# Synthesis, In Vitro Profiling, and In Vivo Evaluation of Benzohomoadamantane-Based Ureas for Visceral Pain: A New Indication for Soluble Epoxide Hydrolase Inhibitors

**DOI:** 10.1021/acs.jmedchem.2c00515

**Published:** 2022-10-12

**Authors:** Sandra Codony, José M. Entrena, Carla Calvó-Tusell, Beatrice Jora, Rafael González-Cano, Sílvia Osuna, Rubén Corpas, Christophe Morisseau, Belén Pérez, Marta Barniol-Xicota, Christian Griñán-Ferré, Concepción Pérez, María Isabel Rodríguez-Franco, Antón L. Martínez, M. Isabel Loza, Mercè Pallàs, Steven H. L. Verhelst, Coral Sanfeliu, Ferran Feixas, Bruce D. Hammock, José Brea, Enrique J. Cobos, Santiago Vázquez

**Affiliations:** † Laboratori de Química Farmacèutica (Unitat Associada al CSIC), Facultat de Farmàcia i Ciències de l’Alimentació, and Institute of Biomedicine (IBUB), Universitat de Barcelona, Av. Joan XXIII, 27-31, Barcelona 08028, Spain; ‡ Animal Behavior Research Unit, Scientific Instrumentation Center, Parque Tecnológico de Ciencias de la Salud, University of Granada, Armilla, Granada 18100, Spain; § CompBioLab Group, Departament de Química and Institut de Química Computacional i Catàlisi (IQCC), Universitat de Girona, C/ Maria Aurèlia Capmany 69, Girona 17003, Spain; ∥ Department of Pharmacology, Faculty of Medicine and Biomedical Research Center (Neurosciences Institute), Biosanitary Research Institute ibs.GRANADA, University of Granada, Avenida de la Investigación 11, Granada 18016, Spain; ⊥ Institució Catalana de Recerca i Estudis Avançats (ICREA), Barcelona 08010, Spain; # Institute of Biomedical Research of Barcelona (IIBB), CSIC and IDIBAPS, Barcelona 08036, Spain; ¶ Department of Entomology and Nematology and Comprehensive Cancer Center, University of California, Davis, California 95616, United States; ∇ Department of Pharmacology, Therapeutics and Toxicology, Institute of Neurosciences, Autonomous University of Barcelona, Bellaterra, Barcelona 08193, Spain; ○ Laboratory of Chemical Biology, Department of Cellular and Molecular Medicine, KU Leuven—University of Leuven, Herestraat 49 box B901, Leuven 3000, Belgium; ⧫ Pharmacology Section, Department of Pharmacology, Toxicology and Therapeutic Chemistry, Faculty of Pharmacy and Food Sciences, Institute of Neuroscience, University of Barcelona (NeuroUB), Av. Joan XXIII 27-31, Barcelona 08028, Spain; †† Institute of Medicinal Chemistry, Spanish National Research Council (CSIC), C/Juan de la Cierva 3, Madrid 28006, Spain; ‡‡ Drug Screening Platform/Biofarma Research Group, CIMUS Research Center, University of Santiago de Compostela (USC), Santiago de Compostela 15782, Spain; §§ Leibniz Institute for Analytical Sciences ISAS, AG Chemical Proteomics, Otto-Hahn-Str. 6b, Dortmund 44227, Germany

## Abstract

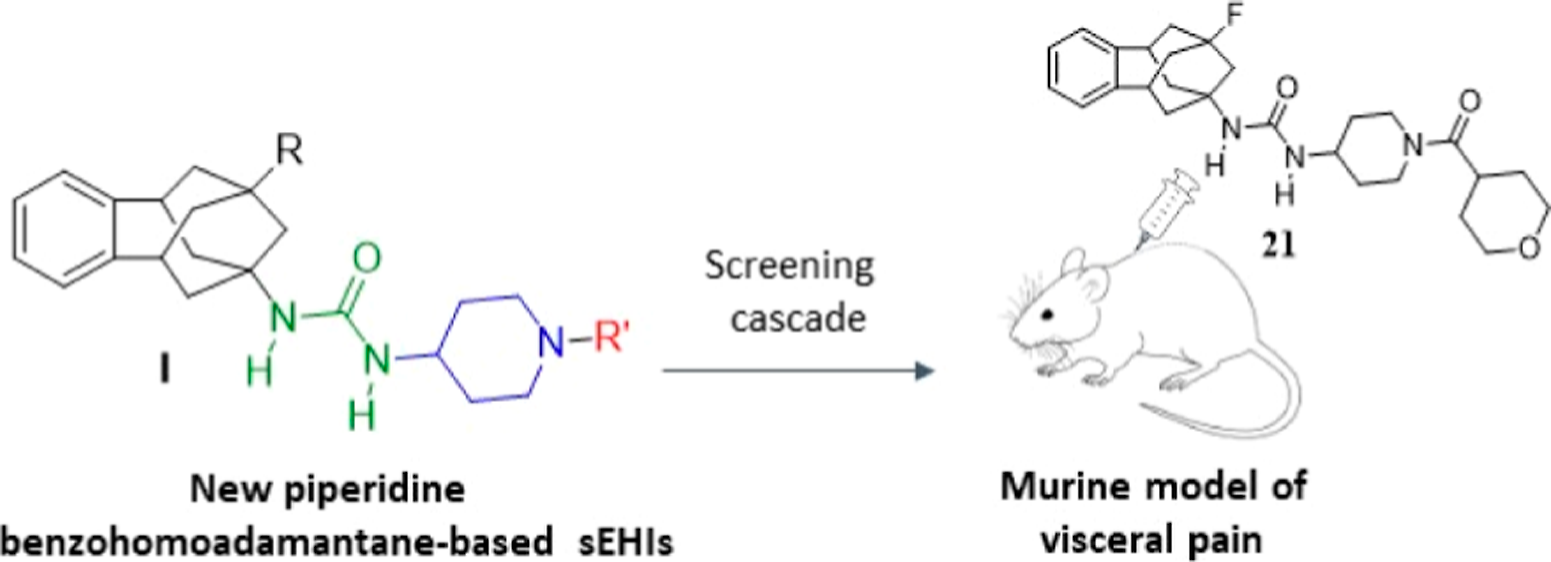

The soluble epoxide hydrolase (sEH) has been suggested as a pharmacological target for the treatment of several diseases, including pain-related disorders. Herein, we report further medicinal chemistry around new benzohomoadamantane-based sEH inhibitors (sEHI) in order to improve the drug metabolism and pharmacokinetics properties of a previous hit. After an extensive in vitro screening cascade, molecular modeling, and in vivo pharmacokinetics studies, two candidates were evaluated in vivo in a murine model of capsaicin-induced allodynia. The two compounds showed an anti-allodynic effect in a dose-dependent manner. Moreover, the most potent compound presented robust analgesic efficacy in the cyclophosphamide-induced murine model of cystitis, a well-established model of visceral pain. Overall, these results suggest painful bladder syndrome as a new possible indication for sEHI, opening a new range of applications for them in the visceral pain field.

## 1 Introduction

**Figure 1:**
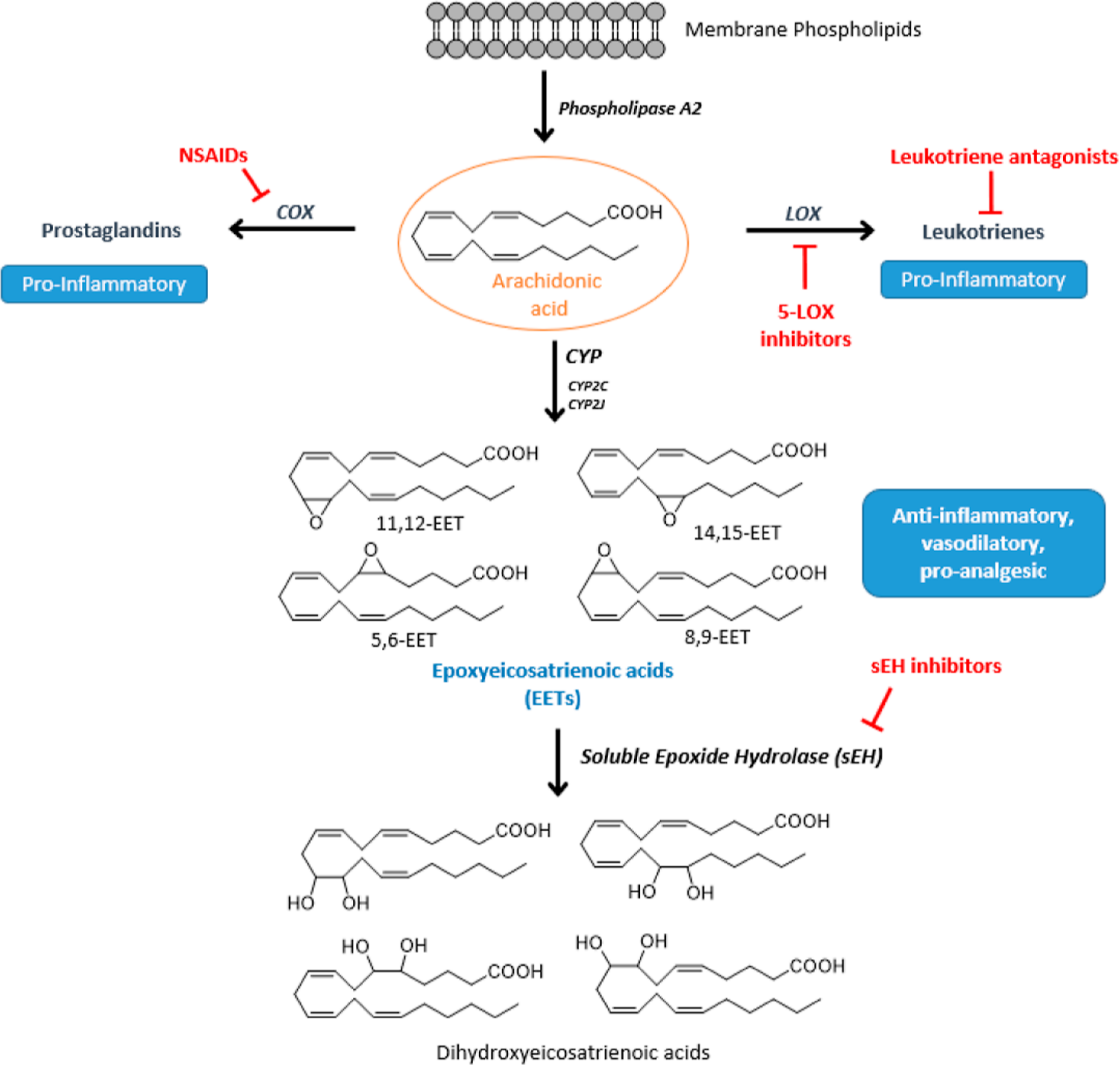
Simplified AA cascade.

Arachidonic acid (AA) is an essential ω-6 20 carbon polyunsaturated fatty acid that is abundant in the phospholipids of cellular membrane. In response to a stimulus, phospholipase A2 promotes its cleavage from the membrane and release into the cytosol, where it can be metabolized, leading to different classes of eicosanoids via three pathways (Figure 1).^1,2^ The cyclooxygenase (COX) pathway catalyzes the production of prostaglandins, prostacyclins, and thromboxanes, endowed with inflammatory properties. The lipoxygenase (LOX) pathway generates leukotrienes, which play a significant part in the onset of asthma, arthritis, allergy, and inflammation.^3^ Both pathways have been extensively studied and targeted pharmaceutically.^4−6^ More recently, increasing attention is being paid to the third branch of the AA cascade, the cytochrome P450 (CYP) pathway that notably converts AA to epoxyeicosatrienoic acids (EETs).^7^ EETs exhibit anti-hypertensive, anti-inflammatory, and anti-nociceptive properties,^8^ but they are rapidly degraded by the soluble epoxide hydrolase (sEH, EPHX2, E.C. 3.3.2.10) to the less active or inactive dihydroxyeicosatrienoic acids (DHETs).

Therefore, sEH inhibition may lead to elevated levels of EETs thereby maintaining their beneficial properties.^9,10^ Indeed, the use of selective sEH inhibitors (sEHI) in vivo models resulted in an increase of EETs levels and the reduction of blood pressure and inflammatory and pain states. Thus, sEH has been suggested as a pharmacological target for the treatment of several diseases, including pain-related disorders.^11−16^

**Figure 2:**
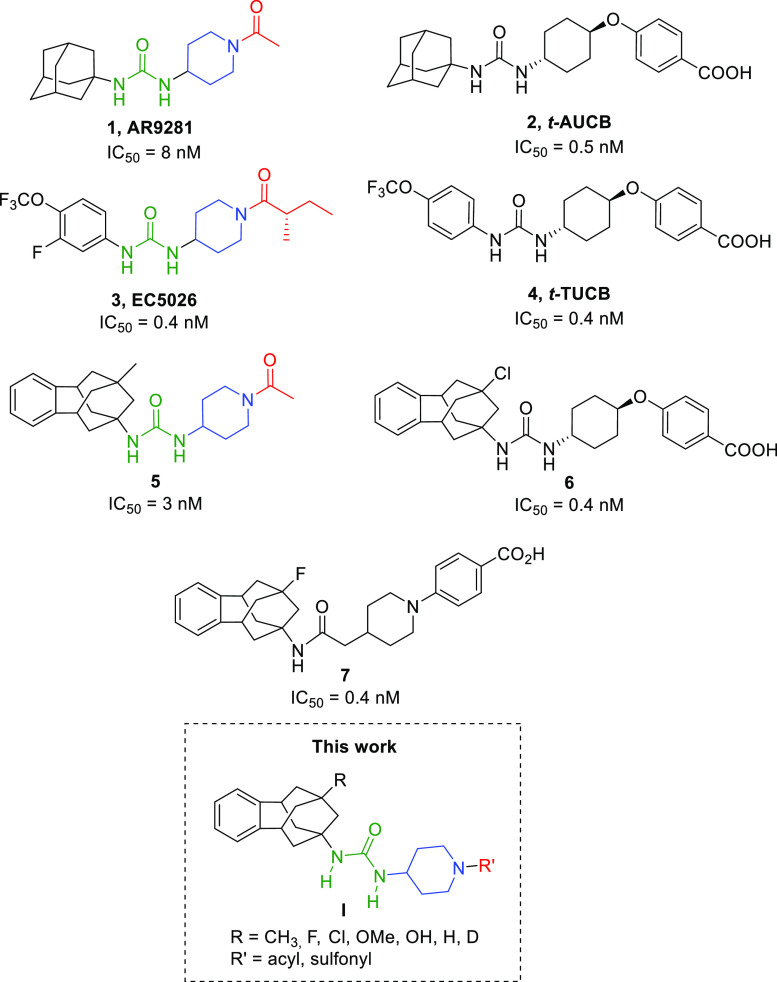
Structures and IC_50_ values in the human sEH of AR9281, **1**, *t*-AUCB, **2**, EC5026, **3**, *t*-TUCB, **4**, **5**, **6**, and **7** and general structure, **I**, of the new derivatives reported on this work.

Given that sEH presents a hydrophobic pocket, several potent sEHI developed in the last years feature an adamantane moiety or an aromatic ring in their structure, such as AR9281, **1**, and EC5026, **3**, two of the sEHI that have reached clinical trials.^17,18^ The first to enter was the adamantane-based AR9281, by Arête Therapeutics, for the treatment of hypertension in diabetic patients. However, it failed largely because of its poor pharmacokinetic properties but also poor target residence time on sEH and only moderate potency on the target.^17^ Very recently, EicOsis has replaced the adamantane moiety of AR9281 by an aromatic ring for its drug candidate EC5026, currently in phase 1 clinical trials for the treatment of neuropathic pain.^18^ Interestingly, both clinical candidates present similar structures: a left-hand side (lhs) hydrophobic moiety (black), a urea group (green), a piperidine residue (blue), and a right-hand side (rhs) acyl group (red). Also, EicOsis is currently advancing the analogue *t*-TUCB, **4**, for veterinary clinical trials (Figure 2).^19^

Our recent observation that the lipophilic cavity of the enzyme is flexible enough to accommodate polycyclic units larger than adamantane,^20^ led to the discovery of a new family of benzohomoadamantane-based ureas, such as **5** and **6**, endowed with low nanomolar or even subnanomolar potencies (Figure 2).^21^ Further in vitro studies with these compounds demonstrated that while compound **5** presented moderate experimental solubility and very poor stability in human and mouse microsomes, compound **6** was endowed with favorable drug metabolism and pharmacokinetics (DMPK) properties and showed efficacy in an in vivo murine model of acute pancreatitis.^21^

Later on, in an effort for improving the DMPK properties of piperidine **5**, we designed a series of analogues where the urea core was replaced by an amide group. Although most of these amides retained or even improved the inhibitory activity of their urea counterparts at the human and mouse enzymes (e.g., compound **7**, Figure 2), only moderate improvements in microsomal stabilities were found.^22^

Herein, we report further medicinal chemistry around inhibitor **5**. New piperidine derivatives retaining the urea group as the main pharmacophore, different substituents in the C-9 position of the polycyclic scaffold (R in **I**), and a broad selection of substituents at the nitrogen atom of the piperidine (R′ in **I**) were synthesized (Figure 2). After a screening cascade, two selected candidates with highly improved DMPK properties were subsequently studied in the murine model of capsaicin-induced allodynia. Finally, the best compound was evaluated in a murine model of visceral pain.

## 2 Results and Discussion

### 2.1 Design and Synthesis of New sEHI

**Figure 3:**
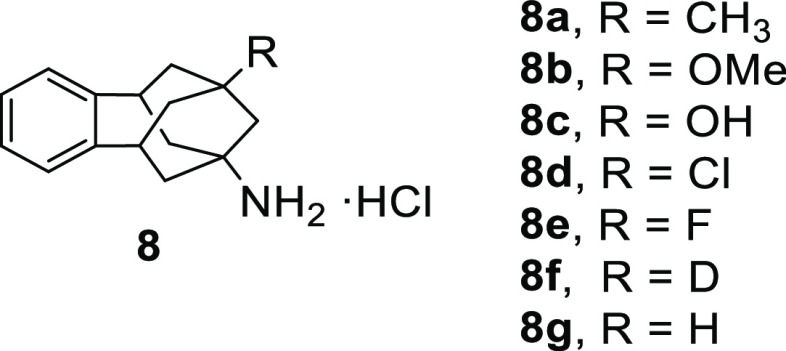
Benzohomoadamantane amines **8a–g** used in this work.

For the preparation of the new sEHI, amines **8a–8g**, previously described by our group, were used as starting materials (Figure 3).^23−26^

**Scheme 1:**
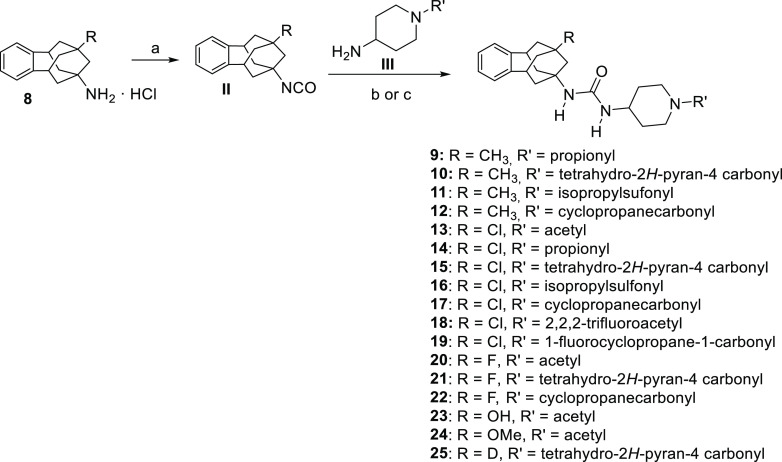
Synthesis of the New sEHI Reagents and conditions: (a) triphosgene, NaHCO_3_, DCM, 30 min; (b) DCM, overnight; and (c) *n*-BuLi, anhyd THF, anhyd DCM, overnight. See the Experimental Section and Supporting Information for further details.

The synthesis of the novel urea-based sEHI was straightforward and involved the reaction of the benzohomoadamantane amines **8a–g** with triphosgene to obtain the corresponding isocyanates **II**, followed by the addition of the required substituted aminopiperidine of general structure **III** to form the final ureas **9–25** (Scheme 1).

All the new compounds were fully characterized through their spectroscopic data and elemental analyses or high-performance liquid chromatography (HPLC)/mass spectrometry (MS) (see the Experimental Section and the Supporting Information for further details).

### 2.2 sEH Inhibition and Microsomal Stability

Compound **5** presented high inhibitory activities against the human and murine enzymes and moderate experimental aqueous solubility (38 μM), but unacceptable stability in human and murine microsomes (Table 1).^21^ Because the acyl chain of piperidine-based sEHI is known to be a suitable position for metabolism,^27^ we decided to explore first new piperidine derivatives replacing the acetyl group of **5** by other fragments selected from previous other series of known sEHI to improve the microsomal stability.^28,29^ Compounds **9–12** were synthesized maintaining the methyl group in the position R of the benzohomoadamantane scaffold **I** and replacing the acetyl group of **5** by the propionyl, tetrahydro-2*H*-pyran-4-carbonyl, isopropylsufonyl, and cyclopropanecarbonyl groups, respectively (Scheme 1). The inhibitory activity against the human and murine enzymes of the new ureas was evaluated, as well as their stabilities in human and mouse microsomes (Table 1).

**Table 1:**
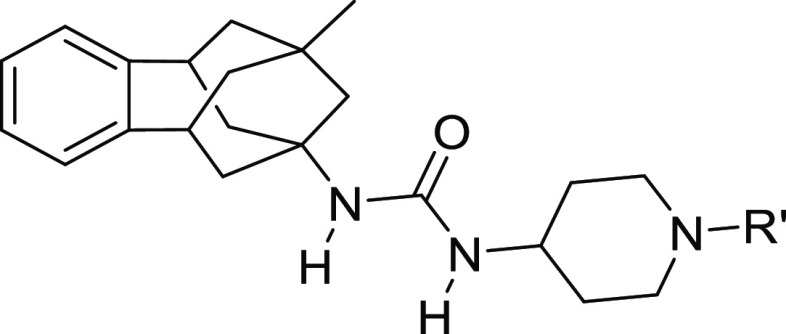

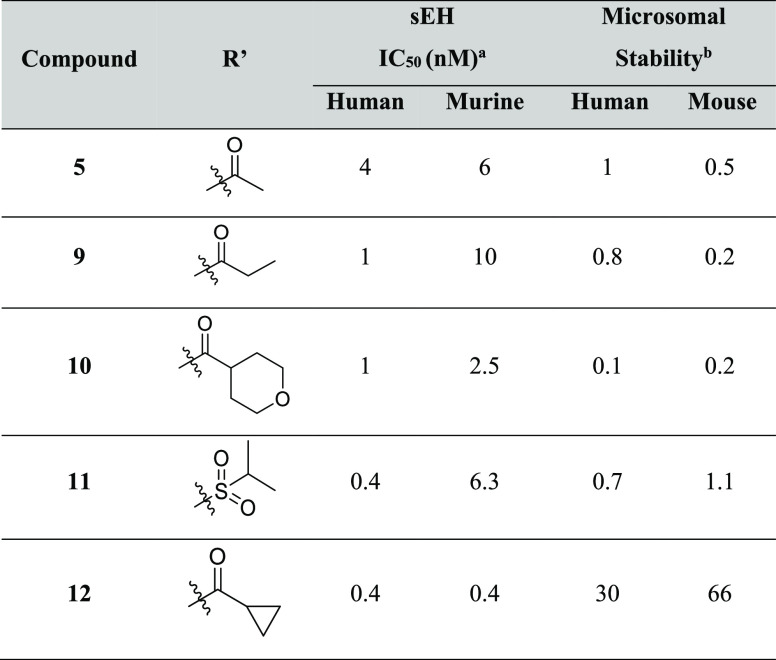
IC_50_ in Human and Murine sEH, and Microsomal Stability Values of **5** and the New sEHI **9–12**

a Reported IC_50_ values are the average of three replicates. The fluorescent assay as performed here has a standard error between 10 and 20%, suggesting that differences of twofold or greater are significant. Because of limitations of the assay, it is difficult to distinguish among potencies <0.5 nM.^30^

b Percentage of remaining compound after 60 min of incubation with pooled human and mouse microsomes in the presence of NADPH at 37 °C.

Gratifyingly, regardless of the substituent of the piperidine ring, all the compounds showed potency in the low nanomolar or even subnanomolar ranges in both the human and murine enzymes (Table 1). Indeed, the most potent compound, **12**, presented inhibitory activities in the subnanomolar range for both enzymes. However, except for **12**, the microsomal stability of these new ureas was very poor and not improved from that of **5** (Table 1).

Consequently, we moved to another strategy for improving the microsomal stability of the compounds, by exploring the C-9 position of the benzohomoadamantane scaffold, replacing the methyl group in **5** and **9–12** by other substituents, such as halogen atoms or polar groups. The potency of these compounds was measured against the human and murine enzymes (Table 2). On the one hand, as expected considering that the catalytic center of sEH is highly hydrophobic, the compounds bearing a polar group in C-9, **23**, and **24**, presented higher IC_50_ values than **5**. Of note, the most important drop in the inhibitory activity was produced by the replacement of the methyl group of **5** by the polar hydroxyl group, compound **23**. On the other hand, when the methyl group was replaced by chlorine or fluorine atoms, the inhibitory activities against the human and murine enzymes were maintained or even improved, as most of them presented IC_50_ values in the low nanomolar or the subnanomolar range (Table 2).

**Table 2:**
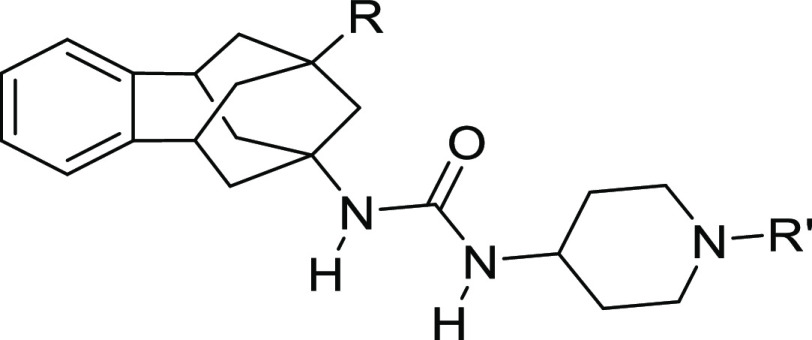

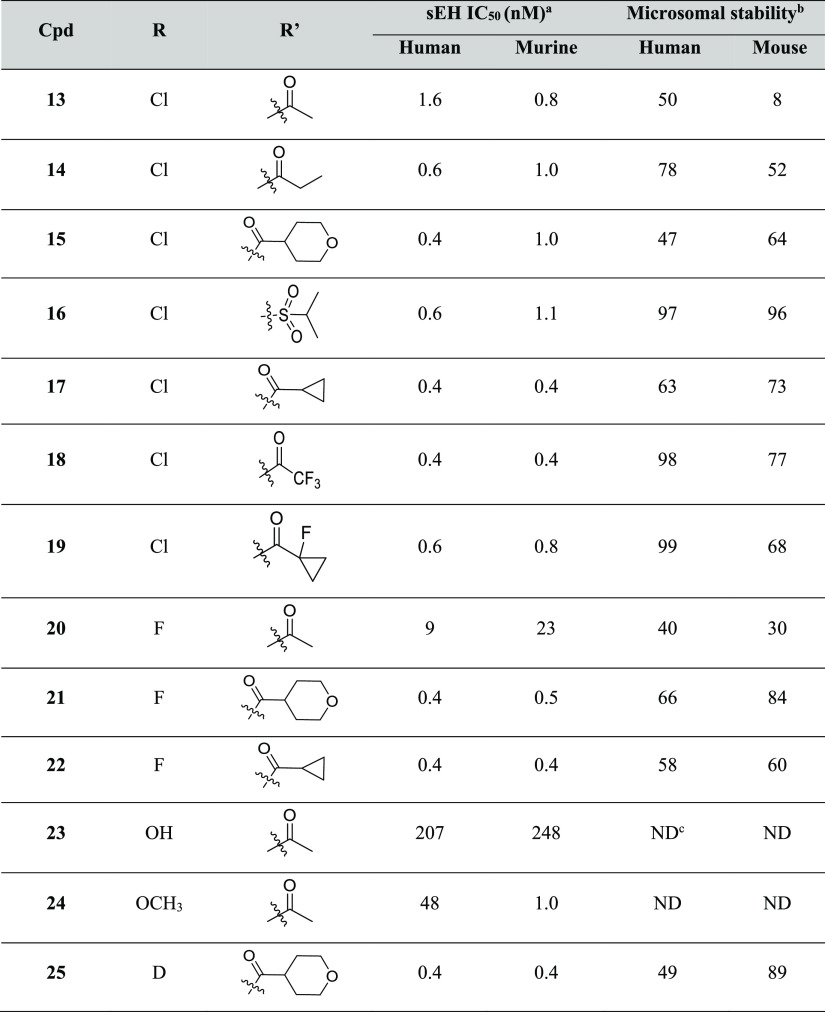
IC_50_ in Human and Murine sEH and Microsomal Stability Values of **13–25**

a Reported IC_50_ values are the average of three replicates. The fluorescent assay as performed here has a standard error between 10 and 20%, suggesting that differences of twofold or greater are significant. Because of limitations of the assay, it is difficult to distinguish among potencies <0.5 nM.^30^

b Percentage of remaining compound after 60 min of incubation with pooled human and mouse microsomes in the presence of NADPH at 37 °C.

c ND: not determined.

Next, the microsomal stability of the most potent compounds was evaluated. Pleasingly, all the compounds featuring halogen atoms in the R position of the benzohomoadamantane scaffold presented better stabilities in human and mice microsomes than their methyl counterparts (Table 2). Especially, the chlorinated compounds **16**, **18**, and **19** exhibited excellent microsomal stabilities in the two species.

### 2.3 In Silico Study: Molecular Basis of Benzohomoadamantane/Piperidine-Based Ureas as sEH Inhibitors

**Figure 4:**
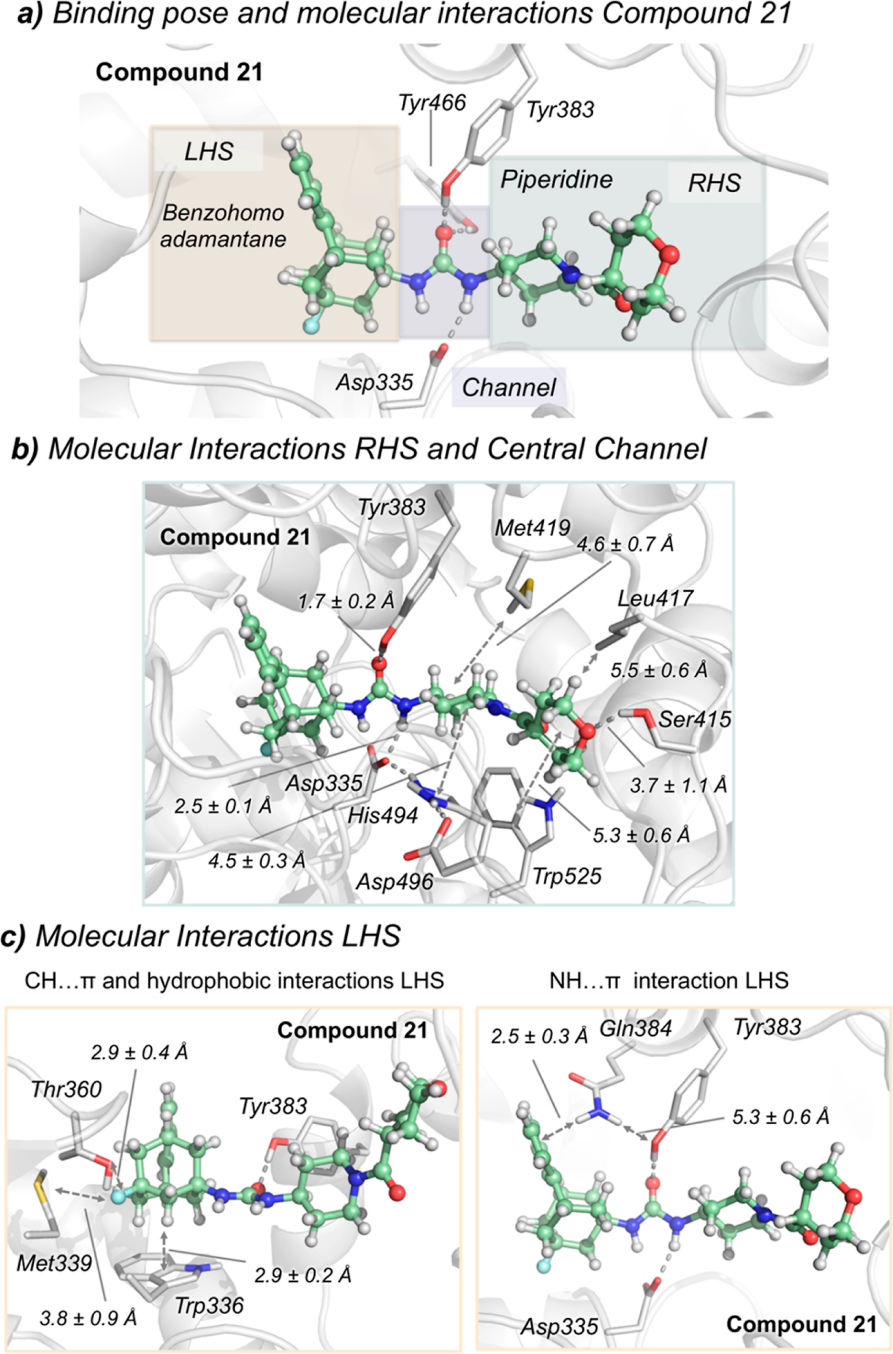
(a) Representative structure of compound **21** bound in the active site of sEH obtained from the most visited conformations along MD simulations. PDB ID 5AM3 has been used as the starting point for MD simulations. The benzohomoadamantane moiety occupies the lhs pocket while the piperidine group is placed in the rhs pocket. The central urea unit establishes hydrogen bonds with Asp335, Tyr466, and Tyr383. (b) Most relevant molecular interactions in the rhs. Average distances (in Å) obtained from three replicas of 500 ns of MD simulations are represented. Hydrogen bonds between the oxygens of the tetrahydropyran group of **21** and the hydrogen of the OH group of Ser415 is shown. The hydrophobic interaction average distances are computed between the terminal heavy atom of amino acid side chains and the centroid of each ring. Hydrogen bond distances between the carboxylic group of the catalytic Asp335 and the amide groups of the inhibitor and the distance between the carbonyl group of the urea inhibitor and the OH group of Tyr383 and Tyr466 residues. (c) Most relevant molecular interactions in the lhs. Average distances (in Å) obtained from the three replicas of 500 ns of MD simulations are represented. The CH−π interaction is calculated between the hydrogens of the benzohomoadamantane unit and the centroid of the benzoid ring of Trp336. The NH−π interaction is monitored between the amide hydrogen of Gln384 and the center of the aromatic ring of the benzohomoadamantane scaffold.

Next, the mechanism of binding of two compounds with high inhibitory activity, that is, **15** (R = Cl, R′ = tetrahydro-2*H*-pyran-4-carbonyl) and **21** (R = F, R′ = tetrahydro-2*H*-pyran-4-carbonyl), was investigated with molecular dynamics (MD) simulations. sEHs present a flexible L-shaped active site pocket divided into three regions: the lhs and the rhs pockets that are connected by a central narrow channel defined by catalytic residues Asp335, Tyr383, and Tyr466 (see Figure 4). Recently, we showed that bulky benzohomoadamantane groups occupy the lhs in urea-based sEHIs that present both adamantyl and phenyl moieties, for example, compound **6**.^21^ However, available X-ray structures of sEH in complex with piperidine-based ureas show that the piperidine group can also occupy the lhs.^31^ To determine the preferred binding mode of **15** and **21** that present both benzohomoadamantane and piperidine groups, we performed conventional MD simulations starting from two possible orientations in the sEH active site predicted by molecular docking calculation (see the Experimental Section): (a) with the benzohomoadamantane in the lhs and piperidine in the rhs (see Figure 4a, similar to adamantyl based-urea in PDB 5AM3) and (b) the piperidine group is placed in lhs while benzohomoadamantane occupies rhs (similar to piperidine based-urea in PDB 5ALZ).^31^ From these MD simulations, the binding affinity of **15** and **21** was estimated with molecular mechanics with generalized Born and surface area solvation (MM/GBSA) calculations showing that the orientation shown in Figure 4a is −5.7 and −10.2 kcal/mol more stable than the opposite orientation for compounds **15** and **21**, respectively (see Table S2). When the benzohomoadamantane occupies the lhs and the piperidine the rhs, both compounds present similar absolute binding affinities (−68.0 and −69.4 kcal/mol for **15** and **21**, respectively), which is in line with the similar IC_50_ values. To corroborate these results, accelerated MD (aMD) simulations were performed to completely reconstruct the binding pathway of compound **15** into the sEH active site pocket (see Movie S1, Figure S1, and Experimental Section). This strategy is frequently used to predict substrate and inhibitor binding pathways in enzymes.^32,33^ Spontaneous binding aMD simulations show how the inhibitor is recognized in the lhs pocket by the benzohomodamantane scaffold and then extends through the sEH binding site accommodating the benzohomoadamantane moiety in the lhs, while the piperidine counterpart lays in the rhs pocket. Considering these results, we conclude that the orientation shown in Figure 4a is the preferred binding mode of compounds **15** and **21**.

To understand in more detail the molecular basis of the inhibitory mechanism of benzohomoadamantane/piperidine-based ureas **15** and **21**, the non-covalent interactions between the selected inhibitors and the active site residues of sEH were studied (see Figure 4 for compound **21** and Figure S2 for compound **15**). MD simulations show that the inhibitor is retained in the active site through three strong hydrogen bond interactions between the urea moiety and the central channel residues Asp335, Tyr383, and Tyr466 (see Figures 4b and S3). In the rhs pocket, the piperidine group is stabilized through persistent hydrophobic interactions with His494 and Met419, while the tetrahydro-2*H*-pyran moiety is retained by the side chains of Leu417 and Trp525. The oxygen of tetrahydro-2*H*-pyran ring establishes transient hydrogen bonds with Ser415 and is relatively solvent exposed (see Figure 4b). In the lhs pocket, the orientation of the benzohomoadmantane moiety is directed by the NH···π interaction between the Gln384 and the aromatic ring of the polycyclic scaffold, which is maintained along the MD simulations. Additionally, hydrophobic interactions are established with the side chains of Met339 and Trp336. This extensive network of hydrophobic interactions and hydrogen bonds in the sEH pocket is key to recognize and bind the inhibitor in the active site.

Introducing a polar hydroxy group in the polycyclic scaffold (compound **23**) significantly decreases the resulting inhibitory activity (see Table 2). To determine the molecular basis of this drop in activity, the binding modes of compounds **13** (R = Cl and R′ = acetyl and IC_50_ = 1.6 nM) and **23** (R = OH and R′ = acetyl and IC_50_ = 207 nM) were compared with MD simulations. The incorporation of OH in the polycyclic scaffold causes a series of rearrangements in the lhs pocket that destabilize the inhibitor bounds with the enzyme in the active site (see Figure S4). In particular, the Thr360 side chain establishes a hydrogen bond with the oxygen of the hydroxyl substituent of compound **23** that induces the rotation of the benzohomoadamantane scaffold in the lhs pocket. This breaks the NH−π interaction between Gln384 and the aromatic ring of **23** providing more flexibility to the benzohomadamantane moiety as compared to **13**, **15**, and **21**, which may be related to the decreased activity (see Figure S5). In addition, the enhanced dynamism of the polycyclic scaffold allows the transient entrance of few water molecules into the lhs pocket (average number of water molecules 0.97 ± 0.96 for **23** and 0.31 ± 0.5 for **21**, see Figure S6). Compound **24** (R = OCH_3_ and R′ = acetyl, IC_50_ = 48 nM) that also present reduced activity shows a similar behavior as **23** (see Figures S5 and S6). Therefore, the above-mentioned results and those previously reported with related compounds,^21^ reveal that the presence of a small, lipophilic group at C-9 of the benzohomodamantane scaffold is key for the stability and activity of benzohomoadamantane-based sEHIs at the molecular level.

### 2.4 Further DMPK Profiling of the Selected Inhibitors

The halogen-substituted sEHI compounds that exhibited outstanding inhibitory activities and had more than 50% of the parent compound unaltered after incubation with human and/or murine microsomes were selected for further evaluation. Solubility, permeability through the blood–brain barrier (BBB), cytotoxicity, and cytochrome inhibition of the selected compounds **14–19**, **21**, **22**, and **25** were experimentally measured. In addition, we evaluated all the synthesized compounds as pan assay interference compounds (PAINS) using SwissADME and FAFDrugs4 web tools.^34,35^ None of them gave positive as PAINS.

While compounds **14**, **16**, **17**, **18**, and **19** exhibited limited solubility, with values lower than 20 μM, compounds **15**, **21**, **22**, and **25** displayed good to excellent solubility values. Additionally, the selected compounds were further tested for predicted brain permeation in the widely used in vitro parallel artificial membrane permeability assay–BBB (PAMPA–BBB) model.^36^ Compounds **14**, **15**, **22**, and **25** showed CNS+ proving their potential capacity to reach CNS, whereas the other compounds presented uncertain BBB permeation (CNS+/−). Next, the cytotoxicity of the new sEHI was tested using the propidium iodide (PI) and MTT assays in SH-SY5Y cells. Interestingly, none of the selected compounds appeared cytotoxic at the highest concentration tested (100 μM) (Table 3).

**Table 3:** Solubility and Permeability (PAMPA–BBB) Values, Cytotoxicity, and Inhibition of Pooled Human Cytochromes P450 Enzymes of Selected sEHI

			cytotoxicity LD_50_ (μM)		cytochrome inhibition^d^^,^^e^		
compound	solubility (μM)^a^	PAMPA–BBB	PI^b^	MTT^c^	CYP 2C9	CYP 2C19	CYP 3A4 (7-BFC)^f^
**14**	18	CNS+	>100	>100	30 ± 4	46 ± 3	44 ± 1
**15**	57	CNS+	>100	>100	34 ± 1	38 ± 4	1 ± 1
**16**	19	CNS+/–	>100	>100	38 ± 1	1.48 μM	18 ± 1
**17**	19	CNS+/–	>100	>100	34 ± 2	1.54 μM	1 ± 1
**18**	16	CNS+/–	>100	>100	54 ± 1	0.63 μM	2 ± 1
**19**	17	CNS+/–	>100	>100	43 ± 3	0.78 μM	3 ± 2
**21**	95	CNS+/–	>100	>100	30 ± 3	32 ± 4	2 ± 2
**22**	92	CNS+	>100	>100	17 ± 2	26 ± 5	1 ± 1
**25**	62	CNS+	>100	>100	12 ± 2	40 ± 1	0.70 μM

a Solubility measured in a 1% DMSO: 99% PBS buffer solution.

b Cytotoxicity tested by PI staining after 24 h incubation in SH-SY5Y cells.

c Cytotoxicity tested by 3-[4,5-dimethylthiazole-2-yl]-2,5-diphenyltetrazolium bromide (MTT) assay after 24 h incubation in SH-SY5Y cells.

d The percent of cytochrome inhibition was tested at 10 μM. IC_50_ was calculated for those compounds that presented >50% of inhibition at 10 μM.

e At 10 μM, all the compounds inhibited <50% the cytochromes CYP1A2, CYP2D6, and CYP3A4 (DBF).

f For the study of CYP3A4, two different substrates were used benzyloxytrifluoromethylcoumarin (BFC) and dibenzylfluorescein (DBF). See the Experimental Section for further details.

Finally, inhibition of several cytochrome P450 enzymes were measured, giving special attention to CYPs 2C19 and 2C9, as these isoforms are two of the main producers of EETs, the substrates of the sEH.^8^ Unfortunately, compounds **16**, **17**, **18**, and **19** inhibited significantly CYP 2C19. In contrast, compounds **14**, **15**, **21**, **22**, and **25** did not significantly inhibit these subfamilies of cytochromes (Table 3). Additionally, CYPs 2D6, 1A2, and 3A4 were also evaluated (Table S3). With the only exception of **25**, which inhibited CYP3A4 in the submicromolar range, all the compounds showed IC_50_ values higher than 10 μM (Tables 3 and S3).

After performing the above-mentioned screening cascade, three compounds, **15**, **21**, and **22**, emerged as the more promising candidates. These compounds exhibited excellent inhibitory activities against the human and murine enzymes, improved metabolic stability, good solubility, and did not significantly inhibit cytochromes. Notwithstanding, hERG inhibition and Caco-2 assays were also performed in order to additionally characterize them. None of the compounds significantly inhibit hERG at 10 μM, and they displayed moderate permeability in Caco-2 cells. Finally, they were tested for selectivity against hCOX-2 and hLOX-5, two enzymes involved in the AA cascade. Gratifyingly, they did not present significant inhibition of these enzymes (Table 4).

**Table 4:** Permeability Values (Caco-2) and Inhibition of the hERG Channel of hLOX-5 and of hCOX-2 of the Selected Compounds **15**, **21**, and **22**

	permeability (Caco-2)					
	papp (nm/s)					
Cpd	A → B	B → A	ER^a^	hERG channel inhibition (% inhib. 10 μM)	IC_50_ hLOX-5 (μM)^b^	IC_50_ hCOX-2 (μM)^c^
**15**	55.6 ± 0.7	171.6 ± 0.5	3.1 ± 0.3	1 ± 2	>100	>10
**21**	32.9 ± 1	301.7 ± 26.5	9.2 ± 0.5	2 ± 1	>100	>10
**22**	26.9 ± 2	235.4 ± 14.5	8.8 ± 0.2	1 ± 2	>100	>10

a The efflux ratio was calculated as ER = (Papp B → A)/(Papp A → B). See the Experimental Section for further details.

b IC_50_ in human LOX-5 (hLOX-5). See the Experimental Section for further details.

c IC_50_ in human COX-2 (*h*COX-2) performed by Eurofins (catalogue reference 4186).

### 2.5 sEH Engagement and Off-Target Profile

**Scheme 2:**
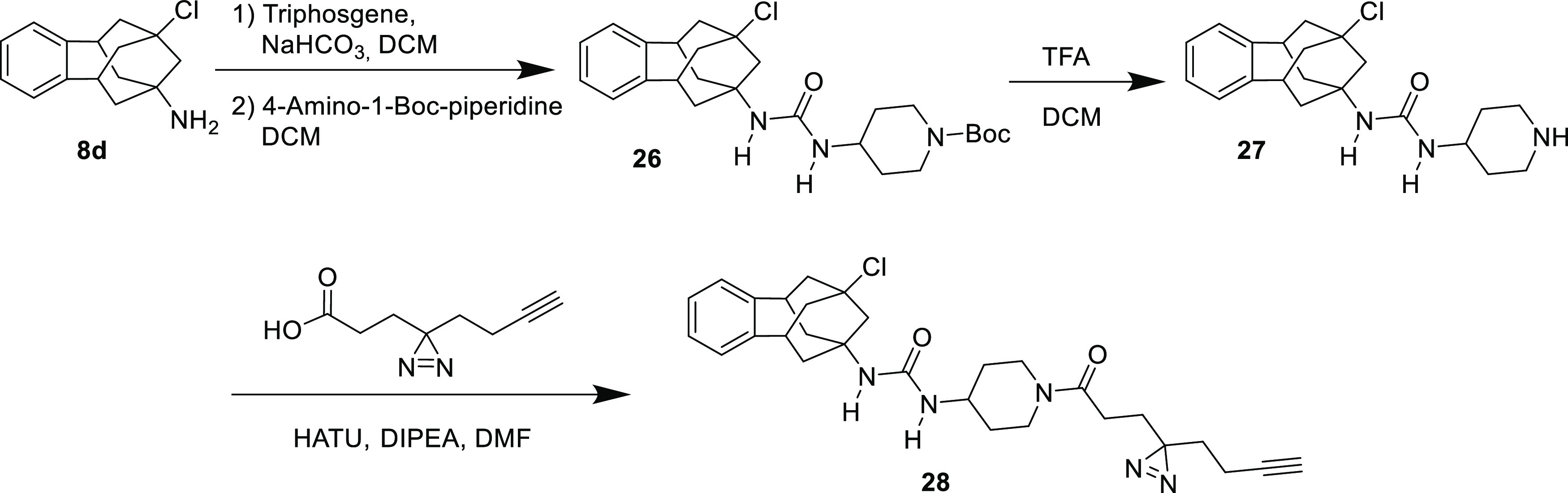
Synthesis of the Probe **28** See the Experimental Section for details.

Compound **28** was designed as a chemical probe with the objective to disturb the parent compound structure as little as possible. Important in this design was the knowledge that the piperidine nitrogen atom can be substituted without loss of biological activity. Therefore, a butynyl diazirinyl propionic acid minimalistic linker was coupled, via a straightforward amide coupling reaction, to the piperidine nitrogen of **27**, in turn obtained from **8d** through urea formation and Boc-removal (Scheme 2). The probe **28** was found to be a potent inhibitor with IC_50_ of 0.5 and 0.4 nM, for the human and mouse enzymes, respectively.

**Figure 5:**
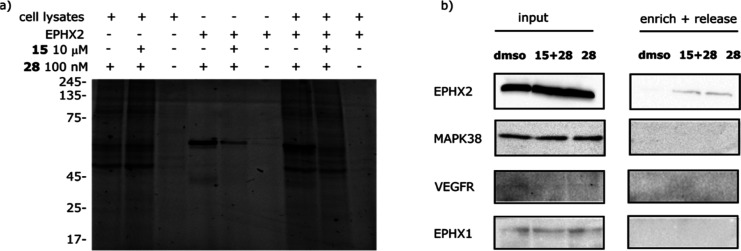
Target engagement and off-target profile of **28** in HEK293T cell lysates. (a) Fluorescence scan showing probe **28** labeling pattern in lysates, purified EPHX2- and EPHX2-spiked lysates, revealing that EPHX2 is the only target visibly outcompeted by the parent compound **15** and hence, the only target with high occupancy (Coomassie-stained gel in Figure S11). (b) Western blot analysis of selected proteins further confirms EPHX2 target engagement by **28** and proves that neither EPHX1, MAPK38, nor VEGFR are targeted by this compound. Compound **28** used at 10 μM and compound **15** used at 100 μM.

Next, we tested whether probe **28** could covalently bind endogenously expressed human sEH in a complex proteome. Hence, photoaffinity labeling was followed by incorporation of an azide-TAMRA-Biotin tag via copper(I) azide alkyne cycloaddition (CuAAc). This tag allows both visualization and isolation of the probe’s protein targets. A fluorescent band at 72 KDa was identified as sEH via immunoblotting (Figures 5, S7, S9 and S10).

Once the probe engagement of EPHX2 was confirmed, we determined the minimal probe labeling concentration using purified recombinant human EPHX2 (Figure S8). The minimal probe concentration was found to be 100 nM, which was then used to get insights in the selectivity of the probe **28** and compound **15**. Although it was observed that probe **28** labeled multiple bands, competition with the parent compound **15** shows competition of only EPHX2, illustrating that this is the sole target with high occupancy and that the other bands are non-specific labeling events by the probe (Figure 5). To further confirm the selective character of **15**, we wanted to exclude p38 mitogen-activated protein kinase (p38 MAPK) and pro-angiogenic kinase vascular endothelial growth factor receptor-2 (VEGFR2) as targets because some urea-based sEHI are reported to show cross-reactivity with these proteins.^37−39^ In addition, we also aimed to exclude membrane bound microsomal epoxide hydrolase as a possible off-target.^40^ To this end, we performed pull-down experiments and immunoblotting with specific antibodies. These experiments confirmed that none of these proteins are targets of **28**, underlining its selectivity (Figure 5b).

### 2.6 Pharmacokinetic Study of Compounds **15** and **21**

Overall, compounds **15** and **21**, with similar DMPK properties and structures, were selected for in vivo studies. First, a study was conducted in order to determine the pharmacokinetic profile in the plasma of compounds **15** and **21** when administered by a subcutaneous (sc) route at a single dose of 5 mg/kg. As shown in Table 5, absorption of **21** is fast, reaching *C*_max_ (19.1 μg/mL) at 15 min after dosing. The compound disappeared from the plasma progressively and half-life (HL) was calculated to be around 0.7 h. In the case of **15**, *C*_max_ (1.2 μg/mL) was 15 times lower than that of **21**, however, showing a higher HL (3.4 h). For both compounds, the narrow differences in AUC_0_^*t*^ and AUC_0_^∞^ showed complete exposure and good bioavailability. Although **21** demonstrated better bioavailability characteristics than **15** both compounds were subsequently evaluated in vivo efficacy studies.

**Table 5:** Pharmacokinetic Parameters in Male CD1 Mice for Compounds **15** and **21** After 5 mg/kg sc Administration^a^

compound	Dose	HL (h)	*T*_max_ (h)	*C*_max_ (μg/mL)	AUClast (μg*h/mL)	AUCINF (μg*h/mL)
**15**	5 mg/Kg	3.42	0.75	1.2	2.4	2.5
**21**	5 mg/Kg	0.70	0.25	19.1	13.5	13.6

a See the Experimental Section and Tables S4 and S5 and Figures S12 and S13 in the Supporting Information.

### 2.7 In Vivo Efficacy Studies

**Figure 6:**
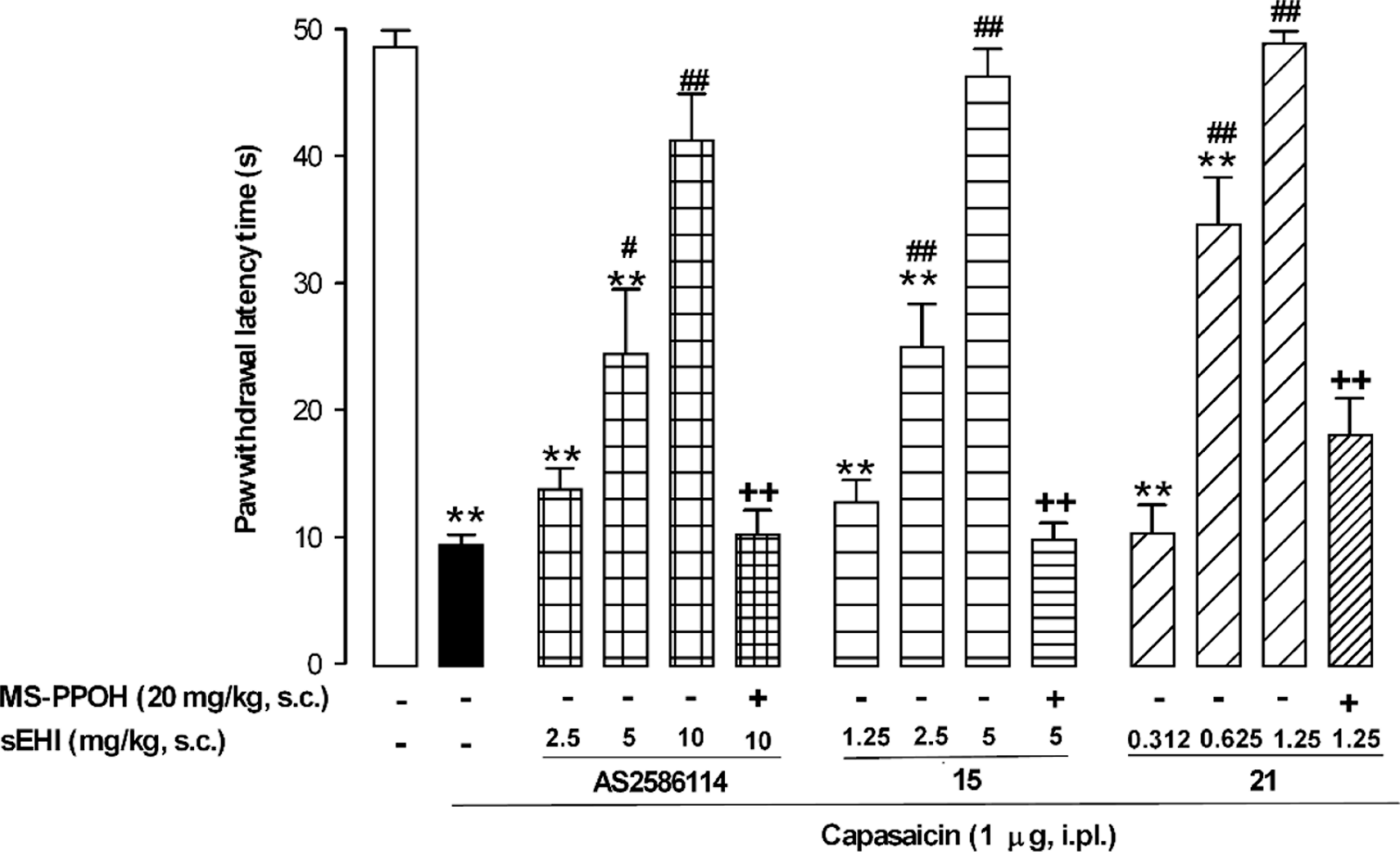
Reduction of capsaicin-induced secondary mechanical hypersensitivity in mice by the systemic administration of AS2586114, and compounds **15** and **21**, is due to sEH inhibition. The data shown represent the effect of the sc administration of AS2586114, **15**, and **21** administered alone or associated with the CYP450 oxidase inhibitor MS-PPOH (sc) on paw withdrawal latency in mice-treated intra-plantarly (i.pl.) with capsaicin. Each bar and vertical line represent the mean ± SEM of the values obtained in 8–10 animals. Statistically significant differences: **p < 0.01 between nonsensitized mice (open bar) and the other experimental groups; #*p* < 0.05 and ##*p* < 0.01 between capsaicin-treated mice injected with the sEHI or their solvent (black bar); ++*p* < 0.01 sEHI-treated mice associated or not with MS-PPOH (one-way ANOVA followed by Student–Newman–Keuls test).

A first in vivo efficacy study was performed in a capsaicin-induced secondary mechanical hypersensitivity (allodynia) model in mice. It is well known that the increase in sensitivity to mechanical stimulation in the area surrounding capsaicin injection results from central sensitization,^41^ which is a key process in chronic pain development and maintenance.^42^ In our experimental conditions, mice markedly decreased their paw withdrawal latency to mechanical stimulation after capsaicin administration (Figure 6), denoting the development of mechanical allodynia. The sc administration of the prototypic, brain-penetrant,^43−46^ sEHI AS2586114 induced a dose-dependent reversion of the capsaicin-induced mechanical hypersensitivity reaching a full reversal of sensory hypersensitivity at 10 mg/kg (Figure 6). The sc administration of compounds **15** and **21** fully inhibited mechanical hypersensitivity in a dose-dependent manner and with a much higher potency than AS2586114, reaching full reversal of sensory gain with 5 mg/kg for compound **15** and even with a dose as low as 1.25 mg/kg for compound **21** (Figure 6), in spite of its limited predicted BBB permeability (as previously commented). Importantly, the administration of *N*-methanesulfonyl-6-(2-proparyloxyphenyl)hexanamide (MS-PPOH), an inhibitor of microsomal CYP450s, which is responsible for the production of EETs,^47^ fully abolished the effect of not only AS2586114 but also those induced by compounds **15** and **21** (Figure 6). These results strongly suggest that the three tested compounds induced the reversal of capsaicin-induced mechanical hypersensitivity through the in vivo inhibition of sEH.

Given that the tested compounds induced ameliorative effects on this behavioral model of central sensitization attributable to sEH inhibition, we tested the effect of compound **21** (the most potent compound among the sEHI evaluated), in a model of pathological pain. Specifically, cyclophosphamide (CTX)-induced cystitis because it has been used as a model of interstitial cystitis/bladder pain syndrome,^48^ and it is known that pain induced by this disease has a strong component of central sensitization in both humans and rodents.^49,50^

**Figure 7:**
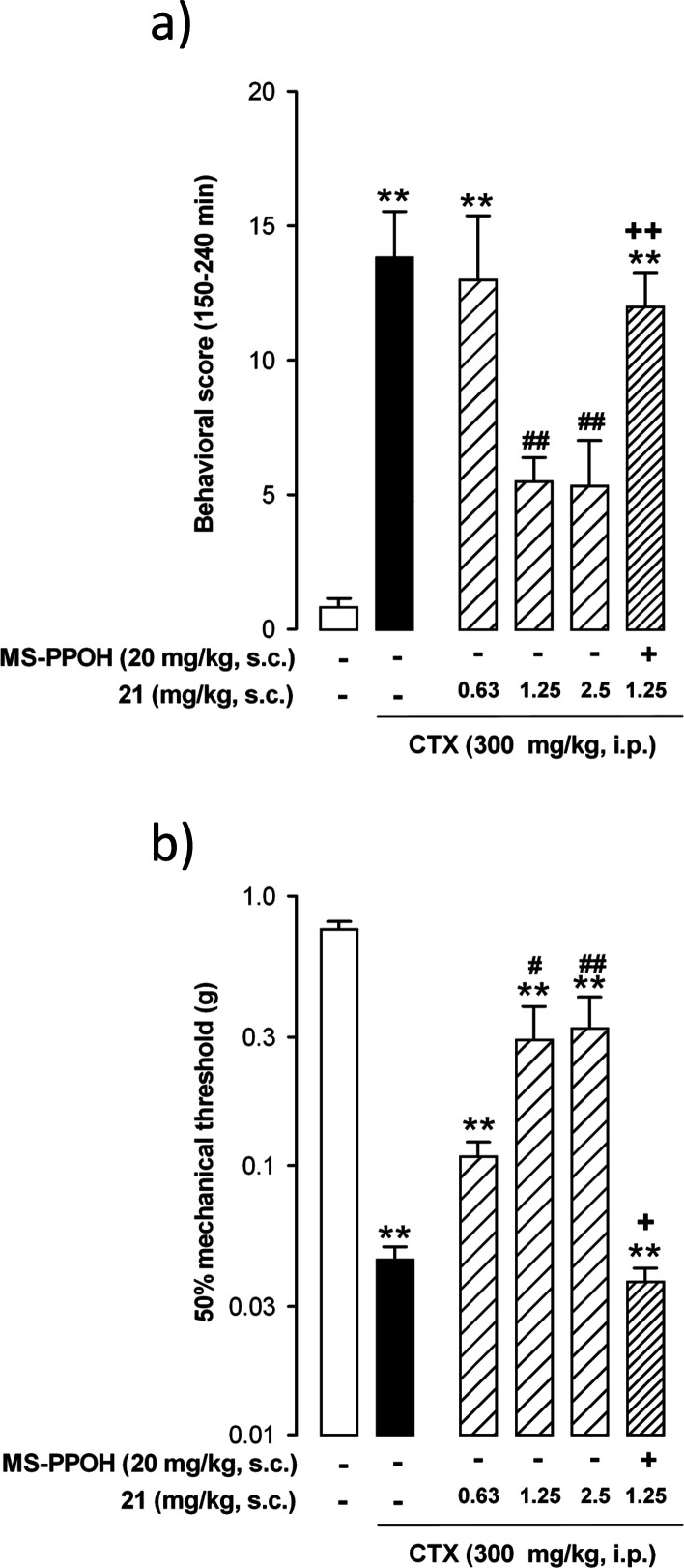
Effects of compound **21** on pain-related behaviors and referred mechanical hyperalgesia induced by CTX. (a) Behavioral score was recorded at 30 min intervals over the 150–240 min observation period after the intraperitoneal (ip) injection of (CTX, 300 mg/kg) or its vehicle. (b) 50% mechanical threshold was evaluated by stimulation of the abdomen with von Frey filaments at 240 min after the administration CTX or its vehicle and was used as an index of referred hyperalgesia. Each bar and vertical line represents the mean ± SEM of values obtained in at least six animals per group. Statistically significant differences: **p < 0.01, between nonsensitized mice (open bar) and the other experimental groups; #*p* < 0.05, ##*p* < 0.01 between CTX-treated mice injected with the sEHI or their solvent (black bar); ++*p* < 0.01 mice injected with compound **21** associated or not with MS-PPOH (one-way ANOVA followed by Student–Newman–Keuls test).

In our experimental conditions, mice treated with CTX showed a significant increase in the pain behavioral score in comparison to mice treated with the vehicle (Figure 7a). The sc administration of compound **21** (0.63–2.5 mg/kg) significantly reduced this pain-related score in a dose-dependent manner (Figure 7a). In addition, animals administered with the CTX vehicle showed a marked reduction in their mechanical threshold in the abdomen, denoting the development of referred hyperalgesia (Figure 7b). The sc treatment with compound **21** also reversed, in a dose-dependent manner, the mechanical referred hyperalgesia induced by CTX (Figure 7b). The administration of MS-PPOH fully reversed the effect of compound **21** in either the pain-related behaviors as in referred hyperalgesia (Figure 7a,b, respectively), mirroring the results obtained on capsaicin-induced secondary hyperalgesia and suggesting that compound **21** exerted its in vivo effects on pain through sEH inhibition. To our knowledge, there are no previous studies exploring the role of sEHI on visceral pain. Therefore, our results suggest interstitial cystitis/pain bladder syndrome as a possible new indication for inhibitors of sEH.

## 3 Conclusions

sEH is a suitable target for several inflammatory and pain-related diseases. In this work, we report further medicinal chemistry around new benzohomoadamantane-based piperidine derivatives, analogues of the clinical candidates AR9281 and EC5026. The introduction of a halogen atom in the position C-9 of the benzohomoadamantane scaffold led to very potent compounds with improved DMPK properties. The in vitro profiling of these new sEHI (solubility, cytotoxicity, metabolic stability, CYP450s, hLOX-5, hCOX-2, and hERG inhibition) allowed one to select two suitable candidates for in vivo efficacy studies. The administration of compounds **15** and **21** reduced pain in the capsaicin-induced murine model of allodynia in a dose-dependent manner and outperformed AS2586114. Moreover, compound **21** was tested in a CTX-induced murine model of cystitis, revealing its robust analgesic effect. Hence, this study opens a whole range of applications of the benzohomoadamantane-based sEHIs in pain and likely other fields.

## 4 Experimental Section

### 4.1 Chemical Synthesis

Commercially available reagents and solvents were used without further purification unless stated otherwise. Preparative normal phase chromatography was performed on a CombiFlash Rf 150 (Teledyne Isco) with pre-packed RediSep Rf silica gel cartridges. Thin-layer chromatography was performed with aluminum-backed sheets with silica gel 60 F254 (Merck, ref 1.05554), and spots were visualized with UV light and 1% aqueous solution of KMnO_4_. HPLC purification was performed on a Prominence ultra-fast liquid chromatography system (Shimadzu) using a Waters X-bridge 150 mm C18 prep column with a gradient of acetonitrile in water (with 0.1% trifluoroacetic acid) over 32 min. All compounds showed a sharp melting point and a single spot on TLC. Purity >95% of all final compounds was assessed by the integration of LC chromatograms. Melting points were determined in open capillary tubes with a MFB 595010M Gallenkamp. 400 MHz ^1^H and 100.6 MHz ^13^C NMR spectra were recorded on a Varian Mercury 400 or on a Bruker 400 Avance III spectrometers. 500 MHz ^1^H NMR spectra were recorded on a Varian Inova 500 spectrometer. The chemical shifts are reported in ppm (δ scale) relative to internal tetramethylsilane, and coupling constants are reported in Hertz (Hz). Assignments given for the NMR spectra of selected new compounds have been carried out on the basis of DEPT, COSY 1H/1H (standard procedures), and COSY ^1^H/^13^C (gHSQC and gHMBC sequences) experiments. IR spectra were run on PerkinElmer spectrum RX I, PerkinElmer spectrum TWO, or Nicolet Avatar 320 FT-IR spectrophotometers. Absorption values are expressed as wavenumbers (cm^–1^); only significant absorption bands are given. High-resolution mass spectrometry (HRMS) analyses were performed with an LC/MSD TOF Agilent Technologies spectrometer. The elemental analyses were carried out in a Flash 1112 series Thermo Finnigan elemental microanalyzer (A5) to determine C, H, N, and S. The structure of all new compounds was confirmed by elemental analysis and/or accurate mass measurement, IR, ^1^H NMR, and ^13^C NMR. The analytical samples of all the new compounds, which were subjected to pharmacological evaluation, possessed purity ≥95% as evidenced by their elemental analyses (Table S1) or HPLC/UV. HPLC/UV were determined with a HPLC Agilent 1260 Infinity II LC/MSD coupled to a photodiode array. 5 μL of sample 0.5 mg/mL in methanol/acetonitrile were injected, using an Agilent Poroshell 120 EC-C18, 2.7 μm, 50 mm × 4.6 mm column at 40 °C. The mobile phase was a mixture of A = water with 0.05% formic acid and B = acetonitrile with 0.05% formic acid, with the method described as follows: flow 0.6 mL/min, 5% B–95% A 3 min, 100% B 4 min, and 95% B–5% A 1 min. Purity is given as % of absorbance at 220 nm.

#### 4.1.1 1-(9-Methyl-5,6,8,9,10,11-hexahydro-7*H*-5,9:7,11-dimethanobenzo[9]annulen-7-yl)-3-(1-propionylpiperidin-4-yl)urea (**9**)

To a solution of 9-methyl-5,6,8,9,10,11-hexahydro-7*H*-5,9:7,11-dimethanobenzo[9]annulen-7-amine hydrochloride (464 mg, 1.76 mmol) in DCM (10 mL), saturated aqueous NaHCO_3_ solution (10 mL) and triphosgene (193 mg, 0.65 mmol) were added. The biphasic mixture was stirred at room temperature for 30 min, then the two phases were separated, and the organic layer was washed with brine (5 mL), dried over anhyd Na_2_SO_4_, filtered, and evaporated under vacuum to obtain 1–2 mL of a solution of the isocyanate in DCM. To this solution was added 1-(4-aminopiperidin-1-yl)propan-1-one (350 mg, 2.24 mmol). The reaction mixture was stirred at room temperature overnight and the solvent was evaporated under vacuum to obtain a white solid (741 mg). Column chromatography (SiO_2_, DCM/methanol mixtures) gave urea **9** (597 mg, 83% yield) as a white solid. The analytical sample was obtained by crystallization from hot EtOAc and DCM (300 mg), mp 207–208 °C. IR (NaCl disk): 3357, 2917, 2859, 1644, 1620, 1556, 1493, 1450, 1361, 1344, 1319, 1264, 1221, 1132, 1068, 1024, 971, 949, 758 cm^–1^. ^1^H NMR (400 MHz, CDCl_3_): δ 0.90 (s, 3H, C9–CH_3_), 1.11 (t, *J* = 7.2 Hz, 3H, COCH_2_CH_3_), 1.14 [m, 2H, COCH_2_CH_3_, 5′(3′)-H_ax_], 1.52 [d, *J* = 13.4 Hz, 2H, 10(13)-H_ax_], 1.62 [dd, *J* = 13.4 Hz, *J*′ = 6.0 Hz, 2H, 10(13)-H_eq_], 1.77–1.86 (complex signal, 3H, 8-H_2_, 5′-H_eq_ or 3′-H_eq_), 1.93 [d, *J* = 12.8 Hz, 2H, 6(12)-H_ax_], 2.02 (d, *J* = 12.0 Hz, 1H, 3′-H_eq_ or 5′-H_eq_), 2.12 [dd, *J* = 12.8 Hz, *J*′ = 6.0 Hz, 2H, 6(12)-H_eq_], 2.32 (m, 2H, COCH_2_CH_3_), 2.70 (m, 1H, 2′-H_ax_ or 6′-H_ax_), 3.00–3.12 [complex signal, 3H, 5(11)-H, 6′-H_ax_ or 2′-H_ax_], 3.70–3.77 (complex signal, 2H, 4′-H, 6′-H_eq_ or 2′-H_eq_), 4.47 (d, *J* = 13.6 Hz, 1H, 2′-H_eq_ or 6′-H_eq_), 4.64–4.72 (complex signal, 2H, C7–NH, C4′-NH), 7.02 [m, 2H, 1(4)-H], 7.05 [m, 2H, 2(3)-H]. ^13^C NMR (100.6 MHz, CDCl_3_): δ 9.7 (CH_3_, COCH_2_CH_3_), 26.6 (CH_2_, COCH_2_CH_3_), 32.3 (CH_3_, C9–CH_3_), 32.4 (CH_2_, C3′ or C5′), 33.6 (C, C9), 33.9 (CH_2_, C5′ or C3′), 39.9 [CH_2_, C6(12)], 40.9 (CH_2_, C6′ or C2′), 41.1 [CH, C5(11)], 41.2 [CH_2_, C10(13)], 44.5 (CH_2_, C2′ or C6′), 46.7 (CH, C4′), 48.0 (CH_2_, C8), 53.4 (C, C7), 126.2 [CH, C2(3)], 127.9 [CH, C1(4)], 146.3 [C, C4a(11a)], 156.5 (C, NHCONH), 172.4 (NCOCH_2_CH_3_). Anal. Calcd for C_25_H_35_N_3_O_2_·0.25 H_2_O: C, 72.52; H, 8.64; N, 10.15. Found: C, 72.65; H, 8.49; N, 9.82. HRMS calcd for [C_25_H_35_N_3_O_2_ + H]^+^, 410.2802; found, 410.2801.

#### 4.1.2 1-(9-Methyl-5,6,8,9,10,11-hexahydro-7*H*-5,9:7,11-dimethanobenzo[9]annulen-7-yl)-3-(1-(tetrahydro-2*H*-pyran-4-carbonyl)piperidin-4-yl)urea (**10**)

To a solution of 9-methyl-5,6,8,9,10,11-hexahydro-7*H*-5,9:7,11-dimethanobenzo[9]annulen-7-amine hydrochloride (258 mg, 0.98 mmol) in DCM (4 mL), saturated aqueous NaHCO_3_ solution (4 mL) and triphosgene (107 mg, 0.36 mmol) were added. The biphasic mixture was stirred at room temperature for 30 min, then the two phases were separated, and the organic layer was washed with brine (2 mL), dried over anhyd Na_2_SO_4_, filtered, and evaporated under vacuum to obtain 1–2 mL of a solution of the isocyanate in DCM. To this solution was added (4-aminopiperidin-1-yl)(tetrahydro-2*H*-pyran-4-yl)methanone (215 mg, 1.01 mmol). The reaction mixture was stirred at room temperature overnight, and the solvent was evaporated under vacuum to obtain a yellow residue (534 mg). Column chromatography (SiO_2_, DCM/methanol mixtures) gave urea **10** (207 mg, 45% yield) as a white solid, mp 224–225 °C. IR (NaCl disk): 3357, 3064, 3017, 2945, 2919, 2850, 1640, 1614, 1553, 1493, 1446, 1361, 1344, 1320, 1278, 1261, 1238, 1211, 1126, 1089, 1068, 1018, 984, 941, 874, 818, 759, 733 cm^–1^. ^1^H NMR (400 MHz, CDCl_3_): δ 0.90 (s, 3H, C9–CH_3_), 1.17 [m, 2H, 3′(5′)-H_ax_], 1.50–1.65 [complex signal, 6H, 3″(5″)-H_ax_, 10(13)-H_2_], 1.79 (s, 2H, 8-H), 1.82–1.90 [complex signal, 3H, 5′-H_eq_ or 3′-H_eq_, 3″(5″)-H_eq_], 1.94 [d, *J* = 12.8 Hz, 2H, 6(12)-H_ax_], 2.03–2.16 [complex signal, 3H, 6(12)-H_eq_, 3′-H_eq_ or 5′-H_eq_], 2.65–2.79 (complex signal, 2H, 2′-H_ax_ or 6′-H_ax_, 4″-H), 3.00–3.17 [complex signal, 3H, 6′-H_ax_ or 2′-H_ax_, 5(11)-H], 3.43 [m, 2H, 2″(6″)-H_ax_], 3.69–3.88 (complex signal, 2H, 4′-H, 2′-H_eq_ or 6′-H_eq_), 3.99 [m, 2H, 2″(6″)-H_eq_], 4.48 (m, 2H, 2′-H_eq_ or 6′-H_eq_), 7.02 [m, 2H, 1(4)-H], 7.06 [m, 2H, 2(3)-H]. ^13^C NMR (100.6 MHz, CDCl_3_): δ 29.1 [CH_2_, C3″(5″)], 32.3 (CH_3_, C9–CH_3_), 32.4 (CH_2_, C5′ or C3′), 33.7 (C, C9), 34.1 (CH_2_, C3′ or C5′), 37.6 (CH, C4″), 39.9 [CH_2_, C6(12)], 41.1 [CH, C5(11)], 41.2 [CH_2_, C10(13)], 44.3 [CH_2_, C2′(6′)], 47.0 (CH, C4′), 48.0 (CH_2_, C8), 53.5 (C, C7), 67.1 [CH_2_, C2″(6″)], 126.2 [CH, C2(3)], 127.9 [CH, C1(4)], 146.3 [C, C4a(11a)], 156.3 (C, NHCONH), 172.8 (C, NCOR). Anal. Calcd for C_28_H_39_N_3_O_3_: C, 72.23; H, 8.44; N, 9.02. Found: C, 72.33; H, 8.40; N, 8.83. HRMS calcd for [C_28_H_39_N_3_O_3_ + H]^+^, 466.3064; found, 466.3065.

#### 4.1.3 1-[1-(Isopropylsulfonyl)piperidin-4-yl]-3-(9-methyl-5,6,8,9,10,11-hexahydro-7*H*-5,9:7,11-dimethanobenzo[9]annulen-7-yl)urea (**11**)

To a solution of 9-methyl-5,6,8,9,10,11-hexahydro-7*H*-5,9:7,11-dimethanobenzo[9]annulen-7-amine hydrochloride (300 mg, 1.14 mmol) in DCM (6 mL) and saturated aqueous NaHCO_3_ solution (4 mL) was added triphosgene (169 mg, 0.57 mmol). The biphasic mixture was stirred at room temperature for 30 min, then the two phases were separated, and the organic layer was washed with brine (5 mL), dried over anhyd Na_2_SO_4_, filtered, and evaporated under vacuum to obtain 1–2 mL of a solution of the isocyanate in DCM.

To a solution of 1-(isopropylsulfonyl)piperidin-4-amine (233 mg, 1.13 mmol) in anhyd THF (5 mL) under an argon atmosphere at −78 °C was added dropwise a solution of *n*-butyllithium (2.5 M in hexanes, 0.59 mL, 1.47 mmol) during 20 min. After the addition, the mixture was tempered to 0 °C using an ice bath. This solution was added carefully to the solution of the isocyanate from the previous step cooled to 0 °C, under an argon atmosphere. The reaction mixture was stirred at room temperature overnight. Methanol (2 mL) was then added to quench any unreacted *n*-butyllithium. The solvents were evaporated under vacuum to give an orange gum (506 mg). This residue was dissolved in EtOAc (10 mL) and washed with 2 N HCl solution (2 × 5 mL) and the organic layer was dried over anhyd Na_2_SO_4_, filtered, and concentrated in vacuum to obtain a white gum (241 mg). Column chromatography (SiO_2_, DCM/methanol mixtures) gave a white solid. Crystallization from hot DCM/pentane provided urea **11** (66 mg, 13% yield) as a white solid, mp 218–219 °C. IR (NaCl disk): 3364, 3062, 3013, 2946, 2920, 2854, 1710, 1638, 1553, 1494, 1453, 1361, 1320, 1305, 1266, 1249, 1232, 1168, 1134, 1091, 1045, 943, 881, 841, 759, 732, 666, 593, 555 cm^–1^. ^1^H NMR (400 MHz, CDCl_3_): δ 0.90 (s, 3H, C9–CH_3_), 1.31 [d, *J* = 6.8 Hz, 6H, CH(CH_3_)_2_], 1.36 [dq, *J* = 12.0 Hz, *J*′ = 4.0 Hz, 3′(5′)-H_ax_], 1.52 [d, *J* = 13.2 Hz, 2H, 10(13)-H_ax_], 1.61 [m, 2H, 10(13)-H_eq_], 1.79 (s, 2H, 8-H), 1.92–1.97 [complex signal, 4H, 3′(5′)-H_eq_, 6(12)-H_ax_], 2.12 [dd, *J* = 12.8 Hz, *J*′ = 6.4 Hz, 2H, 6(12)-H_eq_], 2.92 [m, 2H, 2′(6′)-H_ax_], 3.04 [t, *J* = 6.4 Hz, 2H, 5(11)-H], 3.15 (sept, *J* = 6.8 Hz, 1H, CH(CH_3_)_2_), 3.67 (m, 1H, 4′-H), 3.78 [dm, *J* = 13.2 Hz, 2H, 2′(6′)-H_eq_], 4.35 (s, 1H, C7–NH), 4.41 (d, *J* = 8.0 Hz, C4′-NH), 7.03 [m, 2H, 1(4)-H], 7.06 [m, 2H, 2(3)-H]. ^13^C NMR (100.6 MHz, CDCl_3_): δ 16.7 [CH_3_, CH(CH_3_)_2_], 32.3 (CH_3_, C9–CH_3_), 33.4 [CH_2_, C3′(5′)], 33.7 (C, C9), 39.8 [CH_2_, C6(12)], 41.1 [CH, C5(11)], 41.2 [CH_2_, C10(13)], 45.7 [CH_2_, C2′(6′)], 46.6 (CH, C4′), 48.0 (CH_2_, C8), 53.4 [CH, CH(CH_3_)_2_], 53.5 (C, C7), 126.2 [CH, C2(3)], 127.9 [CH, C1(4)], 146.3 [C, C4a(11a)], 156.2 (C, NHCONH). Anal. Calcd for C_25_H_37_N_3_O_3_S: C, 65.33; H, 8.11; N, 9.14. Found: C, 65.41; H, 8.31; N, 8.93. HRMS calcd for [C_25_H_37_N_3_O_3_S + H]^+^, 460.2628; found, 460.2623.

#### 4.1.4 1-(9-Methyl-5,6,8,9,10,11-hexahydro-7*H*-5,9:7,11-dimethanobenzo[9]annulen-7-yl)-3-(1-(cyclopropanecarbonyl)piperidin-4-yl)urea (**12**)

To a solution of 9-methyl-5,6,8,9,10,11-hexahydro-7*H*-5,9:7,11-dimethanobenzo[9]annulen-7-amine hydrochloride (112.5 mg, 0.43 mmol) in DCM (6 mL), saturated aqueous NaHCO_3_ solution (5 mL) and triphosgene (93.8 mg, 0.16 mmol) were added. The biphasic mixture was stirred at room temperature for 30 min and then the two phases were separated and the organic layer was washed with brine (5 mL), dried over anhyd Na_2_SO_4_, filtered, and evaporated under vacuum to obtain 2–3 mL of a solution of the isocyanate in DCM. To this solution was added (4-aminopiperidin-1-yl)(tetrahydro-2*H*-pyran-4-yl)methanone (72 mg, 0.43 mmol). The mixture was stirred overnight at room temperature, and the solvent was then evaporated. Column chromatography (SiO_2,_ DCM/methanol mixtures) provided urea **12** as a white solid (60 mg, 33% yield), mp 115–120 °C. IR (ATR): 3341, 2899, 1633, 1607, 1549, 1448, 1311, 1222, 1128, 1064, 1027, 979, 756. ^1^H NMR (400 MHz, CDCl_3_): δ 0.72 [dd, 2H, *J* = 6.0 Hz, *J*′ = 2.0 Hz, 8′(9′)-H_ax_], 0.90 (s, 3H, 9′-H), 0.93 [m, 2H, 8′(9′)-H_eq_], 1.20 [m, 2H, 3′(5′)-H_eq_], 1.52 [d, 2H, *J* = 13.2 Hz, 10(13)-H_ax_], 1.63 [dd, 2H, *J* = 13.2 Hz, *J*′ = 5.6 Hz, 10(13)-H_eq_], 1.73 (m, 2H, 7′-H), 1.80 [m, 3H, 8-H, 3′ or 5′-H_eq_], 1.95 [d, 2H, *J* = 12.8 Hz, 6(12)-H_ax_], 2.04 [d, 1H, *J* = 12.0 Hz, 3′ or 5′-H_eq_], 2.13 [dd, 2H, *J* = 12.0 Hz, *J*′ = 6.4 Hz, 6(12)-H_eq_], 2.73 [t, 1H, *J* = 11.6 Hz, 2′ or 6′-H_ax_], 3.04 [t, 2H, *J* = 12.0 Hz, 5(11)-H], 3.20 [t, 1H, *J* = 12 Hz, 2′ or 6′-H_ax_], 3.75 (m, 1H, 4′-H), 4.1 [d, 1H, *J* = 10.8 Hz, 2′ or 6′-H_eq_], 4.4 [d, 1H, *J* = 10 Hz, 2′ or 6′-H_eq_], 4.5 [d, 1H, *J* = 10.8 Hz, NHCONH], 4.6 (s, 1H, NHCONH), 7.03 [m, 2H, 1(4)-H], 7.05 [m, 2H, 2(3)-H]. ^13^C NMR (100.5 MHz, CDCl_3_): δ 7.49 [CH_2_, C8′(9′)], 11.14 (CH, C7′), 32.43 (CH_3_, C9′), 32.53 (CH_2_, C3′ or 5′), 33.82 (CH_2_, C3′ or 5′), 40.03 [CH_2_, C6(12)], 41.26 [CH, C5(11)], 41.35 [CH_2,_ C10(13)], 41.66 [CH_2,_ C2′ or 6′], 44.71 [CH_2,_ C2′or 6′], 47.04 (CH, C4′), 48.13 (CH_2,_ C8), 53.58 (C, C7), 126.33 [CH, C(1)4], 128.07 [CH, C2(3)], 146.49 {C, C4_a_(11_a_)], 156.57 (C, NHCONH), 172.22 (C, NCOR). Anal. Calcd for C_26_H_35_N_3_O_2_·0.1 CH_2_Cl_2_: C, 72.89; H, 8.25; N, 9.77. Found: C, 73.08; H, 8.23; N, 9.53. HRMS calcd for [C_26_H_35_N_3_O_2_ + H]^+^, 422.2802; found, 422.2808.

#### 4.1.5 1-(1-Acetylpiperidin-4-yl)-3-(9-chloro-5,6,8,9,10,11-hexahydro-7*H*-5,9:7,11-dimethanobenzo[9]annulen-7-yl)urea (**13**)

To a solution of 9-chloro-5,6,8,9,10,11-hexahydro-7*H*-5,9:7,11-dimethanobenzo[9]annulen-7-amine hydrochloride (150 mg, 0.53 mmol) in DCM (3 mL), saturated aqueous NaHCO_3_ solution (3 mL) and triphosgene (58 mg, 0.20 mmol) were added. The biphasic mixture was stirred at room temperature for 30 min, then the two phases were separated, and the organic layer was washed with brine (3 mL), dried over anhyd Na_2_SO_4_, filtered, and evaporated under vacuum to obtain 1–2 mL of a solution of the isocyanate in DCM. To this solution was added 1-(4-aminopiperidin-1-yl)ethan-1-one (90 mg, 0.63 mmol). The reaction mixture was stirred at room temperature overnight, and the solvent was evaporated under vacuum to obtain a white solid (204 mg). Column chromatography (SiO_2_, DCM/methanol mixtures) gave urea **13** (115 mg, 52% yield) as a white solid, mp 209–210 °C. IR (NaCl disk): 3358, 3019, 2926, 2855, 1644, 1619, 1556, 1494, 1452, 1358, 1319, 1301, 1268, 1228, 1206, 1135, 1090, 1050, 991, 969, 947, 802, 761, 735 cm^–1^. ^1^H NMR (400 MHz, CDCl_3_): δ 1.15 (m, 1H, 5′-H_ax_ or 3′-H_ax_), 1.18 [m, 1H, 3′-H_ax_ or 5′-H_ax_], 1.85 (d, *J* = 13.6 Hz, 1H, 5′-H_eq_ or 3′-H_eq_), 1.93 [d, *J* = 13.2 Hz, 2H, 6(12)-H_ax_], 2.02 (m, 1H, 3′-H_eq_ or 5′-H_eq_), 2.06 (s, 3H, COCH_3_), 2.15 [d, *J* = 13.6 Hz, 2H, 10(13)-H_ax_], 2.21 [m, 2H, 6(12)-H_eq_], 2.35 [dd, *J* = 12.8 Hz, *J*′ = 6.0 Hz, 2H, 10(13)-H_eq_], 2.45 (m, 1H, 8-H_a_), 2.48 (m, 1H, 8-H_b_), 2.72 (m, 1H, 2′-H_ax_ or 6′-H_ax_), 3.05–3.19 (complex signal, 3H, 5(11)-H, 6′-H_ax_ or 2′-H_ax_), 3.67–3.80 (complex signal, 2H, 4′-H, 6′-H_eq_ or 2′-H_eq_), 4.41 (dm, *J* = 13.6 Hz, 1H, 2′-H_eq_ or 6′-H_eq_), 4.78 (d, *J* = 7.6 Hz, 1H, C4′-NH), 4.85 (s, 1H, C7–NH), 7.04 [m, 2H, 1(4)-H], 7.09 [m, 2H, 2(3)-H]. ^13^C NMR (100.6 MHz, CDCl_3_): δ 21.5 (CH_3_, COCH_3_), 32.4 (CH_2_, C5′ or C3′), 33.7 (CH_2_, C3′ or C5′), 38.89 (CH_2_, C6 or C12), 38.96 (CH_2_, C12 or C6), 40.7 (CH_2_, C2′ or C6′), 41.2 [CH, C5(11)], 44.47 (CH_2_, C10 or C13), 44.50 (CH_2_, C13 or C10), 45.4 (CH_2_, C6′ or C2′), 46.6 (CH, C4′), 50.8 (CH_2_, C8), 55.5 (C, C7), 69.5 (C, C9), 126.8 [CH, C2(3)], 128.1 [CH, C1(4)], 144.7 (CH, C4a or C11a), 144.8 (CH, C11a or C4a), 156.2 (C, NHCONH), 169.17 (C, COCH_3_). Anal. Calcd for C_23_H_30_ClN_3_O_2_·0.75 ethyl acetate: C, 64.78; H, 7.53; N, 8.72. Found: C, 64.73; H, 7.56; N, 8.89. HRMS calcd for [C_23_H_30_ClN_3_O_2_ + H]^+^, 416.2099; found, 416.2100.

#### 4.1.6 1-(9-Chloro-5,6,8,9,10,11-hexahydro-7*H*-5,9:7,11-dimethanobenzo[9]annulen-7-yl)-3-(1-propionylpiperidin-4-yl)urea (**14**)

To a solution of 9-chloro-5,6,8,9,10,11-hexahydro-7*H*-5,9:7,11-dimethanobenzo[9]annulen-7-amine hydrochloride (150 mg, 0.53 mmol) in DCM (4 mL) and saturated aqueous NaHCO_3_ solution (3 mL), triphosgene (56 mg, 0.19 mmol) was added. The biphasic mixture was stirred at room temperature for 30 min, then the two phases were separated, and the organic one was washed with brine (3 mL), dried over anhyd Na_2_SO_4_, filtered, and evaporated under vacuum to obtain 1–2 mL of a solution of isocyanate in DCM. To this solution was added 1-(4-aminopiperidin-1-yl)propan-1-one (83 mg, 0.53 mmol). The mixture was stirred overnight at room temperature, and the solvent was then evaporated. Column chromatography (SiO_2,_ DCM/methanol mixtures) provided an orange solid. The analytical sample was obtained by a crystallization from hot ethyl acetate/pentane mixtures to obtain a urea **14** as a yellowish solid (79 mg, 35% yield), mp 155–156 °C. IR (ATR): 3359, 2924, 2852, 1681, 1652, 1637, 1612, 1565, 1447, 1373, 1356, 1322, 1297, 1263, 1221, 1134, 1075, 1045, 1022, 967, 946, 908, 804, 755, 618, 559 cm^–1^. ^1^H NMR (400 MHz, CDCl_3_): δ 1.11 (t, *J* = 7.2 Hz, 3H, COCH_2_CH_3_), 1.13 [m, 2H, 5′(3′)-H_ax_], 1.84 (d, *J* = 12.8 Hz, 1H, 5′-H_eq_ or 3′-H_eq_), 1.94 [d, *J* = 12.8 Hz, 2H, 6(12)-H_ax_], 2.00 (d, *J* = 12.4 Hz, 3′-H_eq_ or 5′-H_eq_), 2.14 [d, *J* = 13.2 Hz, 2H, 10(13)-H_ax_], 2.21 [m, 2H, 6(12)-H_eq_], 2.29–2.40 [complex signal, 4H, 10(13)-H_eq_, COCH_2_CH_3_], 2.48 (m, 2H, 8-H), 2.70 (m, 1H, 2′-H_ax_ or 6′-H_ax_), 3.08 (m, 1H, 6′-H_ax_ or 2′-H_ax_), 3.14 [t, *J* = 6.4 Hz, 2H, 5(11)-H], 3.68–3.82 (complex signal, 2H, 6′-H_eq_ or 2′-H_eq_, 4′-H), 4.45 (dm, *J* = 13.6 Hz, 1H, 2′-H_eq_ or 6′-H_eq_), 4.68 (d, *J* = 8.0 Hz, 1H, C4′-NH), 4.75 (s, 1H, C7–NH), 7.05 [m, 2H, 1(4)-H], 7.10 [m, 2H, 2(3)-H]. ^13^C NMR (100.6 MHz, CDCl_3_): δ 9.7 (CH_3_, COCH_2_CH_3_), 26.6 (CH_2_, COCH_2_CH_3_), 32.4 (CH_2_, C5′ or C3′), 33.9 (CH_2_, C3′ or C5′), 38.9 [CH_2_, C6(12)], 40.9 (CH_2_, C2′ or C6′), 41.2 [CH, C5(11)], 44.5 [2 CH_2_, C10(13), C6′ or C2′], 46.7 (CH, C4′), 50.8 (CH_2_, C8), 55.5 (C, C7), 69.5 (C, C9), 126.8 [CH, C2(3)], 128.1 [CH, C1(4)], 144.8 [C4a(11a)], 156.1 (NHCONH), 172.5 (NCOR). HRMS calcd for [C_24_H_32_ClN_3_O_2_ + H]^+^, 430.2256; found, 430.2253. Anal. Calcd for C_24_H_32_ClN_3_O_2_·0.75 H_2_O: C, 65.00; H, 7.61; N, 9.47. Found: C, 65.27; H, 7.51; N, 9.15.

#### 4.1.7 1-(9-Chloro-5,6,8,9,10,11-hexahydro-7*H*-5,9:7,11-dimethanobenzo[9]annulen-7-yl)-3-(1-(tetrahydro-2*H*-pyran-4-carbonyl)piperidin-4-yl)urea (**15**)

To a solution of 9-chloro-5,6,8,9,10,11-hexahydro-7*H*-5,9:7,11-dimethanobenzo[9]annulen-7-amine hydrochloride (130 mg, 0.46 mmol) in DCM (4 mL) and saturated aqueous NaHCO_3_ solution (3 mL), triphosgene (50 mg, 0.17 mmol) was added. The biphasic mixture was stirred at room temperature for 30 min, then the two phases were separated, and the organic one was washed with brine (3 mL), dried over anhyd Na_2_SO_4_, filtered, and evaporated under vacuum to obtain 1–2 mL of a solution of isocyanate in DCM. To this solution was added (4-aminopiperidin-1-yl) (tetrahydro-2*H*-pyran-4-yl)methanone (97 mg, 0.46 mmol). The mixture was stirred overnight at room temperature, and the solvent was then evaporated. Column chromatography (SiO_2,_ DCM/methanol mixtures) provided urea **15** as a yellowish solid (90 mg, 41% yield). The analytical sample was obtained by washing the product with ethyl acetate to obtain a white solid, mp 214–215 °C. IR (ATR): 2924, 2851, 1675, 1610, 1546, 1493, 1451, 1361, 1319, 1296, 1282, 1246, 1225, 1208, 1120, 1084, 1017, 991, 946, 908, 874, 810, 755, 730, 696, 644, 619, 564 cm^–1^. ^1^H NMR (400 MHz, CDCl_3_): δ 1.18 [dq, *J* = 12.0 Hz, *J*′ = 4.0 Hz, 2H, 3′(5′)-H_ax_], 1.56 [m, 2H, 3″(5″)-H_ax_], 1.80–1.91 [complex signal, 3H, 3″(5″)-H_eq_, 3′-H_eq_ or 5′-H_eq_], 1.94 [d, *J* = 13.2 Hz, 2H, 6(12)-H_ax_], 2.08 (d, *J* = 12.8 Hz, 1H, 5′-H_eq_ or 3′-H_eq_), 2.16 [d, 2H, 10(13)-H_ax_], 2.20 [m, 2H, 6(12)-H_eq_] 2.36 [dd, *J* = 13.2 Hz, *J*′= 6.4 Hz, 2H, 10(13)-H_eq_], 2.48 (s, 2H, 8-H), 2.66–2.78 (complex signal, 2H, 4″-H, 6′-H_ax_ or 2′-H_ax_), 3.11 (m, 1H, 2′-H_ax_ or 6′-H_ax_), 3.15 [t, *J* = 6.0 Hz, 2H, 5(11)-H], 3.43 [t, *J* = 11.6 Hz, 2H, 2″(6″)-H_ax_], 3.75 (m, 1H, 4′-H), 3.83 (d, *J* = 13.2 Hz, 1H, 2′-H_eq_ or 6′-H_eq_), 3.99 [dm, *J* = 11.6 Hz, 2″(6″)-H_eq_], 4.46 (m, 1H, 6′-H_eq_ or 2′-H_eq_), 4.51 (d, *J* = 7.6 Hz, 1H, C4′-NH), 4.57 (s, 1H, C7–NH), 7.06 [m, 2H, 1(4)-H], 7.09 [m, 2H, 2(3)-H]. ^13^C NMR (100.6 MHz, CDCl_3_): δ 29.1 [CH_2_, C3″(5″)], 32.4 (CH_2_, C5′ or C3′), 34.2 (CH_2_, C3′ or C5′), 37.6 (CH, C4″), 38.9 [CH_2_, C6(12)], 41.1 (CH_2_, C6′ or C2′), 41.2 [CH, C5(11)], 44.3 (CH_2_, C2′ or C6′), 44.5 [CH_2_, C10(13)], 47.0 (CH, C4′), 50.8 (CH_2_, C8), 55.6 (C, C7), 67.2 [CH_2_, C2″(6″)], 69.5 (C, C9), 126.8 [CH, 2(3)], 128.1 [CH, 1(4)], 144.7 [C, C5a(11a)], 156.0 (NHCONH), 172.9 (NCOR). HRMS calcd for [C_27_H_36_ClN_3_O_3_ + H]^+^, 486.2518; found, 486.2522. Anal. Calcd for C_27_H_36_ClN_3_O_3_: C, 66.72; H, 7.47; N, 8.65. Found: C, 66.92; H, 7.40; N, 8.43. HPLC: *t*_r_ = 4.523 (λ = 220 nm, 97.1% purity).

#### 4.1.8 1-(9-Chloro-5,6,8,9,10,11-hexahydro-7*H*-5,9:7,11-dimethanobenzo[9]annulen-7-yl)-3-(1-(isopropylsulfonyl)piperidin-4-yl)urea (**16**)

To a solution of 9-chloro-5,6,8,9,10,11-hexahydro-7*H*-5,9:7,11-dimethanobenzo[9]annulen-7-amine hydrochloride (268 mg, 0.94 mmol) in DCM (8 mL) and saturated aqueous NaHCO_3_ solution (5 mL) was added triphosgene (103 mg, 0.35 mmol). The biphasic mixture was stirred at room temperature for 30 min, then the two phases were separated, and the organic layer was washed with brine (5 mL), dried over anhyd Na_2_SO_4_, filtered, and evaporated under vacuum to obtain 1–2 mL of a solution of the isocyanate in DCM.

To a solution of 1-(isopropylsulfonyl)piperidin-4-amine (194 mg, 0.94 mmol) in anhyd THF (8 mL) under an argon atmosphere at −78 °C was added dropwise a solution of *n*-butyllithium (2.5 M in hexanes, 0.49 mL, 1.22 mmol) for 20 min. After the addition, the mixture was tempered to 0 °C using an ice bath. This solution was added carefully to the solution of the isocyanate from the previous step cooled to 0 °C, under an argon atmosphere. The reaction mixture was stirred at room temperature overnight. Methanol (2 mL) was then added to quench any unreacted *n*-butyllithium. The solvents were evaporated under vacuum to give a yellow residue (690 mg). Column chromatography (SiO_2_, DCM/methanol mixtures) gave a white solid. Crystallization from hot DCM/pentane provided urea **16** as a yellowish solid (75 mg, 17% yield). The analytical sample was obtained by crystallization from hot ethyl acetate/pentane mixtures, mp 223–224 °C. IR (NaCl disk): 3407, 3370, 2926, 2856, 1672, 1538, 1494, 1451, 1353, 1304, 1296, 1223, 1209, 1177, 1130, 1090, 1045, 972, 949, 903, 885, 841, 805, 767, 735, 668, 623 cm^–1^. ^1^H NMR (400 MHz, CDCl_3_): δ 1.31 [d, *J* = 6.8 Hz, 6H, CH(CH_3_)_2_], 1.37 [dq, *J* = 12.4 Hz, *J*′ = 4.0 Hz, 2H, 3′(5′)-H_ax_], 1.91–1.99 [complex signal, 4H, 6(12)-H_ax_, 3′(5′)-H_eq_], 2.15 [d, *J* = 13.2 Hz, 2H, 10(13)-H_ax_], 2.20 [dd, *J* = 13.6 Hz, *J*′ = 5.6 Hz, 6(12)-H_eq_], 2.35 [dd, *J* = 13.6 Hz, *J*′ = 5.6 Hz, 10(13)-H_eq_], 2.47 (s, 2H, 8-H), 2.93 [dt, *J* = 13.2 Hz, *J*′ = 2.6 Hz, 2H, 2′(6′)-H_ax_], 3.11–3.22 [complex signal, 3H, CH(CH_3_)_2_, 5(11)-H], 3.67 (m, 1H, 4′-H), 3.79 [dm, *J* = 13.2 Hz, 2H, 2′(6′)-H_eq_], 4.50 (s, 1H, C7–NH), 4.54 (d, *J* = 7.6 Hz, C4′-NH), 7.04 [m, 2H, 1(4)-H], 7.10 [m, 2H, 2(3)-H]. ^13^C NMR (100.6 MHz, CDCl_3_): δ 16.7 [CH_3_, CH(CH_3_)_2_], 33.4 [CH_2_, C3′(5′)], 38.9 [CH_2_, C6(12)], 41.2 [CH, C5(11)], 44.5 [CH_2_, C10(13)], 45.7 [CH_2_, C2′(6′)], 46.6 (CH, C4′), 50.8 (CH_2_, C8), 53.4 [CH, CH(CH_3_)_2_], 55.6 (C, C7), 69.5 (C, C9), 126.8 [CH, C2(3)], 128.1 [CH, C1(4)], 144.8 [C, C4a(11a)], 156.0 (C, NHCONH). HRMS calcd for [C_24_H_34_ClN_3_O_3_S + H]^+^, 480.2082; found, 480.2084. Anal. Calcd for C_24_H_34_ClN_3_O_3_S·0.05 ethyl acetate: C, 60.00; H, 7.16; N, 8.67. Found: C, 60.38; H, 7.08; N, 8.27.

#### 4.1.9 1-(9-Chloro-5,6,8,9,10,11-hexahydro-7*H*-5,9:7,11-dimethanobenzo[9]annulen-7-yl)-3-(1-(cyclopropanecarbonyl)piperidin-4-yl)urea (**17**)

To a solution of 9-chloro-5,6,8,9,10,11-hexahydro-7*H*-5,9:7,11-dimethanobenzo[9]annulen-7-amine hydrochloride (130 mg, 0.46 mmol) in DCM (4 mL) and saturated aqueous NaHCO_3_ solution (3 mL), triphosgene (50 mg, 0.17 mmol) was added. The biphasic mixture was stirred at room temperature for 30 min, then the two phases were separated, and the organic one was washed with brine (3 mL), dried over anhYd Na_2_SO_4_, filtered, and evaporated under vacuum to obtain 1–2 mL of a solution of isocyanate in DCM. To this solution was added (4-aminopiperidin-1-yl) (cyclopropyl)methanone (77 mg, 0.46 mmol). The mixture was stirred overnight at room temperature, and the solvent was then evaporated. Column chromatography (SiO_2,_ DCM/methanol mixtures) provided urea **17** as a white solid (70 mg, 35% yield), mp 119–120 °C. IR (ATR): 3367, 3330, 2926, 2853, 1682, 1654, 1605, 1565, 1550, 1481, 1452, 1374, 1357, 1319, 1299, 1264, 1224, 1128, 1088, 1036, 1013, 993, 967, 948, 925, 911, 870, 799, 755, 735, 700, 632, 604, 564 cm^–1^. ^1^H NMR (400 MHz, CDCl_3_): δ 0.75 (m, 2H, 2″(3″)-H_ax_), 0.94 [m, 2H, 2″(3″)-H_eq_], 1.23 [m, 2H, 3′(5′)-H_ax_], 1.74 (m, 1H, 1″-H), 1.88 [m, 1H, 5′-H_eq_ or 3′-H_eq_], 1.95 [d, *J* = 13.2 Hz, 2H, 6(12)-H_ax_], 2.08 (m, 1H, 3′-H_eq_ or 5′-H_eq_), 2.16 [d, *J* = 13.2 Hz, 2H, 10(13)-H_ax_], 2.23 [m, 2H, 6(12)-H_eq_], 2.37 [dd, *J* = 12.0 Hz, *J*′ = 6.4 Hz, 2H, 10(13)-H_ax_], 2.50 (s, 2H, 8-H), 2.73 (broad t, *J* = 12.0 Hz, 1H, 2′-H_ax_ or 6′-H_ax_), 3.16 [t, *J* = 6.4 Hz, 2H, 5(11)-H], 3.21 (m, 1H, 6′-H_ax_ or 2′-H_ax_), 3.77 (m, 1H, 4′-H), 4.14 (m, 1H, 6′-H_eq_ or 2′-H_eq_), 4.23 (d, *J* = 8.0 Hz, 1H, C4′-NH), 4.30 (s, 1H, C7–NH), 4.48 (dm, *J* = 12.0 Hz, 1H, 3′-H_eq_ or 5′-H_eq_), 7.05 [m, 2H, 1(4)-H], 7.11 [m, 2H, 2(3)-H]. ^13^C NMR (100.6 MHz, CDCl_3_): δ 7.4 [CH_2_, C2″(3″)], 11.0 (CH, C1″), 32.3 (CH_2_, C5′ or C3′), 34.1 (CH_2_, C3′ or C5′), 38.9 [CH_2_, C6(12)], 41.3 [CH, C5(11)], 41.6 (CH_2_, C2′ or C6′), 44.5 [CH_2_, C10(13)], 44.6 (CH_2_, C6′ or C2′), 46.7 (CH, C4′), 50.8 (CH_2_, C8), 55.5 (C, C7), 69.6 (C, C9), 126.8 [CH, C2(3)], 128.1 [CH, C1(4)], 144.8 (C, C4a(11a)], 156.3 (C, NHCONH), 172.2 (C, NCOR). HRMS calcd for [C_25_H_32_ClN_3_O_2_ + H]^+^, 442.2256; found, 442.2262. Anal. Calcd for C_25_H_32_ClN_3_O_2_·0.75 H_2_O: C, 66.05; H, 7.41; N, 9.24. Found: C, 66.21; H, 7.31; N 9.00.

#### 4.1.10 1-(9-Chloro-5,6,8,9,10,11-hexahydro-7*H*-5,9:7,11-dimethanobenzo[9]annulen-7-yl)-3-(1-(2,2,2-trifluoroacetyl)piperidin-4-yl)urea (**18**)

To a solution of 9-chloro-5,6,8,9,10,11-hexahydro-7*H*-5,9:7,11-dimethanobenzo[9]annulen-7-amine hydrochloride (130 mg, 0.46 mmol) in DCM (4 mL) and saturated aqueous NaHCO_3_ solution (3 mL), triphosgene (50 mg, 0.17 mmol) was added. The biphasic mixture was stirred at room temperature for 30 min, then the two phases were separated, and the organic one was washed with brine (3 mL), dried over anhyd Na_2_SO_4_, filtered, and evaporated under vacuum to obtain 1–2 mL of a solution of isocyanate in DCM. To this solution was added 1-(4-aminopiperidin-1-yl)-2,2,2-trifluoroethan-1-one hydrochloride (106 mg, 0.46 mmol) and Et_3_N (92 mg, 0.91 mmol). The mixture was stirred overnight at room temperature, and the mixture was washed with water (15 mL). The organic phase was dried over anhyd Na_2_SO_4_, filtered, and evaporated under vacuum to obtain an orange gum (196 mg). Column chromatography (SiO_2,_ DCM/methanol mixtures) provided urea **18** as a yellowish solid (55 mg, 26% yield). The analytical sample was obtained by a crystallization from hot ethyl acetate/pentane mixtures, mp 188–189 °C. IR (ATR): 3348, 2926, 2859, 1689, 1634, 1556, 1495, 1466, 1454, 1357, 1298, 1266, 1203, 1179, 1137, 1091, 1044, 1009, 992, 971, 946, 897, 802, 757, 698, 660, 623, 599, 556 cm^–1^. ^1^H NMR (400 MHz, CDCl_3_): δ 1.30 [m, 2H, 5′(3′)-H_ax_], 1.94 [d, *J* = 12.8 Hz, 2H, 6(12)-H_ax_], 2.03 [m, 2H, 5′(3′)-H_eq_], 2.16 [d, *J* = 13.6 Hz, 2H, 10(13)-H_ax_], 2.20 [m, 2H, 6(12)-H_eq_], 2.36 [dd, *J* = 13.6 Hz, *J*′ = 13.6 Hz, 2H, 10(13)-H_eq_], 2.47 (s, 2H, 8-H), 2.89 (t, *J* = 12.0 Hz, 1H, 2′-H_ax_ or 6′-H_ax_), 3.13–3.25 [complex signal, 3H, 5(11)-H, 6′-H_ax_ or 2′-H_ax_], 3.80 (m, 1H, 4′-H), 3.95 (d, *J* = 14.0 Hz, 1H, 6′-H_eq_ or 2′-H_eq_), 4.28 (d, *J* = 7.6 Hz, 1H, C4′-NH), 4.32 (s, 1H, C7–NH), 4.42 (dm, *J* = 14.0 Hz, 2′-H_eq_ or 6′-H_eq_), 7.05 [m, 2H, 1(4)-H], 7.09 [m, 2H, 2(3)-H]. ^13^C NMR (100.6 MHz, CDCl_3_): δ 32.2 (CH_2_, C5′ or C3′), 33.3 (CH_2_, C3′ or C5′), 38.91 (CH_2_, C6 or C12), 38.92 (CH_2_, C12 or C6), 41.2 [CH, C5(11)], 42.8 (CH_2_, C2′ or C6′), 44.4 [CH_2_, C10(13)], 44.7 (q, ^*4*^*J*_C–F_ = 3.5 Hz, CH_2_, C6′ or C2′), 46.7 (CH, C4′), 50.8 (CH_2_, C8), 55.8 (C, C7), 69.3 (C, C9), 116.5 (q, ^1^*J*_C–F_ = 287.7 Hz, C, CF_3_), 126.8 [CH, C2(3)], 128.1 [CH, C1(4)], 144.7 [C, C4a(11a)], 155.3 (C, NCOR), 155.6 (C, NHCONH). HRMS calcd for [C_23_H_27_ClF_3_N_3_O_2_ – H]^−^, 468.1671; found, 468.1671. Anal. Calcd for C_23_H_27_ClF_3_N_3_O_2_·0.75 CH_3_OH: C, 57.75; H, 6.12; N, 8.51. Found: C, 58.04; H, 5.82; N, 8.20.

#### 4.1.11 1-(9-Chloro-5,6,8,9,10,11-hexahydro-7*H*-5,9:7,11-dimethanobenzo[9]annulen-7-yl)-3-(1-(1-fluorocyclopropane-1-carbonyl)piperidin-4-yl)urea (**19**)

To a solution of 9-chloro-5,6,8,9,10,11-hexahydro-7*H*-5,9:7,11-dimethanobenzo[9]annulen-7-amine hydrochloride (130 mg, 0.46 mmol) in DCM (4 mL) and saturated aqueous NaHCO_3_ solution (3 mL), triphosgene (50 mg, 0.17 mmol) was added. The biphasic mixture was stirred at room temperature for 30 min, then the two phases were separated, and the organic one was washed with brine (3 mL), dried over anhyd Na_2_SO_4_, filtered, and evaporated under vacuum to obtain 1–2 mL of a solution of isocyanate in DCM. To this solution were added (4-aminopiperidin-1-yl) (1-fluorocyclopropyl)methanone hydrochloride (101 mg, 0.46 mmol) and Et_3_N (92 mg, 0.91 mmol). The mixture was stirred overnight at room temperature, and the mixture was washed with water (10 mL). The organic phase was dried over anhyd Na_2_SO_4_, filtered, and evaporated under vacuum to obtain an orange gum (140 mg). Column chromatography (SiO_2,_ DCM/methanol mixtures) provided urea **19** as a yellowish solid (20 mg, 10% yield). The analytical sample was obtained by a crystallization from hot ethyl acetate/pentane mixtures, mp 120–121 °C. IR (ATR): 3340, 2921, 2856, 1730, 1632, 1553, 1493, 1453, 1439, 1356, 1327, 1299, 1274, 1244, 1204, 1122, 1088, 1047, 1025, 993, 970, 947, 907, 801, 760, 729, 697, 680, 643 cm^–1^. ^1^H NMR (400 MHz, CDCl_3_): δ 1.14–1.38 [complex signal, 6H, 2″(3″)-H_2_, 5′(3′)-H_ax_], 1.95 [d, *J* = 13.2 Hz, 2H, 6(12)-H_ax_], 2.00 [m, 2H, 5′(3′)-H_eq_], 2.16 [d, *J* = 13.2 Hz, 2H, 10(13)-H_ax_], 2.22 [dd, *J* = 12.4 Hz, *J*′ = 5.6 Hz, 2H, 6(12)-H_eq_], 2.36 [dd, *J* = 12.4 Hz, *J*′ = 6 Hz, 10(13)-H_eq_], 2.49 (s, 2H, 8-H), 2.83 (m, 1H, 2′-H_ax_ or 6′-H_ax_), 3.15 (broad signal, 1H, 6′-H_ax_ or 2′-H_ax_), 3.16 [t, *J* = 6.4 Hz, 2H, 5(11)-H], 3.79 (m, 1H, 4′-H), 4.21 (d, *J* = 8.0 Hz, 1H, C4′-NH), 4.27 (s, 1H, C7–NH), 4.18–4.22 (m, 2H, 2′-H_eq_, 6′-H_eq_), 7.06 [m, 2H, 2(3)-H], 7.10 [m, 2H, 1(4)-H]. ^13^C NMR (100.6 MHz, CDCl_3_): δ 11.8 [CH_2_, 2″(3″)-H], 33.6 [CH_2_, C3′(5′)], 38.9 [CH_2_, C6(12)], 41.2 [CH, C5(11)], 44.5 [2 CH_2_, C10(13), C2′(6′)], 47.2 (CH, C4′), 50.8 (CH_2_, C8), 55.7 (C, C7), 69.4 (C, C9), 79.2 (C, C1″), 126.8 [CH, C2(3)], 128.1 [CH, C1(4)], 144.7 [C, C4a(11a)], 155.7 (C, NHCONH), 166.5 (C, NCOR). HRMS calcd for [C_25_H_31_ClFN_3_O_2_ + H]^+^, 460.2162; found, 460.2165. HPLC: *t*_r_ = 4.297 (λ = 220 nm, 97.2% purity).

#### 4.1.12 1-(1-Acetylpiperidin-4-yl)-3-(9-fluoro-5,6,8,9,10,11-hexahydro-7*H*-5,9:7,11-dimethanobenzo[9]annulen-7-yl)urea (**20**)

To a solution of 9-fluoro-5,6,8,9,10,11-hexahydro-7*H*-5,9:7,11-dimethanobenzo[9]annulen-7-amine hydrochloride (143 mg, 0.53 mmol) in DCM (4 mL) and saturated aqueous NaHCO_3_ solution (2 mL) was added triphosgene (78 mg, 0.26 mmol). The biphasic mixture was stirred at room temperature for 30 min, then the two phases were separated, and the organic layer was washed with brine (5 mL), dried over anhyd Na_2_SO_4_, filtered, and evaporated under vacuum to obtain 1–2 mL of a solution of the isocyanate in DCM. To this solution was added 1-(4-aminopiperidin-1-yl)ethan-1-one (90 mg, 0.63 mmol). The reaction mixture was stirred at room temperature overnight, and the solvent was evaporated under vacuum to obtain a yellow gum (259 mg). Column chromatography (SiO_2_, DCM/methanol mixtures) gave urea **20** (180 mg, 85% yield). The analytical sample was obtained by crystallization from hot DCM (57 mg), mp 228–229 °C. IR (NaCl disk): 3357, 3063, 3018, 2928, 2857, 1684, 1643, 1618, 1553, 1494, 1451, 1359, 1341, 1317, 1267, 1227, 1207, 1135, 1097, 1042, 1004 cm^–1^. ^1^H NMR (400 MHz, CDCl_3_): δ 1.15 [dq, *J* = 12.0 Hz, *J*′ = 4.0 Hz, 1H, 5′-H_ax_or 3′-H_ax_], 1.16 (dq, *J* = 12.0 Hz, *J*′ = 4.0 Hz, 2H, 3′-H_ax_ or 5′-H_ax_), 1.83–2.04 [complex signal, 6H, 10(13)-H_ax_, 6-H_ax_, 12-H_ax_, 5′-H_eq_ or 3′-H_eq_], 2.06 (s, 3H, COCH_3_), 2.09–2.26 [complex signal, 6H, 8-H_2_, 10(13)-H_eq_, 6-H_eq_, 12-H_eq_], 2.71 (m, 1H, 6′-H_ax_ or 2′-H_ax_), 3.12 (m, 1H, 2′-H_ax_ or 6′-H_ax_), 3.21 [t, *J* = 7.2 Hz, 2H, 5(11)-H], 3.69–3.77 (complex signal, 2H, 4′-H, 6′-H_eq_ or 2′-H_eq_), 4.42 (dm, *J* = 13.6 Hz, 1H, 2′-H_eq_ or 6′-H_eq_), 4.71 (d, *J* = 7.6 Hz, 1H, C4′-NH), 4.82 (s, 1H, C7–NH), 7.06 [m, 2H, 1(4)-H], 7.10 [m, 2H, 2(3)-H]. ^13^C NMR (100.6 MHz, CDCl_3_): δ 21.4 (CH_3_, COCH_3_), 32.3 (CH_2_, C5′ or C3′), 33.7 (C3′ or C5′), 39.3 (CH_2_, d, ^4^*J*_C–F_ = 2.2 Hz, C6 or C12), 39.3 (CH_2_, d, ^4^*J*_C–F_ = 2.2 Hz, C12 or C6), 39.6 [CH, d, ^3^*J*_C–F_ = 13.3 Hz, C5(11)], 40.1 [CH_2_, d, ^2^*J*_C–F_ = 20.1 Hz, C10(13)], 40.7 (CH_2_, C6′ or C2′), 45.4 (CH_2_, C2′ or C6′), 46.6 (CH, C4′), 46.8 (CH_2_, C8), 56.8 (C, d, ^3^*J*_C–F_ = 11.4 Hz, C7), 94.4 (C, d, ^1^*J*_C–F_ = 176.9, C9), 126.8 [CH, C2(3)], 128.1 [CH, C1(4)], 144.8 [C, d, ^4^*J*_C–F_ = 2.0 Hz, C4a(11a)], 156.2 (C, NHCONH), 169.1 (C, COCH_3_). Anal. Calcd for C_23_H_30_FN_3_O_2_·0.5 H_2_O: C, 67.62; H, 7.65; N, 10.29. Found: C, 67.61; H, 7.93; N, 9.94. HRMS calcd for [C_23_H_30_FN_3_O_2_ + H]^+^, 400.2395; found, 400.2395.

#### 4.1.13 1-(9-Fluoro-5,6,8,9,10,11-hexahydro-7*H*-5,9:7,11-dimethanobenzo[9]annulen-7-yl)-3-(1-(tetrahydro-2*H*-pyran-4-carbonyl)piperidin-4-yl)urea (**21**)

To a solution of 9-fluoro-5,6,8,9,10,11-hexahydro-7*H*-5,9:7,11-dimethanobenzo[9]annulen-7-amine hydrochloride (150 mg, 0.56 mmol) in DCM (4.5 mL) and saturated aqueous NaHCO_3_ solution (3.5 mL), triphosgene (61.5 mg, 0.21 mmol) was added. The biphasic mixture was stirred at room temperature for 30 min, then the two phases were separated, and the organic layer was washed with brine (3.5 mL), dried over anhYd Na_2_SO_4_, filtered, and evaporated under vacuum to obtain 1–2 mL of a solution of the isocyanate in DCM. To this solution was added (4-aminopiperidin-1-yl) (tetrahydro-2*H*-pyran-4-yl)methanone (119 mg, 0.56 mmol). The mixture was stirred overnight at room temperature, and the solvent was then evaporated. Column chromatography (SiO_2,_ DCM/methanol mixtures) provided urea **21** as a yellowish solid (75 mg, 28% yield), mp 210–213 °C. IR (ATR): 3351, 2926, 2850, 1609, 1549, 1444, 1358, 1306, 1210, 1125, 1089, 1005, 983, 867, 760 cm^–1^. ^1^H NMR (400 MHz, CDCl_3_): δ 1.17 [dq, 2H, *J* = 12.0 Hz, *J*′ = 4.0 Hz, 3′(5′)-H_ax_], 1.56 [t, 2H, *J* = 10.8 Hz, 3″(5″)-H_ax_], 1.76–2.00 [complex signal, 7H, 10(13)-H_ax_, 6(12)-H_ax_, 3″(5″)-H_eq_, 3′-H_eq_ or 5′-H_eq_], 2.02–2.20 [complex signal, 5H, 10(13)-H_eq_, 6(12)-H_eq_, 5′-H_eq_ or 3′-H_eq_], 2.21 (d, 2H, *J* = 6.0 Hz, 8-H_2_), 2.65–2.80 (complex signal, 2H, 4″-H, 2′-H_ax_ or 6′-H_ax_), 3.11 (t, 1H, *J* = 12.4 Hz, 6′-H_ax_ or 2′-H_ax_), 3.21 [broad signal, s, 2H, 5(11)-H], 3.43 [t, 2 H, *J* = 11.2 Hz, 2″(6″)-H_ax_], 3.75 (m, 1H, 4′-H), 3.83 (d, 1H, *J* = 13.2 Hz, 2′-H_eq_ or 6′-H_eq_), 3.99 [dd, 2H, *J* = 11.6 Hz, *J*′= 2.0 Hz, 2″(6″)-H_eq_], 4.47 (d, 1H, *J* = 14.0 Hz, 6′-H_eq_ or 2′-H_eq_), 4.55 (d, 1H, *J* = 7.6 Hz, C4′-NH), 4.64 (s, 1H, C7–NH), 7.06 [m, 2H, 1(4)-H], 7.11 [m, 2H, 2(3)-H]. ^13^C NMR (100.6 MHz, CDCl_3_): δ 29.1 [CH_2_, C3″(5″)], 32.4 (CH_2_, C3′ or 5′), 34.2 (CH_2_, C5′ or 3′), 37.6 (CH, C4″), 39.3 [CH_2_, C6(12)], 39.5 [CH, ^3^*J*_C–F_ = 13.4 Hz, C5(11)], 40.1 [CH_2_, d, ^2^*J*_C–F_ = 20.1 Hz, C10(13)], 41.1 (CH_2_, C2′ or 6′), 44.3 (CH_2_, C2′ or 6′), 46.7 (CH_2_, d, ^2^*J*_C–F_ = 17.9 Hz, C8), 46.9 (CH, C4′), 56.9 (C, d, ^3^*J*_C–F_ = 11.5 Hz, C7), 67.1 (CH_2_, C2″(6″)], 94.4 [C, d, ^1^*J*_C–F_ = 177.2 Hz, C9), 126.8 [CH, C1(4)], 128.1 [CH, C2(3)], 144.8 [C, C1′(4′)], 156.0 (C, NHCONH), 172.9 (C, NCOR). Anal. Calcd for C_27_H_36_FN_3_O_3_·0.2 CH_2_Cl_2_: C, 67.14; H, 7.54; N, 8.64. Found: C, 67.47; H, 7.57; N 8.29. HRMS calcd for [C_27_H_36_FN_3_O_3_ + H], 470.2813; found, 470.2815. HPLC: *t*_r_ = 4.522 (λ = 220 nm, 98.8% purity).

#### 4.1.14 1-(9-Fluoro-5,6,8,9,10,11-hexahydro-7*H*-5,9:7,11-dimethanobenzo[9]annulen-7-yl)-3-(1-(cyclopropanecarbonyl)piperidin-4-yl)urea (**22**)

To a solution of 9-fluoro-5,6,8,9,10,11-hexahydro-7*H*-5,9:7,11-dimethanobenzo[9]annulen-7-amine hydrochloride (150 mg, 0.56 mmol) in DCM (4.5 mL) and saturated aqueous NaHCO_3_ solution (3.5 mL), triphosgene (61.5 mg, 0.21 mmol) was added. The biphasic mixture was stirred at room temperature for 30 min, then the two phases were separated, and the organic layer was washed with brine (3.5 mL), dried over anhyd Na_2_SO_4_, filtered, and evaporated under vacuum to obtain 1–2 mL of a solution of the isocyanate in DCM. To this solution was added (4-aminopiperidin-1-yl) (tetrahydro-2*H*-pyran-4-yl)methanone (94.2 mg, 0.56 mmol). The mixture was stirred overnight at room temperature, and the solvent was then evaporated. Column chromatography (SiO_2,_ DCM/methanol mixtures) provided urea **22** as a white solid (60 mg, 25% yield), mp 187–191 °C. IR (ATR): 3320, 2934, 1630, 1568, 1450, 1358, 1317, 1221, 1125, 865, 767, 734, 569. ^1^H NMR (400 MHz, CDCl_3_): δ 0.75 [dd, 2H, *J* = 8.0 Hz, *J*′ = 3.2 Hz, 8′(9′)-H_ax_], 0.93 [dd, 2H, *J* = 9.6 Hz, *J*′ = 4.8 Hz, 8′(9′)-H_eq_], 1.20 [complex signal, 2H, 3′(5′)-H_ax_], 1.74 (tt, 1H, *J* = 8.0 Hz, *J*′ = 4.8 Hz, 7′-H), 1.95–1.85 [d, 4H, *J* = 12.8 Hz, 10(13)-H_ax_, 6(12)-H_ax_; d, 1H, *J* = 12.4 Hz, 3′ or 5′-H_eq_], 2.2–2.1 [complex signal, 5H, 10(13)-H_eq_, 6(12)-H_eq_, 3′ or 5′-H_eq_], 2.25 (d, 2H, *J* = 5.2 Hz, 8-H), 2.75 (t, 2H, *J* = 12 Hz, 2′ or 6′-H_ax_), 3.20 [m, 3H, 5(11)-H, 2′ or 6′-H_ax_], 3.75 (m, 1H, 4′-H), 4.10 (broad signal, d, 1H, *J* = 14 Hz, 2′ or 6′-H_eq_), 4.37 (d, 1H, *J* = 7.6 Hz, HNCONH), 4.50–4.45 (s, 1H, HNCONH; s, 1H, 2′ or 6′-H_eq_), 7.07 [broad signal, 2H, 2(3)-H], 7.11 [broad signal, 2H, 1(4)-H]. ^13^C NMR (100.5 MHz, CDCl_3_): δ 7.51 [CH_2_, C8′(9′)], 11.15 (CH, C7′), 32.54 (CH_2_, C5′ or 3′), 34.41 (CH_2_, C5′ or 3′), 39.47 [CH, C5(11)], 39.80 [CH_2_, d, ^4^*J*_C–F_ = 14.07 Hz, C6(12)], 40.34 [CH_2_, d, ^2^*J*_C–F_ = 20.1 Hz, C10(13), 41.66 (CH_2_, C2′ or 6′), 44.71 (CH_2_, C2′ or 6′), 46.94 (CH_2_, d, ^2^*J*_C–F_ = 18.09 Hz, C8), 47.22 (CH, C4′), 57.19 (C, C7), 94.53 (C, d, ^1^*J*_C–F_ = 176.88 Hz, C9), 126.99 [CH, C1(4)], 128.27 [CH, C2(3)], 144.97 [CH, C1′(4′)], 156.09 (C, HNCONH), 172.26 (C, CO). Anal. Calcd for C_25_H_32_FN_3_O_2_·0.1 CH_2_Cl_2_: C, 69.46; H, 7.48; N, 9.68. Found: C, 69.64; H, 7.52; N, 9.45. Accurate mass calcd for [C_25_H_32_FN_3_O_2_ + H]^+^, 426.2551; found, 426.2556.

#### 4.1.15 1-(1-Acetylpiperidin-4-yl)-3-(9-hydroxy-5,6,8,9,10,11-hexahydro-7*H*-5,9:7,11-dimethanobenzo[9]annulen-7-yl)urea (**23**)

To a solution of 1-(4-aminopiperidin-1-yl)ethan-1-one (192 mg, 1.35 mmol) in DCM (4 mL) and saturated aqueous NaHCO_3_ solution (3 mL), triphosgene (200 mg, 0.67 mmol) was added. The biphasic mixture was stirred at room temperature for 30 min, then the two phases were separated, and the organic one was washed with brine (5 mL), dried over anhyd Na_2_SO_4_, filtered, and evaporated under vacuum to obtain 1–2 mL of a solution of the isocyanate in DCM. To this solution was added 9-amino-5,6,8,9,10,11-hexahydro-7*H*-5,9:7,11-dimethanobenzo[9]annulen-7-ol hydrochloride (300 mg, 1.13 mmol) followed by Et_3_N (228 mg, 2.25 mmol). The reaction mixture was stirred at room temperature overnight, and the solvent was evaporated under vacuum. Column chromatography (SiO_2_, DCM/methanol mixtures) gave urea **23** (19 mg, 4.2% yield) as a gray solid, mp 222–223 °C. IR (NaCl disk): 3313, 2922, 2852, 1733, 1716, 1699, 1646, 1622, 1558, 1542, 1507, 1491, 1472, 1456, 1358, 1337, 1319, 1301, 1265, 1231, 1204, 1134, 1104, 1053 cm^–1^. ^1^H NMR (400 MHz, CDCl_3_): δ 1.20 [m, 2H, 3′(5′)-H_ax_], 1.76 [d, *J* = 12.8 Hz, 2H, 6(12)-H_ax_], 1.86–2.02 [complex signal, 6H, 3′(5′)-H_eq_, 10(13)-H_ax_, 6(12)-H_eq_], 2.04 (s, 2H, 8-H), 2.07 (3, 3H, COCH_3_), 2.14 [m, 2H, 10(12)-H_eq_], 2.70 (m, 1H, 6′-H_ax_ or 2′-H_ax_), 3.12 [ddd, *J* = 14.4 Hz, *J*′ = 12.0 Hz, *J*″ = 2.4 Hz, 1H, 2′-H_ax_ or 6′-H_ax_], 3.17 [t, *J* = 6.0 Hz, 2H, 5(11)-H], 3.67–3.78 [complex signal, 2H, 4′-H, 2′-H_eq_ or 6′-H_eq_], 4.24 (d, *J* = 7.6 Hz, 1H, C4′-NH), 4.34 (s, 1H, C7–NH), 4.47 [dm, *J* = 14.0 Hz, 1H, 6′-H_eq_ or 2′-H_eq_), 7.06 [m, 2H, 1(4)-H)], 7.09 [m, 2H, 2(3)-H]. ^13^C NMR (100.6 MHz, CDCl_3_): δ 21.4 (CH_3_, COCH_3_), 32.4 (CH_2_, C5′ or C3′), 33.6 (CH_2_, C3′ or C5′), 39.4 [CH_2_, C10(13)], 40.1 [CH, C5(11)], 40.7 (CH_2_, C6′ or C2′), 42.5 [CH_2_, C6(12)], 45.4 (CH_2_, C2′ or C6′), 47.1 (CH, C4′), 49.1 (CH_2_, C8), 56.3 (C, C7), 71.0 (C, C9), 126.6 [CH, C2(3)], 128.1 [CH, C1(4)], 145.2 [C, C4a(11a)], 155.9 (C, NHCONH), 169.0 (C, COCH_3_). Anal. Calcd for C_23_H_31_N_3_O_3_·CH_3_OH: C, 67.11; H, 8.21; N, 9.78. Found: C, 67.25; H, 8.15; N, 9.72. HRMS calcd for [C_23_H_31_N_3_O_3_ + H]^+^, 398.2438; found, 398.2440.

#### 4.1.16 1-(1-Acetylpiperidin-4-yl)-3-(9-methoxy-5,6,8,9,10,11-hexahydro-7*H*-5,9:7,11-dimethanobenzo[9]annulen-7-yl)urea (**24**)

To a solution of 9-methoxy-5,6,8,9,10,11-hexahydro-7*H*-5,9:7,11-dimethanobenzo[9]annulen-7-amine (300 mg, 1.23 mmol) in DCM (4.5 mL) and saturated aqueous NaHCO_3_ solution (3 mL), triphosgene (183 mg, 0.62 mmol) was added. The biphasic mixture was stirred at room temperature for 30 min, then the two phases were separated, and organics were washed with brine (5 mL), dried over anhyd Na_2_SO_4_, filtered, and evaporated under vacuum to obtain 1–2 mL of a solution of the isocyanate in DCM. To this solution was added 1-(4-aminopiperidin-1-yl)ethan-1-one (210 mg, 1.47 mmol). The reaction mixture was stirred at room temperature overnight, and the solvent was evaporated under vacuum to obtain a white gum (521 mg). Column chromatography (SiO_2_, DCM/methanol mixtures) gave urea **24** (148 mg, 30% yield) as a white solid. The analytical sample was obtained by crystallization from hot EtOAc, mp 212–213 °C. IR (NaCl disk): 3358, 3044, 3019, 2931, 2847, 2823, 1646, 1618, 1555, 1495, 1452, 1356, 1319, 1266, 1229, 1135, 1095, 1076, 972, 849, 756, 735 cm^–1^. ^1^H NMR (400 MHz, CDCl_3_): δ 1.12 (dq, *J* = 11.6 Hz, *J*′ = 4.0 Hz, 1H, 3′-H_ax_ or 5′-H_ax_), 1.19 (dq, *J* = 11.6 Hz, *J*′ = 4.0 Hz, 1H, 5′-H_ax_ or 3′-H_ax_), 1.79–1.86 [complex signal, 3H, 6(12)-H_ax_, 5′-H_eq_ or 3′-H_eq_], 1.92 [dm, *J* = 12.4 Hz, 6(12)-H_eq_], 1.98–2.02 [complex signal, 5H, 10(13)-H_ax_, 8-H_2_, 5′-H_eq_ or 3′-H_eq_], 2.06 (s, 3H, COCH_3_), 2.10 [m, 2H, 10(13)-H_eq_], 2. 70 (m, 1H, 6′-H_ax_ or 2′-H_ax_), 3.10 [ddd, *J* = 14.4 Hz, *J*′ = 12.4 Hz, *J*″ = 2.4 Hz, 1H, 2′-H_ax_ or 6′-H_ax_], 3.17 [t, *J* = 6.0 Hz, 2H, 5(11)-H], 3.22 (s, 3H, OCH_3_), 3.68–3.77 (complex signal, 2H, 4′-H, 6′-H_eq_ or 2′-H_eq_), 4.41 (dm, *J* = 13.6 Hz, 1H, 2′-H_eq_ or 6′-H_eq_), 4.73 (d, *J* = 8.0 Hz, 1H, C4′-NH), 4.76 (s, 1H, C7–NH), 7.05 [m, 2H, 1(4)-H], 7.08 (m, 2H, 2(3)-H]. ^13^C NMR (100.6 MHz, CDCl_3_): δ 21.4 (CH_3_, COCH_3_), 32.4 (CH_2_, C5′ or C3′), 33.7 (CH_2_, C3′ or C5′), 38.2 [CH_2_, C6(12)], 39.71 (CH_2_, C10 or C13), 39.73 (CH_2_, C13 or C10), 39.8 [CH, C5(11)], 40.7 (CH_2_, C6′ or C2′), 45.4 (CH_2_, C2′ or C6′), 45.6 (CH_2_, C8), 46.6 (CH, C4′), 48.2 (CH_3_, OCH_3_), 55.8 (C, C7), 74.8 (C, C9), 126.6 CH, C2(3)], 128.0 [CH, C1(4)], 145.4 [C, C4a(11a)], 156.3 (C, NHCONH), 169.1 (C, COCH_3_). Anal. Calcd for C_24_H_33_N_3_O_3_: C, 70.04; H, 8.08; N, 10.21. Found: C, 69.63; H, 8.28; N, 9.86. HRMS calcd for [C_24_H_33_N_3_O_3_ +H]^+^, 412.2595; found, 412.2595.

#### 4.1.17 1-(5,6,8,9,10,11-Hexahydro-7*H*-5,9:7,11-dimethanobenzo[9]annulen-7-yl-9-d)-3-(1-(tetrahydro-2*H*-pyran-4-carbonyl)piperidin-4-yl)urea (**25**)

To a solution of 5,6,8,9,10,11-hexahydro-7*H*-5,9:7,11-dimethanobenzo[9]annulen-9-*d*-7-amine hydrochloride (82 mg, 0.32 mmol) in DCM (2 mL) and saturated aqueous NaHCO_3_ solution (2 mL), triphosgene (36 mg, 0.12 mmol) was added. The biphasic mixture was stirred at room temperature for 30 min, then the two phases were separated, and the organic one was washed with brine (3 mL), dried over anhyd Na_2_SO_4_, filtered, and evaporated under vacuum to obtain 1–2 mL of a solution of isocyanate in DCM. To this solution was added (4-aminopiperidin-1-yl) (tetrahydro-2*H*-pyran-4-yl)methanone (68 mg, 0.32 mmol). The mixture was stirred overnight at room temperature, and the solvent was then evaporated. Column chromatography (SiO_2_, DCM/Methanol mixtures) provided urea **25** as a white solid (83 mg, 56% yield). The analytical sample was obtained by crystallization from hot EtOAc, mp 125–126 °C. IR (ATR): 3318, 2902, 2849, 1630, 1557, 1491, 1445, 1361, 1318, 1300, 1274, 1238, 1213, 1123, 1108, 1090, 1040, 1016, 987, 972, 872, 823, 750 cm^–1^. ^1^H NMR (400 MHz, CDCl_3_): δ 1.17 [dt, *J* = 12.0 Hz, *J*′ = 4.0 Hz, 2H, 3′-H_ax_ or 5′-H_ax_], 1.20 [dt, *J* = 12.0 Hz, *J*′ = 4.0 Hz, 2H, 5′-H_ax_ or 3′-H_ax_], 1.56 [m, 2H, 3″(5″)-H_ax_], 1.73 [d, *J* = 13.2 Hz, 2H, 10(13)-H_ax_], 1.80–1.90 [complex signal, 3H, 3″(5″)-H_eq_, 5′-H_eq_ or 3′-H_eq_), 1.92 [dd, *J* = 13.2 Hz, *J*′ = 6.0 Hz, 2H, 10(13)-H_eq_], 1.98–2.10 [complex signal, 5H, 6(12)-H_ax_, 8-H_2_, 3′-H_eq_ or 5′-H_eq_], 2.18 [m, 2H, 6(12)-H_eq_], 2.65–2.76 [complex signal, 2H, 4″-H, 2′-H_ax_ or 6′-H_ax_], 3.03 [t, *J* = 6.0 Hz, 2H, 5(11)-H], 3.10 (m, 1H, 6′-H_ax_ or 2′-H_ax_), 3.43 [m, 2H, 2″(6″)-H_ax_], 3.75 (m, 1H, 4′-H), 3.82 (d, *J* = 13.0 Hz, 1H, 6′-H_eq_ or 2′-H_eq_), 3.99 [dm, *J* = 11.4 Hz, 2H, 2″(6″)-H_eq_], 4.27–4.34 [complex signal, 2H, C7–NH, C4′-NH], 4.48 [d, *J* = 13.0 Hz, 1H, 2′-H_eq_ or 6′-H_eq_], 7.03 [m, 2H, 1(4)-H], 7.05 [m, 2H, 2(3)-H]. ^13^C NMR (100.6 MHz, CDCl_3_): δ 29.2 [CH_2_, C3″(5″)], 30.7 (CD, t, ^1^*J*_C–D_ = 19.8 Hz, C9), 32.4 (CH_2_, C5′ or C3′), 34.1 (CH_2_, C3′ or C5′), 34.3 [CH_2_, C10(13)], 37.6 (CH, C4″), 40.5 [CH_2_, C6(12)], 41.1 [CH_2_, C2′ or C6′), 41.2 [CH, C5(11) and CH_2_, C8], 44.3 (CH_2_, C6′ or C2′), 47.1 (CH, C4′), 51.9 (C, C7), 67.2 [CH_2_, C2″(6″)], 126.2 [CH, C2(3)], 128.0 [CH, C1(4)], 146.6 [C, C4a(11a)], 156.1 (C, NHCONH), 172.8 (C, NCOR). HRMS calcd for [C_27_H_36_DN_3_O_3_ + H]^+^, 453.297; found, 453.2974. Anal. Calcd for C_27_H_36_DN_3_O_3_·1 H_2_O: C, 68.91; H, 8.14; N, 8.93. Found: C, 69.28; H, 7.94; N 8.69.

#### 4.1.18 *tert*-Butyl 4-(3-(9-chloro-5,6,8,9,10,11-hexahydro-7*H*-5,9:7,11-dimethanobenzo[9]annulen-7-yl)ureido)piperidine-1-carboxylate (**26**)

To a solution of 9-chloro-5,6,8,9,10,11-hexahydro-7*H*-5,9:7,11-dimethanobenzo[9]annulen-7-amine hydrochloride (131 mg, 0.46 mmol) in DCM (4 mL) and saturated aqueous NaHCO_3_ solution (3 mL), triphosgene (50 mg, 0.17 mmol) was added. The biphasic mixture was stirred at room temperature for 30 min, then the two phases were separated, and the organic one was washed with brine (5 mL), dried over anhyd Na_2_SO_4_, filtered, and evaporated under vacuum to obtain 1–2 mL of a solution of isocyanate in DCM. To this solution was added 4-amino-1-Boc-piperidine (93 mg, 0.46 mmol). The mixture was stirred overnight at room temperature, and the solvent was then evaporated. Column chromatography (SiO_2_, DCM/Methanol mixtures) provided **26** as a white solid (103 mg, 47% yield). HRMS–ESI^–^*m*/*z*: [M – H]^−^ calcd for [C_26_H_36_ClN_3_O_3_ – H]^−^, 472.2372; found, 472.2365.

#### 4.1.19 1-(9-Chloro-5,6,8,9,10,11-hexahydro-7*H*-5,9:7,11-dimethanobenzo[9]annulen-7-yl)-3-(piperidin-4-yl)urea (**27**)

*t*-Butyl 4-(3-(9-chloro-5,6,8,9,10,11-hexahydro-7*H*-5,9:7,11-dimethanobenzo[9]annulen-7-yl)ureido)piperidine-1-carboxylate (19.74 mg, 41.6 μmol) was dissolved in 5 mL dichloromethane and 20 μL of TFA was added and the reaction stirred until TLC showed no starting material. The solvents were evaporated, and the free amine **27** (ESI–MS calcd for C_21_H_28_ClN_3_O [M + H^+^] *m*/*z*: 374.19; found, 374.05) was used in the next step.

#### 4.1.20 1-(1-(3-(3-(But-3-yn-1-yl)-3*H*-diazirin-3-yl)propanoyl)piperidin-4-yl)-3-(9-chloro-5,6,8,9,10,11-hexahydro-7*H*-5,9:7,11-dimethanobenzo[9]annulen-7-yl)urea (**28**)

1-(9-Chloro-5,6,8,9,10,11-hexahydro-7*H*-5,9:7,11-dimethanobenzo[9]annulen-7-yl)-3-(piperidin-4-yl)urea (41.6 μmol, 1.2 equiv) was dissolved in 250 μL dimethylformamide and 20 μL *N*,*N*-diisopropylethylamine was added. In a separate flask 3-(3-(but-3-yn-1-yl)-3*H*-diazirin-3-yl)propanoic acid (34.6 μmol, 1 equiv) was dissolved in 250 μL dimethylformamide to which 1-[bis(dimethylamino)methylene]-1*H*-1,2,3-triazolo[4,5-*b*]pyridinium 3-oxide hexafluorophosphate (HATU; 34 μmol, 0.9 equiv) and *N*,*N*-diisopropylethylamine (20 μL, 3 equiv) were added. After stirring for 10 min, the solution containing the amine was added to the mixture, which was left stirring at room temperature for 16 h. Reverse phase HPLC purification provided **28** in a 66% yield. ESI–MS calcd for C_29_H_36_ClN_5_O_2_ [M + H]^+^*m*/*z*: 522.26; found, 522.10. ^1^H NMR (CDCl_3_): δ (ppm) 1.18–1.28 (m, 3H), 1.66 (td, 2H), 1.84 (m, 2H), 1.18–2.06 (cs, 12H), 1.16 (m, 4H), 2.23 (d, 2H), 2.71 (dt, 1H), 3.06 (dt, 1H), 3.23 (s, 2H), 3.69–3.74 (cs, 2H), 4.47 (d, 2H), 7.06–7.09 (m, 2H), 7.11–7.14 (m, 2H). ^13^C NMR (CDCl_3_): δ (ppm) 13.3, 26.8, 27.9, 32.3, 32.5, 33.3, 39.3, 39.5, 39.6, 39.9, 40.1, 40.9, 44.3, 46.6, 46.7, 47.4, 57.2, 57.3, 69.2, 82.8, 93.7, 94.9, 126.9, 128.2, 144.7, 155.8, 169.5.

### 4.2 In Vitro Biological Methods

The assays for the in vitro determination of the inhibitory activities toward human and mouse sEH,^30^ the assays for determination of the PAMPA–BBB permeability,^36^ aqueous solubility, cytotoxicity in SH-SY5Y cells, microsomal stability, cytochrome P450 inhibition, permeability, hERG inhibition, and inhibition of human LOX-5 were carried out following described methodologies previously used in our group (see the Supporting Information for complete details).^21,22^

### 4.3 In Silico Studies

#### 4.3.1 MD Simulation Details

The parameters for **13**, **15**, **21**, and **23** for the MD simulations were generated using the ANTECHAMBER module of AMBER 18^51^ using the general AMBER force field (GAFF),^52^ with partial charges set to fit the electrostatic potential generated at the HF/6-31G(d) level by the RESP model.^53^ The charges were calculated according to the Merz–Singh–Kollman scheme^54,55^ using Gaussian 09.^56^

MD simulations of sEH were carried out using PDB 5AM3 (crystallized with *t*-AUCB) and PDB 5ALZ (crystallized with piperidine-based sEHI) as a starting point.^31^ The benzohomoadamanatane derivatives corresponding to **13**, **15**, **21**, **23**, and **24** were manually prepared using the *t*-AUCB structure as a starting point. Molecular docking calculations using the standard parameters of the SwissDock web server were carried out to assess to generate starting orientations of **13**, **15**, **21**, **23**, and **24**.^57,58^ The coordinates of *t*-AUCB in PDB 5AM3 and piperidine-base compound in PDB 5ALZ were a reference for placing compounds **13**, **15**, **21**, **23**, and **24** in molecular docking calculations in the two possible orientations: (a) benzohomoadamantane group in the lhs while the piperidine group in the rhs and (b) benzohomoadamantane group in the rhs while piperidine in the lhs. From these two sets of orientations, conventional MD simulations were used to explore the conformational plasticity of sEH in the presence of **13**, **15**, **21**, **23**, and **24** bound in the active site. All simulations were performed using the AMBER ff14SB force field.^59^ Amino acid protonation states were predicted using the H++ server (http://biophysics.cs.vt.edu/H++). The MD simulations have been carried with the following protonation of histidine residues: HIE146, HIE239, HIP251, HID265, HIP334, HIE420, HIE506, HIE513, HIE518, and HIP524.

Each system was immersed in a pre-equilibrated truncated octahedral box of water molecules with an internal offset distance of 10 Å. All systems were neutralized with explicit counterions (Na^+^ or Cl^–^). A two-stage geometry optimization approach was performed. First, a short minimization of the positions of water molecules with positional restraints on the solute by a harmonic potential with a force constant of 500 kcal mol^–1^ Å^–2^ was done. The second stage was an unrestrained minimization of all the atoms in the simulation cell. Then, the systems were gently heated in six 50 ps steps, increasing the temperature by 50 K each step (0–300 K) under constant-volume, periodic-boundary conditions, and the particle-mesh Ewald approach^60^ to introduce long-range electrostatic effects. For these steps, a 10 Å cutoff was applied to Lennard–Jones and electrostatic interactions. Bonds involving hydrogen were constrained with the SHAKE algorithm.^61^ Harmonic restraints of 10 kcal mol^–1^ were applied to the solute, and the Langevin equilibration scheme was used to control and equalize the temperature.^62^ The time step was kept at 2 fs during the heating stages, allowing potential inhomogeneities to self-adjust. Each system was then equilibrated for 2 ns with a 2 fs timestep at a constant pressure of 1 atm (NPT ensemble). Finally, conventional MD trajectories at a constant volume and temperature (300 K) were collected. In total, we carried out three replicas of 500 ns MD simulations for sEH in the presence of **13**, **15**, **21**, **23**, and **24** gathering a total of 7.5 μs of MD simulation time. Each MD simulation was clusterized based on active site residues, and the structures corresponding to the most populated clusters were used for the noncovalent interactions analysis. We monitored the presence of water molecules using the watershell function of the cpptraj MD analysis program.^63^ aMD simulations^64,65^ were used to study the spontaneous binding of **15** in the active site of sEH. Standard dual-boost aMD simulations were performed using the same simulation protocols and aMD parameters as described in our previous works.^21^ To reconstruct the spontaneous binding process, we placed one molecule of **15** in the solvent with a minimum distance of 25 Å from catalytic Asp335. First, we performed 250 ns of conventional MD followed by 10 replicas of 2 μs of aMD capturing one binding event (see Movie S1 comprising only the aMD simulation part). Binding affinities (kcal/mol) of compounds **13**, **15**, **21**, and **23** were computed using the MM/GBSA method as implemented in AMBER 18.

### 4.4 Preparation of HEK 293T Lysates

HEK293T cells were grown in DMEM media (D6546-500ML Sigma) supplemented with 10% FBS, 2 mM glutamax, 100 units/mL penicillin, and 0.1 mg/mL streptomycin. They were maintained at 37 °C with 5% CO_2_. Cells were split every 3 to 4 days according to an ATCC protocol. The cells were harvested and collected by centrifugation (500 g for 5 min at 4 °C) and the supernatant was removed. The pellets were washed twice with ice-cold PBS and resuspended in 2 vol of ice-cold lysis buffer (50 mM HEPES pH 7.5, 150 mM NaCl, 1 mM DTT and 0.5% NP-40). After 30 min on ice, the cells were centrifuged to remove cell debris for 5 min at 4 °C. The supernatant was aliquoted and flash frozen in liquid N_2_ for use as lysates, with a total protein concentration of 1 mg/mL. Protein concentrations were determined using the BCA assay (Fisher Scientific).

### 4.5 Labeling in HEK 293T Lysates

HEK 293T lysates were spiked or not with 100 ng of recombinant purified sEH, treated either with 100 nM probe **28** or DMSO, and incubated for 30 min at 37 °C. After this time, the samples were irradiated for 6 min at 365 nm using a 100 W UV lamp. Subsequently, a bi-functional tag containing a TAMRA dye and a biotin was incorporated using copper(I)-catalyzed azide–alkyne cycloaddition (CuAAC). The photoaffinity labeling was analyzed by in-gel analysis, mixing the samples with 4× SDS-loading buffer, and separating using 12% SDS-PAGE after which the gel was scanned on a Typhoon FLA 9500.

### 4.6 Labeling Purified Soluble Epoxide Hydrolase for Minimal Probe Concentration Determination

Purified recombinant sEH was produced and purified as indicated previously.^30^ Of the pure active enzyme 100 or 200 ng were incubated for 30 min at 37 °C with decreasing concentrations of probe 3, namely: 10 μM, 1 μM, 100 nM, 10 nM, and 1 nM. After this time, the compounds were irradiated for 6 min at 365 nm using a 100 W UV lamp. Subsequently, a bi-functional tag containing a TAMRA dye and a biotin was incorporated using copper(I)-catalyzed azide–alkyne cycloaddition (CuAAC). The photoaffinity labeling was analyzed by in-gel analysis by mixing the samples with 4× SDS-loading buffer and separating using 12% SDS-PAGE after which the gel was scanned on a Typhoon FLA 9500.

### 4.7 EPHX2 Target Engagement Confirmation and Off-Target Elucidation by Pull Down

Untreated HEK293T whole cell lysates were normalized to a concentration of 1 mg/mL in a volume of 100 μL, per condition. Lysates were then treated with DMSO, 10 μM of probe **28** or 10 μM of probe **28**, and 100 μM of **15** (for competition experiments), and incubated at 37 °C for 30 min. After this time, the whole was irradiated for 6 min at 365 nm using a 100 W UV lamp. Subsequently, a bi-functional tag containing a TAMRA dye and a biotin was incorporated via CuAAC. The excess reagents from the samples were then removed by acetone precipitation. Following resuspension of the pellets to a final volume of 100 μL, half of the sample was kept as the input control. The remaining 50 μL were incubated with 20 μL of pre-washed streptavidin beads (Thermo Fisher) for 1 h with mixing at RT. The supernatant was removed, and the beads were sequentially washed with 0.33% SDS in PBS (2 × 50 μL), 1 M NaCl (2 × 50 μL) and PBS (2 × 50 μL). Bound proteins were eluted by boiling (95 °C) the beads with 60 μL of 1× SDS loading buffer for 10 min. Samples were resolved by 12% SDS-PAGE. Following visualization using a Typhoon FLA 9500, the gel was transferred onto a nitrocellulose membrane and probed with VEGF2 (cell signaling), p38 MAPK (cell signaling), EPHX1 (Elabscience), and EPHX2 (Abcam) for detection.

This experiment was also carried out using lower probe and parent compound concentrations of 1 and 10 μM, respectively, yielding the same results.

### 4.8 Affinity-Based Probe and Parent Compound Off-Target Profile Elucidation

To HEK293T cell lysates at 1 mg/mL protein concentration spiked or not with 100 ng of recombinant human sEH and 100 ng of purified recombinant enzyme were treated with either 100 nM probe **28**, 10 μM urea **15**, and 100 nM probe **28** or DMSO to a concentration of 1% of the total sample. After 30 min of incubation of the compounds at 37 °C, the whole was irradiated for 6 min at 365 nm using a 100 W UV lamp. Subsequently, a bi-functional tag containing a TAMRA dye and a biotin was incorporated via CuAAC. The samples were analyzed by in-gel analysis by mixing the samples with 4× SDS-loading buffer and separating using 12% SDS-PAGE after which the gel was scanned on a Typhoon FLA 9500 and/or submitted to Western blot analysis using human sEH antibody for detection (Abcam). The comparison of labeling patterns via fluorescence showed the inability of the parent compound to compete out the probe **28** for most of the targets, which pointed out that except for the sEH the other labeled proteins are not targets of the parent compound but of the probe **28**.

### 4.9 Pharmacokinetic Study

#### 4.9.1 Animals

For pharmacokinetic studies, 48 male CD1 mice (weight, 40 to 50 g; age, 8 week), obtained from Envugi, Madison, WI, USA, were housed 3 per cage in IVC (Thoren no. 9 cages (19.5 cm × 30.9 cm × 13.3 cm); Thoren Caging Systems, Hazleton, PA. Mice were kept in an environmentally controlled room: air replacement every 10 min, constant temperature (21 ± 3 °C), and humidity and on a 12/12 h day/night cycle. All experimental procedures followed the standard ethical guidelines of European Communities Council Directive 86/609/EEC and by the Institutional Animal Care and Use of Catalunya (10291, approved 1/28/2018).

#### 4.9.2 Drug Delivery, Sample Collection, and Sample Preparation

Formulations were prepared the day of the study. The vehicle was 10% of 2-hydroxypropyl-β-cyclodextrin (Sigma-Aldrich, ref. 332607-25G). Mice were administered with **15** or **21** at a dose of 5 mg/Kg by sc route. The volume of administration was 10 mL/kg. Animals were weighed before each administration to adjust the required volume. Blood samples (*n* = 3) were collected after euthanasia of mice at different times (0.25; 0.5; 1; 2; 3, 4, and 6 h). The plasma was separated by centrifugation for 10 min and stored at −80 °C until analysis by HPLC. Frozen plasma samples were thawed at room temperature and 25 μL of acetonitrile were added to a 100 μL of plasma sample. The sample was vortexed for 30 s and centrifuged (14,000 rpm/min) for 5 min. The supernatant was transferred to an injection bottle and 25 μL was injected into the chromatographic system.

#### 4.9.3 Instruments and Analysis Conditions

The HPLC system was a PerkinElmer LC (PerkinElmer INC, Massachusetts, U.S.) consisting of a Flexar LC pump, a chromatography interface (NCI 900 network), a Flexar LC autosampler PE, and a Waters 2487 dual λ absorbance detector. The chromatographic column was a kromasil 100-5-C18 (4,0 × 200 mm-Teknokroma Analitica S.A. Sant Cugat, Spain). Flow was 0.8 mL/min and the mobile phase consisting in 0.05 M KH_2_PO4 (30%)/acetonitrile (70%) in isocratic conditions. The elution times of **15** and **21** were 5.6 and 4.4 min, respectively. Compounds were detected at 220 nm. The assay had a range of 0.015–25 μg mL^–1^. The calibration curves were constructed by plotting the peak area ratio of analyzed peak against known concentrations. Compound **22** was analyzed under the same chromatographic conditions but the response to the analysis was 10 times lower than that of **15** and **21**.

#### 4.9.4 Pharmacokinetic Analysis

**15** and **51** plasma concentrations versus time curves for the means of animals were analyzed by a non-compartmental model based on the statistical moment theory using the “PK Solutions” computer program. The pharmacokinetic parameters calculated were as follows: area under the plot of plasma concentration versus time curve (AUC), calculated using the trapezoidal rule in the interval 0–6 h; HL (*t*_1/2β_), determined as ln_2/β_, being β, calculated from the slope of the linear, least-squares regression line; *C*_max_ and *T*_max_ were read directly from the mean concentration curves.

### 4.10 In Vivo Efficacy Studies

#### 4.10.1 Experimental Animals

Experiments were performed in female WT-CD1 (Charles River, Barcelona, Spain) mice weighing 25–30 g. Mice were acclimated in our animal facilities for at least 1 week before testing and were housed in a room under controlled environmental conditions: 12/12 h day/night cycle, constant temperature (22 ± 2 °C), air replacement every 20 min, and they were fed a standard laboratory diet (Harlan Teklad Research Diet, Madison, WI, USA) and tap water ad libitum until the beginning of the experiments. The behavioral test was conducted during the light phase (from 9.00 to 15.00 h), and randomly throughout the oestrous cycle. Animal care was in accordance with institutional (Research Ethics Committee of the University of Granada, Spain), regional (Junta de Andalucía, Spain), and international standards (European Communities Council Directive 2010/63).

#### 4.10.2 Drugs and Drug Administration

The sEHI were dissolved in 5% DMSO (Merck KGaA, Darmstadt, Germany) in physiological sterile saline (0.9% NaCl). Drug solutions were prepared immediately before the start of the experiments and injected sc in a volume of 5 mL/kg into the interscapular area. To test for the effects of MS-PPOH (Cayman Chemical Company, Ann Arbor, MI, USA), a selective inhibitor of microsomal CYP450 epoxidase,^45^ on the effects induced by the sEHI tested, this compound was dissolved in DMSO 5% and cyclodextrin 40% in saline and administered 5 min before sEHI injection. When the effect of the association of several drugs was assessed, each injection was performed in different areas of the interscapular zone to avoid the mixture of the drug solutions and any physicochemical interaction between them. In all cases, the researchers who performed the experiments were blinded to the treatment received by each animal.

As it will be detailed below, we used two different algogenic substances to explore the effects of sEHI on nociception: capsaicin was used to induce somatic mechanical hypersensitivity, and CTX to induce visceral pain. Capsaicin (Sigma-Aldrich Química S.A.) was dissolved in 1% DMSO in physiological sterile saline to a concentration of 0.05 μg/μL (i.e., 1 μg per mouse). Capsaicin solution was injected intraplantarly (i.pl.) into the right hind paw proximate to the heel, in a volume of 20 μL using a 1710 TLL Hamilton microsyringe (Teknokroma, Barcelona, Spain) with a 30^1/2^-gauge needle. Control animals were injected with the same volume of the vehicle of capsaicin. CTX (Sigma-Aldrich, Madrid, Spain), which was used to induce a painful cystitis, was dissolved in saline and injected ip at a dose of 300 mg/kg, in a volume of 10 ml/kg. The same volume of solvents was injected in control animals.

#### 4.10.3 Evaluation of Capsaicin-Induced Secondary Mechanical Hypersensitivity

Animals were placed into individual test compartments for 2 h before the test to habituate them to the test conditions. The test compartments had black walls and were situated on an elevated mesh-bottomed platform with a 0.5 cm^2^ grid to provide access to the ventral surface of the hind paws. In all experiments, punctate mechanical stimulation was applied with a dynamic plantar aesthesiometer (Ugo Basile, Varese, Italy) at 15 min after the administration of capsaicin or its solvent. Briefly, a nonflexible filament (0.5 mm diameter) was electronically driven into the ventral side of the paw previously injected with capsaicin or solvent (i.e., the right hind paw), at least 5 mm away from the site of the injection toward the fingers. The intensity of the stimulation was fixed at 0.5 g force, as described previously.^66^ When a paw withdrawal response occurred, the stimulus was automatically terminated, and the response latency time was automatically recorded. The filament was applied three times, separated by intervals of 0.5 min, and the mean value of the three trials was considered the withdrawal latency time of the animal. A cutoff time of 50 s was used. The compounds tested, or their solvent, were administered sc 30 min before the i.pl. administration of capsaicin or DMSO 1% (i.e., 45 min before we evaluated the response to the mechanical punctate stimulus).

#### 4.10.4 Evaluation of Cyclophosphamide-Induced Visceral Pain

CTX-evoked pain behaviors and referred hyperalgesia were examined following a previously described protocol with slight modifications.^48^ Animals were placed into the same individual test compartments described above for 40 min to habituate them to the test conditions. Then, mice were injected ip with CTX or saline. Compound **21** or its solvent was sc injected at 120 min after CTX ip administration, and pain behaviors were recorded for 2 min every 30 min in the period from 150 to 240 min. These pain-related behaviors were coded according to the following scale: 0 = normal, 1 = piloerection, 2 = labored breathing, 3 = licking of the abdomen, and 4 = stretching and contractions of the abdomen. At the end of the 2 h observation period (i.e., 4 h after the CTX injection), the sensory threshold in the abdomen was measured 240 min after CTX administration, using a series of von Frey filaments with bending forces ranging from 0.02 to 2 g (Stoelting, Wood Dale, USA). Testing was always initiated with the 0.4 g filament. The response to the filament was considered positive if immediate licking/scratching of the application site, sharp retraction of the abdomen, or jumping was observed. If there was a positive response, a weaker filament was used; if there was no response, a stronger stimulus was then selected. The 50% threshold withdrawal was determined using the up and down methods and calculated using the Up–Down Reader software.^67^

## Supporting Information Available

Complete details of in vitro biological methods, ^1^H and ^13^C NMR spectra and elemental analysis data of the new compounds, HPLC trace for compounds **15**, **19**, and **21**, non-covalent interactions between the inhibitors and the active site residues of sEH, analysis of active site water molecules in MD simulations, plots of the dihedral angle that describes the rotation of the benzohomoadamantane in the left-hand side pocket of the sEH active site along the MD simulations, cytochrome inhibition of compounds **15**, **21**, and **22**, and pharmacokinetic data of compounds **15** and **21** (PDF)
PDB files corresponding to the most populated cluster obtained from the MD simulations of compounds **15** (PDB)
PDB files corresponding to the most populated cluster obtained from the MD simulations of compounds **21** (PDB)
PDB files corresponding to the most populated cluster obtained from the MD simulations of compounds **13** (PDB)
PDB files corresponding to the most populated cluster obtained from the MD simulations of compounds **23** (PDB)
PDB files corresponding to the most populated cluster obtained from the MD simulations of compounds **24** (PDB)
Molecular movie of the spontaneous binding accelerated molecular dynamics simulation of compound **15** into the sEH active site (MPG)
Molecular formula string and data (CSV)

## Supplementary Material

jm2c00515_si_001.pdf

jm2c00515_si_002.pdb

jm2c00515_si_003.pdb

jm2c00515_si_004.pdb

jm2c00515_si_005.pdb

jm2c00515_si_006.pdb

jm2c00515_si_007.mpg

## Abbreviations Used

AA: arachidonic acid
aMD: accelerated molecular dynamics
ATR: attenuated total reflectance
BFC: benzyloxytrifluoromethylcoumarin
COX: cyclooxygenase
CYP: cytochrome P450
CTX: cyclophosphamide
DBF: dibenzylfluorescein
EETs: epoxyeicosatrienoic acids
EtOAc: ethyl acetate
LOX: lipoxygenase
MS-PPOH: *N*-methanesulfonyl-6-(2-proparyloxyphenyl)hexanamide
ND: not determined
PI: propidium iodide
sEH: soluble epoxide hydrolase
sEHI: soluble epoxide hydrolase inhibitors

## References

[ref1] Harizi H.; Corcuff J. B.; Gualde N. Arachidonic-acid-derived eicosanoids: roles in biology and immunopathology. Trends Mol. Med. 2008, 14, 461–469. 10.1016/j.molmed.2008.08.005. 18774339

[ref2] Hanna V. S.; Hafez E. A. A. Synopsis of arachidonic acid metabolism: a review. J. Adv. Res. 2018, 11, 23–32. 10.1016/j.jare.2018.03.005. 30034873 PMC6052663

[ref3] Funk C. D. Prostaglandins and leukotrienes: advances in eicosanoid biology. Science 2001, 294, 1871–1875. 10.1126/science.294.5548.1871. 11729303

[ref4] Meirer K.; Steinhilber D.; Proschak E. Inhibitors of the arachidonic acid cascade: interfering with multiple pathways. Basic Clin. Pharmacol. Toxicol. 2014, 114, 83–91. 10.1111/bcpt.12134. 24015667

[ref5] Rubin P.; Mollison K. W. Pharmacotherapy of diseases mediated by 5-lipooxygenase pathway eicosanoids. Prostaglandins Other Lipid Mediators 2007, 83, 188–197. 10.1016/j.prostaglandins.2007.01.005. 17481554

[ref6] Sinha S.; Doble M.; Manju S. L. 5-Lypoxygenase as a drug target: a review on trends in inhibitors structural design, SAR and mechanism based approach. Bioorg. Med. Chem. 2019, 27, 3745–3759. 10.1016/j.bmc.2019.06.040. 31331653

[ref7] Spector A. A.; Norris A. W. Action of epoxyeicosatrienoic acids on cellular function. Am. J. Physiol.: Cell Physiol. 2007, 292, C996–C1012. 10.1152/ajpcell.00402.2006. 16987999

[ref8] Kaspera R.; Totah R. A. Epoxyeicosatrienoic acids: formation, metabolism and potential role in tissue physiology and pathology. Expert Opin. Drug Metab. Toxicol. 2009, 5, 757–771. 10.1517/17425250902932923. 19505190

[ref9] Harris T. R.; Hammock B. D. Soluble epoxide hydrolase: gene structure, expression and deletion. Gene 2013, 526, 61–74. 10.1016/j.gene.2013.05.008. 23701967 PMC3733540

[ref10] Sun C.-P.; Zhang X.-Y.; Morisseau C.; Hwang S. H.; Zhang Z.-J.; Hammock B. D.; Ma X.-C. Discovery of soluble epoxide hydrolase inhibitors from chemical synthesis and natural products. J. Med. Chem. 2021, 64, 184–215. 10.1021/acs.jmedchem.0c01507. 33369424 PMC7942193

[ref11] Inceoglu B.; Bettaieb A.; Trindade da Silva C. A. T.; Lee K. S. S.; Haj F. G.; Hammock B. D. Endoplasmic reticulum stress in the peripheral nervous system is a significant driver of neuropathic pain. Proc. Natl. Acad. Sci. U.S.A. 2015, 112, 9082–9087. 10.1073/pnas.1510137112. 26150506 PMC4517273

[ref12] Kodani S. D.; Hammock B. D. The 2014 Bernard B. Brodie Award Lecture—Epoxide Hydrolases: drug metabolism to therapeutics for chronic pain. Drug Metab. Dispos. 2015, 43, 788–802. 10.1124/dmd.115.063339. 25762541 PMC4407705

[ref13] Pillarsetti S.; Khanna I. A multimodal disease modifying approach to treat neuropathic pain – inhibition of soluble epoxide hydrolase (sEH). Drug Discovery Today 2015, 20, 1382–1390. 10.1016/j.drudis.2015.07.017. 26259523

[ref14] Wagner K. M.; McReynolds C. B.; Schmidt W. K.; Hammock B. D. Soluble epoxide hydrolase as a therapeutic target for pain, inflammatory and neurodegenerative diseases. Pharmacol. Ther. 2017, 180, 62–76. 10.1016/j.pharmthera.2017.06.006. 28642117 PMC5677555

[ref15] Wagner K. M.; Gomes A.; McReynolds C. B.; Hammock B. D. Soluble epoxide hydrolase regulation of lípid mediators límits pain. Neurotherapeutics 2020, 17, 900–916. 10.1007/s13311-020-00916-4. 32875445 PMC7609775

[ref16] Wang Y.; Wagner K. M.; Morisseau C.; Hammock B. D. Inhibition of the soluble epoxide hydrolase as an analgesic strategy: a review of preclinical evidence. J. Pain Res. 2021, 14, 61–72. 10.2147/jpr.s241893. 33488116 PMC7814236

[ref17] Chen D.; Whitcomb R.; MacIntyre E.; Tran V.; Do Z. N.; Sabry J.; Patel D. V.; Anandan S. K.; Gless R.; Webb H. K. Phamacokinetics and pharmacodynamics of AR9281, an inhibitor of soluble epoxide hydrolase, in single- and multiple-dose studies in healthy human subjects. J. Clin. Pharmacol. 2012, 52, 319–328. 10.1177/0091270010397049. 21422238

[ref18] Hammock B. D.; McReynolds C. B.; Wagner K.; Buckpitt A.; Cortes-Puch I.; Croston G.; Lee K. S. S.; Yang J.; Schmidt W. K.; Hwang S. H. Movement to the clinic of soluble epoxide hydrolase inhibitor EC5026 as an analgesic for neuropathic pain and for use as a nonaddictive opioid alternative. J. Med. Chem. 2021, 64, 1856–1872. 10.1021/acs.jmedchem.0c01886. 33550801 PMC7917437

[ref19] Guedes A.; Galuppo L.; Hood D.; Hwang S. H.; Morisseau C.; Hammock B. D. Soluble epoxide hydrolase activity and pharmacologic inhibition in horses with chronic severe laminitis. Equine Vet. J. 2017, 49, 345–351. 10.1111/evj.12603. 27338788 PMC5580818

[ref20] Codony S.; Valverde E.; Leiva R.; Brea J.; Isabel Loza M. I.; Morisseau C.; Hammock B. D.; Vázquez S. Exploring the size of the lipophilic unit of the soluble epoxide hydrolase inhibitors. Bioorg. Med. Chem. 2019, 27, 115078 10.1016/j.bmc.2019.115078. 31488357 PMC6892585

[ref21] Codony S.; Calvó-Tusell C.; Valverde E.; Osuna S.; Morisseau C.; Loza M. I.; Brea J.; Pérez C.; Rodríguez-Franco M. I.; Pizarro-Delgado J.; Corpas R.; Griñán-Ferré C.; Pallàs M.; Sanfeliu C.; Vázquez-Carrera M.; Hammock B. D.; Feixas F.; Vázquez S. From the design to the in vivo evaluation of benzohomoadamantane-derived soluble epoxide hydrolase inhibitors for the treatment of acute pancreatitis. J. Med. Chem. 2021, 64, 5429–5446. 10.1021/acs.jmedchem.0c01601. 33945278 PMC8634379

[ref22] Martín-López J.; Codony S.; Bartra C.; Morisseau C.; Loza M. I.; Sanfeliu C.; Hammock B. D.; Brea J.; Vázquez S. 2-(Piperidin-4-yl)acetamides as potent inhibitors of soluble epoxide hydrolase with anti-inflammatory activity. Pharmaceuticals 2021, 14, 1323 10.3390/ph14121323. 34959721 PMC8703317

[ref23] Torres E.; Duque M. D.; López-Querol M.; Taylor M. C.; Naesens L.; Ma C.; Pinto L. H.; Sureda F. X.; Kelly J. M.; Vázquez S. Synthesis of benzopolycyclic cage amines: NMDA receptor antagonist, trypanocidal and antiviral activities. Bioorg. Med. Chem. 2012, 20, 942–948. 10.1016/j.bmc.2011.11.050. 22178660 PMC3353318

[ref24] Valverde E.; Sureda F. X.; Vázquez S. Novel benzopolycliclic amines with NMDA receptor antagonist activity. Bioorg. Med. Chem. 2014, 22, 2678–2683. 10.1016/j.bmc.2014.03.025. 24698811

[ref25] Barniol-Xicota M.; Escandell A.; Valverde E.; Julián E.; Torrents E.; Vázquez S. Antibacterial activity of novel benzopolycyclic amines. Bioorg. Med. Chem. 2015, 23, 290–296. 10.1016/j.bmc.2014.11.041. 25515953

[ref26] Codony S.; Galdeano C.; Leiva R.; Turcu A. L.; Valverde E.; Vázquez S. Polycyclic compounds as soluble epoxide hydrolase inhibitors. WO 2019243414 A1, 2019.

[ref27] Wan D.; Yang J.; McReynolds C. B.; Barnych B.; Wagner K. M.; Morisseau C.; Hwang S. H.; Sun J.; Blöcher R.; Hammock B. D. In vitro and in vivo metabolism of a potent inhibitor of soluble epoxide hydrolase, 1-(1-propionylpiperidin-4-yl)-3-(4-(trifluoromethoxy)phenyl)urea. Front. Pharmacol. 2019, 10, 464 10.3389/fphar.2019.00464. 31143115 PMC6520522

[ref28] Jones P. D.; Tsai H.-J.; Do Z. N.; Morisseau C.; Hammock B. D. Synthesis and SAR of conformationally restricted inhibitors of soluble epoxide hydrolase. Bioorg. Med. Chem. Lett. 2006, 16, 5212–5216. 10.1016/j.bmcl.2006.07.009. 16870439 PMC1904344

[ref29] Rose T. E.; Morisseau C.; Liu J.-Y.; Inceoglu B.; Jones P. D.; Sanborn J. R.; Hammock B. D. 1-aryl-3-(1-acylpiperidin-4-yl)urea inhibitors of human and murine soluble epoxide hydrolase: structure-activity relationships, pharmacokinetics, and reduction of inflammatory pain. J. Med. Chem. 2010, 53, 7067–7075. 10.1021/jm100691c. 20812725 PMC3285450

[ref30] Jones P. D.; Wolf N. M.; Morisseau C.; Whetstone P.; Hock B.; Hammock B. D. Fluorescent substrates for soluble epoxide hydrolase and application to inhibition studies. Anal. Biochem. 2005, 343, 66–75. 10.1016/j.ab.2005.03.041. 15963942 PMC1447601

[ref31] Öster L.; Tapani S.; Xue Y.; Käck H. Successful generation of structural information for fragment-based drug discovery. Drug Discovery Today 2015, 20, 1104–1111. 10.1016/j.drudis.2015.04.005. 25931264

[ref32] Calvó-Tusell C.; Maria-Solano M. A.; Osuna S.; Feixas F. Time evolution of millisecond allosteric activation of imidazole glycerol phosphate synthase. J. Am. Chem. Soc. 2022, 144, 7146–7159. 10.1021/jacs.1c12629. 35412310 PMC9052757

[ref33] Curado-Carballada C.; Feixas F.; Iglesias-Fernández J.; Osuna S. Hidden conformations in *Aspergillus niger* monoamine oxidase are key for catalytic efficiency. Angew. Chem., Int. Ed. 2019, 58, 3097–3101. 10.1002/anie.201812532. 30600584

[ref34] Daina A.; Michielin O.; Zoete V. SwissADME: a free web tool to evaluate pharmacokinetics, drug-likeness and medicinal chemistry friendliness of small molecules. Sci. Rep. 2017, 7, 42717 10.1038/srep42717. 28256516 PMC5335600

[ref35] Lagorce D.; Bouslama L.; Becot J.; Miteva M. A.; Villoutreix B. O. FAF-Drugs4: free ADME-tox filtering computations for chemical biology and early stages drug discovery. Bioinformatics 2017, 33, 3658–3660. 10.1093/bioinformatics/btx491. 28961788

[ref36] Di L.; Kerns E. H.; Fan K.; McConnell O. J.; Carter G. T. High throughput artificial membrane permeability assay for blood-brain barrier. Eur. J. Med. Chem. 2003, 38, 223–232. 10.1016/s0223-5234(03)00012-6. 12667689

[ref37] Hwang S. H.; Wecksler A. T.; Zhang G.; Morisseau C.; Nguyen L. V.; Fu S. H.; Hammock B. D. Synthesis and biological evaluation of sorafenib- and reforafenib-like sEH inhibitors. Bioorg. Med. Chem. Lett. 2013, 23, 3732–3737. 10.1016/j.bmcl.2013.05.011. 23726028 PMC3744640

[ref38] Wecksler A. T.; Hwang S. H.; Liu J.-Y.; Wettersten H. I.; Morisseau C.; Wu J.; Weiss R. H.; Hammock B. D. Biological evaluation of a novel sorafenib analogue, *t*-CUPM. Cancer Chemother. Pharmacol. 2015, 75, 161–171. 10.1007/s00280-014-2626-2. 25413440 PMC4400119

[ref39] Liang Z.; Zhang B.; Xu M.; Morisseau C.; Hwang S. H.; Hammock B. D.; Li Q. X. 1-Trifluoromethoxyphenyl-3-(1-propionylpiperidin-4-yl)urea, a selective and potent dual inhibitor of soluble epoxide hydrolase and p38 kinase intervenes in Alzheimer’s signaling in human nerve cells. ACS Chem. Neurosci. 2019, 10, 4018–4030. 10.1021/acschemneuro.9b00271. 31378059 PMC7028313

[ref40] Václavíková R.; Hughes D. J.; Souček P. Microsomal epoxide hydrolase 1 (EPHX1): gene, structure, function, and role in human disease. Gene 2015, 571, 1–8. 10.1016/j.gene.2015.07.071. 26216302 PMC4544754

[ref41] Baron R. Capsaicin and nociception: from basic mechanisms to novel drugs. Lancet 2000, 356, 785–787. 10.1016/s0140-6736(00)02649-0. 11022922

[ref42] Woolf C. J. Central sensitization: implications for the diagnosis and treatment of pain. Pain 2011, 152, S2–S15. 10.1016/j.pain.2010.09.030. 20961685 PMC3268359

[ref43] Miura M.; Sato I.; Kiyohara H.; Yokoyama K.; Koji T.; Terada H.; Yamaguchi T.; Amano Y. Cyclic amino compound, or salt thereof. JP 2011016743 A, 2011.

[ref44] Ma M.; Ren Q.; Fujita Y.; Ishima T.; Zhang J. C.; Hashimoto K. Effects of AS2586114, a soluble epoxide hydrolase inhibitor, on hyperlocomotion and prepulse inhibition deficits in mice after administration of phencyclidine. Pharmacol., Biochem. Behav. 2013, 110, 98–103. 10.1016/j.pbb.2013.06.005. 23792539

[ref45] Taguchi N.; Nakayama S.; Tanaka M. Single administration of soluble epoxide hydrolase inhibitor suppresses neuroinflammation and improves neuronal damage after cardiac arrest in mice. Neurosci. Res. 2016, 111, 56–63. 10.1016/j.neures.2016.05.002. 27184295

[ref46] Griñán-Ferré C.; Codony S.; Pujol E.; Yang J.; Leiva R.; Escolano C.; Puigoriol-Illamola D.; Companys-Alemany J.; Corpas R.; Sanfeliu C.; Pérez M. I.; Loza J.; Brea C.; Morisseau B. D.; Hammock S.; Vázquez M.; Pallàs C.; Galdeano C. Pharmacological inhibition of soluble epoxide hydrolase as a new therapy for Alzheimer’s disease. Neurotherapeutics 2020, 17, 1825–1835. 10.1007/s13311-020-00854-1. 32488482 PMC7851240

[ref47] Wang M.-H.; Brand-Schieber E.; Zand B. A.; Nguyen X.; Falck J. R.; Balu N.; Schwartzman M. L. Cytochrome P450-derived arachidonic acid metabolism in the rat kidney: characterization of selective inhibitors. J. Pharmacol. Exp. Ther. 1998, 284, 966–973. 9495856

[ref48] González-Cano R.; Artacho-Cordón A.; Romero L.; Tejada M. A.; Nieto F. R.; Merlos M.; Cañizares F. J.; Cendán C. M.; Fernández-Segura E.; Baeyens J. M. Urinary bladder sigma-1 receptors: a new target for cystitis treatment. Pharmacol. Res. 2020, 155, 104724 10.1016/j.phrs.2020.104724. 32105755

[ref49] Lai H. H.; Gardner V.; Ness T. J.; Gereau R. W. Segmental hyperalgesia to mechanical stimulus in interstitial cystitis/bladder pain syndrome: evidence of central sensitization. J. Urol. 2014, 191, 1294–1299. 10.1016/j.juro.2013.11.099. 24316091 PMC4070875

[ref50] Bon K.; Lichtensteiger C. A.; Wilson S. G.; Mogil J. S. Characterization of cyclophosphamide cystitis, a model of visceral and referred pain, in the mouse: species and strain differences. J. Urol. 2003, 170, 1008–1012. 10.1097/01.ju.0000079766.49550.94. 12913760

[ref51] Case D. A.; Ben-Shalom I. Y.; Brozell S. R.; Cerutti D. S.; Cheatham T. E. I.; Cruzeiro V. W. D.; Darden T. A.; Duke R. E.; Ghoreishi D.; Gilson M. K.; Gohlke H.; Goetz A. W.; Greene D.; Harris R.; Homeyer N.; Izadi S.; Kovalenko A.; Kurtzman T.; Lee T. S.; LeGrand S.; Li P.; Lin C.; Liu J.; Luchko T.; Luo R.; Mermelstein D. J.; Merz K. M.; Miao Y.; Monard G.; Nguyen C.; Nguyen H.; Omelyan I.; Onufriev A.; Pan F.; Qi R.; Roe D. R.; Roitberg A.; Sagui C.; Schott-Verdugo S.; Shen J.; Simmerling C. L.; Smith J.; Salomon-Ferrer R.; Swails J.; Walker R. C.; Wang J.; Wei H.; Wolf R. M.; Wu X.; Xiao L.; York D. M.; Kollman P. A. AMBER 2018; University of California: San Francisco, 2018.

[ref52] Wang J.; Wolf R. M.; Caldwell J. W.; Kollman P. A.; Case D. A. Development and testing of a general amber force field. J. Comput. Chem. 2004, 25, 1157–1174. 10.1002/jcc.20035. 15116359

[ref53] Bayly C. I.; Cieplak P.; Cornell W.; Kollman P. A. A well-behaved electrostatic potential based method using charge restraints for deriving atomic charges: the RESP model. J. Phys. Chem. 1993, 97, 10269–10280. 10.1021/j100142a004.

[ref54] Besler B. H.; Merz K. M. Jr.; Kollman P. A. Atomic charges derived from semiempirical methods. J. Comput. Chem. 1990, 11, 431–439. 10.1002/jcc.540110404.

[ref55] Singh U. C.; Kollman P. A. An approach to computing electrostatic charges for molecules. J. Comput. Chem. 1984, 5, 129–145. 10.1002/jcc.540050204.

[ref56] Frisch M. J.; Trucks G. W.; Schlegel H. B.; Scuseria G. E.; Robb M. A.; Cheeseman J. R.; Scalmani G.; Barone V.; Mennucci B.; Petersson G. A.; Nakatsuji H.; Caricato M.; Li X.; Hratchian H. P.; Izmaylov A. F.; Bloino J.; Zheng G.; Sonnenberg J. L.; Hada M.; Ehara M.; Toyota K.; Fukuda R.; Hasegawa J.; Ishida M.; Nakajima T.; Honda Y.; Kitao O.; Nakai H.; Vreven T.; Montgomery J. A. Jr.; Peralta J. E.; Ogliaro F.; Bearpark M.; Heyd J. J.; Brothers E.; Kudin K. N.; Staroverov V. N.; Kobayashi R.; Normand J.; Raghavachari K.; Rendell A.; Burant J. C.; Iyengar S. S.; Tomasi J.; Cossi M.; Rega N.; Millam J. M.; Klene M.; Knox J. E.; Cross J. B.; Bakken V.; Adamo C.; Jaramillo J.; Gomperts R.; Stratmann R. E.; Yazyev O.; Austin A. J.; Cammi R.; Pomelli C.; Ochterski J. W.; Martin R. L.; Morokuma K.; Zakrzewski V. G.; Voth G. A.; Salvador P.; Dannenberg J. J.; Dapprich S.; Daniels A. D.; Farkas Ö.; Foresman J. B.; Ortiz J. V.; Cioslowski J.; Fox D. J. Gaussian 09, Revision A.02; Gaussian, Inc.: Pittsburgh, PA, 2009.

[ref57] Grosdidier A.; Zoete V.; Michielin O. SwissDock a protein-small molecule docking web service based on EADock DSS. Nucleic Acids Res. 2011, 39, W270–W277. 10.1093/nar/gkr366. 21624888 PMC3125772

[ref58] Grosdidier A.; Zoete V.; Michielin O. Fast docking using the CHARMM force field with EADock DSS. J. Comput. Chem. 2011, 32, 2149–2159. 10.1002/jcc.21797. 21541955

[ref59] Maier J. A.; Martinez C.; Kasavajhala K.; Wickstrom L.; Hauser K. E.; Simmerling C. ff14SB: improving the accuracy of protein side chain and backbone parameters from ff99SB. J. Chem. Theory Comput. 2015, 11, 3696–3713. 10.1021/acs.jctc.5b00255. 26574453 PMC4821407

[ref60] Sagui C.; Darden T. A. Molecular dynamics simulations of biomolecules: long-range electrostatic effects. Annu. Rev. Biophys. Biomol. Struct. 1999, 28, 155–179. 10.1146/annurev.biophys.28.1.155. 10410799

[ref61] Ryckaert J.-P.; Ciccotti G.; Berendsen H. J. C. Numerical integration of the cartesian equations of motion of a system with constraints: molecular dynamics of *n*-alkanes. J. Comput. Phys. 1977, 23, 327–341. 10.1016/0021-9991(77)90098-5.

[ref62] Wu X.; Brooks B. R. Self-guided Langevin dynamics simulation method. Chem. Phys. Lett. 2003, 381, 512–518. 10.1016/j.cplett.2003.10.013.

[ref63] Roe D. R.; Cheatham T. E. III PTRAJ and CPPTRAJ: software for processing and analysis of molecular dynamics trajectory data. J. Chem. Theory Comput. 2013, 9, 3084–3095. 10.1021/ct400341p. 26583988

[ref64] Hamelberg D.; Mongan J.; McCammon J. A. Accelerated molecular dynamics: a promising and efficient simulation method for biomolecules. J. Chem. Phys. 2004, 120, 11919 10.1063/1.1755656. 15268227

[ref65] Hamelberg D.; de Oliveira C. A. F.; McCammon J. A. Sampling of slow diffusive conformational transitions with accelerated molecular dynamics. J. Chem. Phys. 2007, 127, 155102 10.1063/1.2789432. 17949218

[ref66] Entrena J. M.; Cobos E. J.; Nieto F. R.; Cendán C. M.; Gris G.; Del Pozo E.; Zamanillo D.; Baeyens J. M. Sigma-1 receptors are essential for capsaicin-induced mechanical hypersensitivity: studies with selective sigma-1 ligands and sigma-1 knockout mice. Pain 2009, 143, 252–261. 10.1016/j.pain.2009.03.011. 19375855

[ref67] Gonzalez-Cano R.; Boivin B.; Bullock D.; Cornelissen L.; Andrews N.; Costigan M. Up-down reader: an open source program for efficiently processing 50% von Frey thresholds. Front. Pharmacol. 2018, 9, 433 10.3389/fphar.2018.00433. 29765323 PMC5938897

