# Supplementary material for: Synthesis, In Vitro Profiling, and In Vivo Evaluation of Benzohomoadamantane-Based Ureas for Visceral Pain: A New Indication for Soluble Epoxide Hydrolase Inhibitors
Source: J Med Chem. 2022 Oct 12;65(20):13660–80. doi: 10.1021/acs.jmedchem.2c00515 (PMC9620236; doi:10.1021/acs.jmedchem.2c00515)
Supplement: Supplementary file 1 — jm2c00515_si_001.pdf [file jm2c00515_si_001.pdf]

Supporting Information

Synthesis, *in vitro* profiling and *in vivo* evaluation of benzohomoadamantane-based ureas for visceral pain: a new indication for soluble epoxide hydrolase inhibitors

Sandra Codony[1], José M. Entrena[2], Carla Calvó-Tusell[3], Beatrice Jora[1], Rafael González-Cano[4], Sílvia Osuna[3,5], Rubén Corpas[6], Christophe Morisseau[7], Belén Pérez[8], Marta Barniol-Xicota[9], Christian Griñán-Ferré[10], Concepción Pérez[11], María Isabel Rodríguez-Franco[11], Antón L. Martínez[12], M. Isabel Loza[12], Mercè Pallàs[10], Steven H. L. Verhelst[11,13], Coral Sanfeliu[6], Ferran Feixas[3], Bruce D. Hammock[7], José Brea[12], Enrique J. Cobos[4], Santiago Vázquez[1]*

[1]Laboratori de Química Farmacèutica (Unitat Associada al CSIC), Facultat de Farmàcia i Ciències de l'Alimentació, and Institute of Biomedicine (IBUB), Universitat de Barcelona, Av. Joan XXIII, 27-31, 08028 Barcelona, Spain.

[2] Animal Behavior Research Unit, Scientific Instrumentation Center, University of Granada, Parque Tecnológico de Ciencias de la Salud, Armilla, 18100 Granada, Spain.

[3]CompBioLab Group, Departament de Química and Institut de Química Computacional i Catàlisi (IQCC), Universitat de Girona, C/ Maria Aurèlia Capmany 69, 17003 Girona, Spain.

[4]Department of Pharmacology, Faculty of Medicine and Biomedical Research Center (Neurosciences Institute), University of Granada. Biosanitary Research Institute ibs.GRANADA, Avenida de la Investigación 11, 18016, Granada, Spain.

[5]Institució Catalana de Recerca i Estudis Avançats (ICREA), 08010 Barcelona, Spain.

[6]Institute of Biomedical Research of Barcelona (IIBB), CSIC and IDIBAPS, 08036 Barcelona, Spain.

[7]Department of Entomology and Nematology and Comprehensive Cancer Center, University of California, Davis, CA 95616, USA.

[8]Department of Pharmacology, Therapeutics and Toxicology, Institute of Neurosciences, Autonomous University of Barcelona, 08193 Bellaterra, Barcelona, Spain.

[9]KU Leuven – University of Leuven, Laboratory of Chemical Biology, Department of Cellular and Molecular Medicine, Herestraat 49 box B901, 3000 Leuven, Belgium.

[10]Pharmacology Section, Department of Pharmacology, Toxicology and Therapeutic Chemistry, Faculty of Pharmacy and Food Sciences, Institute of Neuroscience, University of Barcelona (NeuroUB), Av. Joan XXIII 27-31, 08028 Barcelona, Spain.

[11]Institute of Medicinal Chemistry, Spanish National Research Council (CSIC), C/Juan de la Cierva 3, 28006 Madrid, Spain.

[12]Drug Screening Platform/Biofarma Research Group, CIMUS Research Center. University of Santiago de Compostela (USC), 15782 Santiago de Compostela, Spain.

[13]Leibniz Institute for Analytical Sciences ISAS, AG Chemical Proteomics, Otto-Hahn-Str. 6b, 44227 Dortmund, Germany.

* Corresponding author. Tel.: +34 934024533; *E-mail address:* svazquez@ub.edu *(S. Vázquez).*

Table of contents

In vitro biological methods Page S5

References Page S10

^1H and ^{13}C NMR spectra of compound **9** Page S11

^1H and ^{13}C NMR spectra of compound **10** Page S12

^1H and ^{13}C NMR spectra of compound **11** Page S13

^1H and ^{13}C NMR spectra of compound **12** Page S14

^1H and ^{13}C NMR spectra of compound **13** Page S15

^1H and ^{13}C NMR spectra of compound **14** Page S16

^1H and ^{13}C NMR spectra of compound **15** Page S17

^1H and ^{13}C NMR spectra of compound **16** Page S18

^1H and ^{13}C NMR spectra of compound **17** Page S19

^1H and ^{13}C NMR spectra of compound **18** Page S20

^1H and ^{13}C NMR spectra of compound **19** Page S21

^1H and ^{13}C NMR spectra of compound **20** Page S22

^1H and ^{13}C NMR spectra of compound **21** Page S23

^1H and ^{13}C NMR spectra of compound **22** Page S24

^1H and ^{13}C NMR spectra of compound **23** Page S25

^1H and ^{13}C NMR spectra of compound **24** Page S26

^1H and ^{13}C NMR spectra of compound **25** Page S27

^1H and ^{13}C NMR spectra of compound **28** Page S28

Table S1. Elemental analysis data. Page S29

HPLC blank Page S30

HPLC trace for compound **15** Page S30

HPLC trace for compound **19** Page S31

HPLC trace for compound **21** Page S31

Table S2. Values of MMGBSA calculations. Page S32

Table S3. Inhibition of recombinant human cytochromes P450 enzymes. Page S32

Table S4. Concentrations of compound **15** in mouse plasma at different times after subcutaneous administration at 5 mg/Kg. Page S33

Table S5. Concentrations of compound **21** in mouse plasma at different times after subcutaneous administration at 5 mg/Kg. Page S33

Figure S1. Spontaneous binding accelerated molecular dynamics simulation (aMD) of compound **15**. Page S34
Figure S2. Binding pose and molecular interactions of compound **15**. Page S35
Figure S3. Binding pose and molecular interactions of compound **21**. Page S36
Figure S4. Binding pose and molecular interactions of compounds **13** and **23** Page S38
Figure S5. Rotation and orientation of the benzohomoadamantane moiety in the LHS pocket of sEH. Page S40
Figure S6. Water occupation in the sEH active site. Page S41
Figure S7. Target spectrum of probe **28** in HEK293T cells Page S42
Figure S8. Minimal labeling concentration of **28** determination Page S42
Figure S9. Pull down experiments to confirm off-targets at 10 μM Page S43
Figure S10. Pull down experiments to confirm off-targets at 1 μM Page S44
Figure S11. Coomassie-stained gel of Figure 5. Page S44
Figure S12. Plasma concentration *vs* time for compound **15**. Page S45
Figure S13. Plasma concentration *vs* time for compound **21**. Page S45
Movie S1. Molecular movie of the simulation of **15** into the sEH. Page S45

Enzymatic sEH activity assay. The assay was performed as previously described.[1] All hsEH, msEH and rsEH IC_{50} values were determined by a fluorescence-based assay system in a 96-well format. Nonfluorescent MNPC cyano(6-methoxy-naphthalen-2-yl)methyl *trans*-[(3-phenyloxiran-2- yl)methyl] carbonate was used as the assay substrate at a concentration of 5 μM. This substrate is hydrolyzed by the sEH to the fluorescent 6-methoxynaphthaldehyde. The formation of the product was measured (λem = 330 nm, λex = 465 nm) by a Molecular Device M-2 plate reader. All measurements were performed in triplicate.

Microsomal stability. The human and mice pooled recombinant microsomes employed were purchased from Tebu-Xenotech. The compound was incubated at 37 ºC with the microsomes in a 50 mM phosphate buffer (pH = 7.4) containing 3 mM $MgCl_2$, 1 mM NADP, 10 mM glucose-6-phosphate and 1 U/mL glucose-6-phosphate-dehydrogenase. Samples (75 μL) were taken from each well at 0, 10, 20, 40 and 60 min and transferred to a plate containing 4 °C 75 μL acetonitrile and 30 μL of 0.5% formic acid in water were added for improving the chromatographic conditions. The plate was centrifuged (46000 g, 30 min) and supernatants were taken and analyzed in a UPLC-MS/MS (Xevo-TQD, Waters) by employing a BEH C18 column and an isocratic gradient of 0.1% formic acid in water: 0.1% formic acid acetonitrile (60:40). The metabolic stability of the compounds was calculated from the logarithm of the remaining compounds at each of the time points studied.

Solubility. A 10 mM stock solution of the compound was serially diluted in 100% DMSO and 1 μL of this solution was added to a 384-well UV-transparent plate (Greiner) containing 99 μL of PBS. The plate was incubated at 37 °C for 2 h and the light scattering was measured in a Nephelostar Plus reader (BMG LABTECH). The data were fitted to a segmented linear regression for measuring the compound solubility.

Parallel Artificial Membrane Permeation Assays - Blood-Brain Barrier (PAMPA-BBB). To evaluate the brain penetration of the different compounds, a parallel artificial membrane permeation assay for blood-brain barrier was used, following the method described by Di *et al.*[2] The *in vitro* permeability (Pe) of fourteen commercial drugs through lipid extract of porcine brain membrane together with the test compounds were determined. Commercial drugs and assayed compounds were tested using a mixture of PBS:EtOH (70:30). Assay validation was made by comparing the experimental permeability with the reported values of the commercial drugs by bibliography and lineal correlation between experimental and reported permeability of the fourteen commercial drugs using the parallel artificial membrane permeation assay was evaluated ($y = 1.5219x - 0.9129$; $R^2 = 0.9387$). From this equation, and taking into account the limits established by Di *et al.*[2] for BBB permeation, we established the ranges of permeability as compounds of high BBB permeation (CNS+): Pe (10-6 cm^{s-1}) > 5.149; compounds of low BBB permeation (CNS-): Pe (10-6 cm^{s-1}) < 2.131 and compounds of uncertain BBB permeation (CNS+/-): 5.149 > Pe (10-6 cm^{s-1}) > 2.131.

Cytotoxicity in SH-SY5Y cells. Cytotoxicity was evaluated in the human neuroblastoma SH-SY5Y cell line (ATCC Number: CRL-2266). Cells were cultured in Minimum Essential Medium / Ham's-F12 (1:1, v/v) medium, supplemented with non-essential amino acids, 10% fetal bovine serum, 1 mM glutamine and 50 µg/ml gentamycin (all reagents from Gibco, Invitrogen). For experiments, cells were seeded at 3×105 cells/ml (100 µl/well) in 96-well plates (Nunc). After 24 h, the testing compounds were added concentrate to triplicate wells to obtain the final different concentrations up to 100 µM. Compounds were incubated for further 24 h. At termination, cytotoxicity was analysed by the propidium iodide (PI) fluorescence stain assay and the 3-(4,5-dimethylthiazol-2-

yl)-2,5-diphenyl tetrazolium bromide (MTT) colorimetric assay. All compounds were tested in three independent experiments using different cell passages.

The PI assay measures cell death. PI enters into the cells with damaged membranes and greatly increases the fluorescence by binding to DNA. PI reagent (Molecular Probes) at the final concentration of 7.5 μg/ml was added to the cells and incubated for 1 h. The resulting fluorescence was measured by a Gemini XPS Microplate reader (Millipore) at 530 nm excitation and 645 nm emission. Percentage of cell death induced by the treatments was calculated from to the fluorescence of treated cells (Ft) relative to that of control cells (Fmin) and cells incubated with Triton X100 (Fmax) as the 0% and 100% cell death, respectively [% = ((Ft-Fmin)/(Fmax-Fmin)) x 100].

The MTT assay quantifies cellular metabolic activity as an indicator of cell viability and proliferation. MTT is a tretrazolium salt that when oxidised by metabolically active cells gives blue formazan crystals, that may be solubilized by a combination of a detergent (SDS) and an organic solvent (dimethylformamide) (all Sigma reagents). MTT was added to cultured cells at the final concentration of 0.5 mg/ml and incubated for 2 h. Then the solubilizing buffer was added to the wells and the culture plates were wrapped with Parafilm to avoid evaporation and maintained at 37°C overnight. The resulting colorimetric reaction was measured by a Multiskan Spectrum Spectrophotometer (Thermo) at 570 nm and a reference 630 nm wavelength. Results were given as a percentage of control cells values.

Cytochrome P450 inhibition assay. The objective of this study was to screen the inhibition potential of the compound using recombinant human cytochrome P450 enzymes (CYP1A2, CYP2C9, CYP2C19, CYP2D6, CYP3A4 (BFC) and CYP3A4 (DBF)) and probe substrates with fluorescent detection. Incubations were conducted in a 200 µL volume in 96-well microtiter plates (COSTAR 3915). The addition of the mixture

buffer-cofactor (KH_2PO_4 buffer, 1.3 mM NADP, 3.3 mM $MgCl_2$, 3.3 mM glucose-6-phosphate and 0.4 U/mL glucose-6-phosphate dehydrogenase), control supersomes, standard inhibitors (furafyline, tranylzypromine, ketoconazole, sulfaphenazole and quinidine; Sigma Aldrich), and previously diluted compound to plates was carried out by a liquid handling station (Zephyr Caliper). The plate was then preincubated at 37 °C for 5 min, and the reaction was initiated by the addition of prewarmed enzyme/substrate (E/S) mix. The E/S mix contained buffer (KH_2PO_4), c-DNA-expressed P450 in insect cell microsomes, substrate (3-cyano-7-ethoxycoumarin, for CYP1A2 and CYP2C19; 7-methoxy-4-(trifluoromethyl)coumarin for CYP2C9; 3-[2-(N,N-diethyl-N-methylammonium)ethyl]-7-methoxy-4-methylcoumarin for CYP2D6; and 7-benzyloxytrifluoromethyl coumarin (7-BFC) and dibenzylfluorescein (DBF) for CYP3A4) in a reaction volume of 200 µL. Reactions were terminated after various times (a specific time for each cytochrome) by addition of STOP solution (ACN/TrisHCl 0.5 M 80:20 or 2 N NaOH). Fluorescence per well was measured using a fluorescence plate reader (Tecan Infinity M1000 pro) and percentage of inhibition was calculated.

Permeability. The Caco-2 cells were cultured to confluency, trypsinized and seeded onto a filter transwell inserted at a density of ~10,000 cells/well in DMEM cell culture medium. Confluent Caco-2 cells were sub-cultured at passages 58-62 and grown in a humidified atmosphere of 5% CO_2 at 37ºC. Following an overnight attachment period (24 h after seeding), the cell medium was replaced with fresh medium in both the apical and basolateral compartments every other day. The cell monolayers were used for transport studies 21 days post seeding. The monolayer integrity was checked by measuring the transepithelial electrical resistance (TEER) obtaining values ≥ 500 Ω/cm^2. On the day of the study, after the TEER measurement, the medium was removed and the cells were washed twice with pre-warmed (37ºC) Hank's Balanced Salt Solution (HBSS) buffer to

remove traces of medium. Stock solutions were made in dimethyl sulfoxide (DMSO), and further diluted in HBSS (final DMSO concentration 1%). Each compound and reference compounds (Colchicine, E3S) were all tested at a final concentration of 10 µM. For A → B directional transport, the donor working solution was added to the apical (A) compartment and the transport media as receiver working solution was added to the basolateral (B) compartment. For B → A directional transport, the donor working was added to the basolateral (B) compartment and transport media as receiver working solution was added to the apical (A) compartment. The cells were incubated at 37ºC for 2 hours with gentle stirring.

At the end of the incubation, samples were taken from both donor and receiver compartments and transferred into 384-well plates and analyzed by UPLC-MS/MS. The detection was performed using an ACQUITY UPLC /Xevo TQD System. After the assay, Lucifer yellow was used to further validate the cell monolayer integrity, cells were incubated with LY 10µM in HBSS for 1hour at 37ºC, obtaining permeability (Papp) values for LY of ≤ 10 nm/s confirming the well-established Caco-2 monolayer.

hERG inhibition assay: The assay was carried out at a CHO cell line transfected with the hERG potassium channel. 72h before the assay, 2500 cells were seeded on a 384 well black plate (Greiner 781091). Cell line were maintained at 37ºC in a 5% CO_2 atmosphere for 24h and at 30ºC in a 5% CO_2 atmosphere for 48h plus. hERG activity was measured by using the FluxorTM Potassium Ion Chanel Assay Kit (Thermo Fisher F10016). Medium was replaced for 20µl Loading Buffer and the cells were incubated for 60 minutes at RT, protected from direct light. After incubation, Loading Buffer was replaced for Assay buffer and the compounds were incubated for 30 minutes at RT. 5µl of Stimulus Buffer was added to each well and the fluorescence was read (λ_{ex}=490 nm, λ_{em}=525nm)

using imaging plate reader system (FDSS7000EX, Hamamatsu®) every second after the establishment of a baseline line.

Inhibition of human lipoxygenase-5 (*h*LOX-5). AA and 2',7'-dichlorodihydrofluorescein diacetate (H_2DCFDA) were obtained from Sigma**.** Human recombinant LOX-5 was purchased from Cayman Chemical. For the determination of *h*LOX-5 activity, the method described by Pufahl *et al.* was followed.[3] The assay solution consisted of 50 mM Tris (pH 7.5), 2 mM EDTA, 2 mM $CaCl_2$, 3 μM AA, 10 μM ATP, 10 μM H_2DCFDA and 100 mU/well *h*LOX-5. For the enzyme inhibition studies the compounds to be tested were added to the assay solution prior to AA and ATP and were preincubated for a period of 10 min at room temperature, after which AA and ATP were added. The enzymatic reaction was carried out for 20 min and terminated by the addition of 40 μL of acetonitrile. The fluorescence measurement, 485 nm excitation and 520 nm emission, was performed on a FLUOstar OPTIMA (BMG LABTECH, Offenburg, Germany.). The IC_{50} is defined as the concentration of compound that inhibits enzymatic activity by 50% over the untreated enzyme control.

References

1. Jones, P. D.; Wolf, N. M.; Morisseau, C.; Whetstone, P.; Hock, B.; Hammock, B. D. Fluorescent substrates for soluble epoxide hydrolase and application to inhibition studies. *Anal. Biochem.* **2005**, *343*, 66–75.

2. Di, L.; Kerns, E. H.; Fan, K.; McConnell, O. J.; Carter, G. T. High throughput artificial membrane permeability assay for blood-brain barrier. *Eur. J. Med. Chem.* **2003**, *38*, 223–232.

3. Pufahl, R. A.; Kasten, T. P.; Hills, R.; Gierse, J. K.; Reitz, B. A.; Weinberg, R. A.; Masferrer, J. L. Development of a fluorescence-based enzyme assay of human 5-lipoxygenase. *Anal. Biochem.* **2007**, *364*, 204–212.

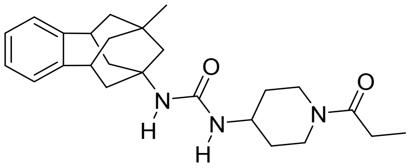

O
N
H
N
H
N
O

9

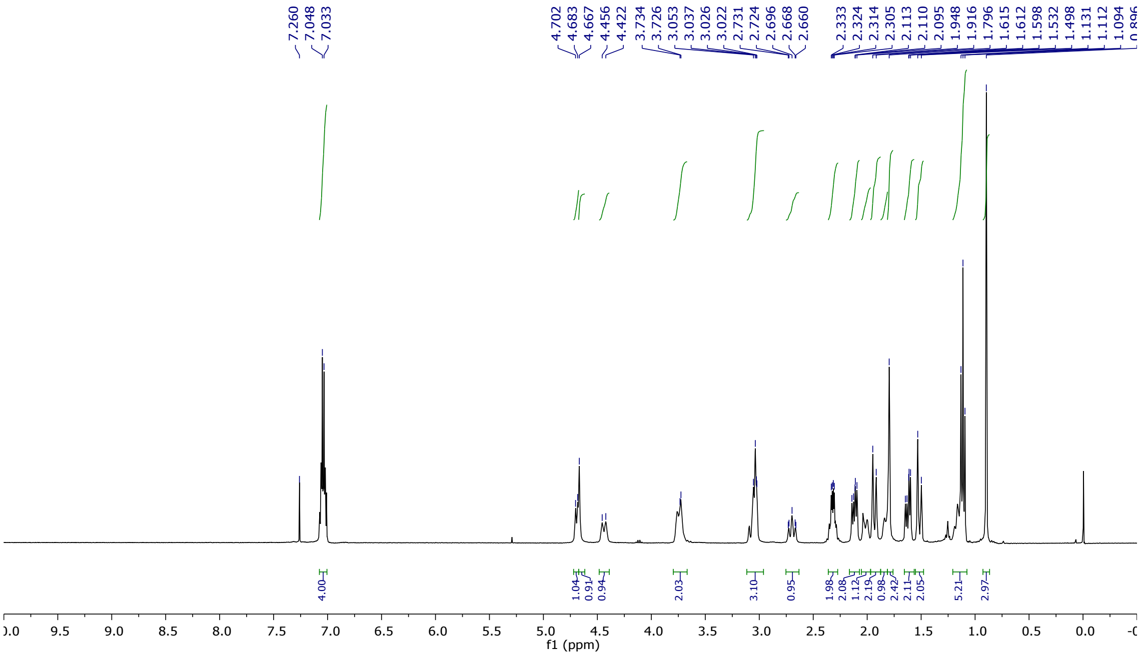

7.260
7.048
7.033
4.702
4.683
4.667
4.456
4.422
3.734
3.726
3.053
3.037
3.026
3.022
2.731
2.724
2.696
2.668
2.660
2.333
2.324
2.314
2.305
2.113
2.110
2.095
1.948
1.916
1.796
1.615
1.612
1.598
1.532
1.498
1.131
1.112
1.094
4.00
1.04
0.91
0.94
2.03
3.10
0.95
1.98
2.08
1.12
2.19
0.98
2.42
2.11
2.05
5.21
2.97
9.5
9.0
8.5
8.0
7.5
7.0
6.5
6.0
5.5
5.0
4.5
4.0
3.5
3.0
2.5
2.0
1.5
1.0
0.5
0.0
f1 (ppm)

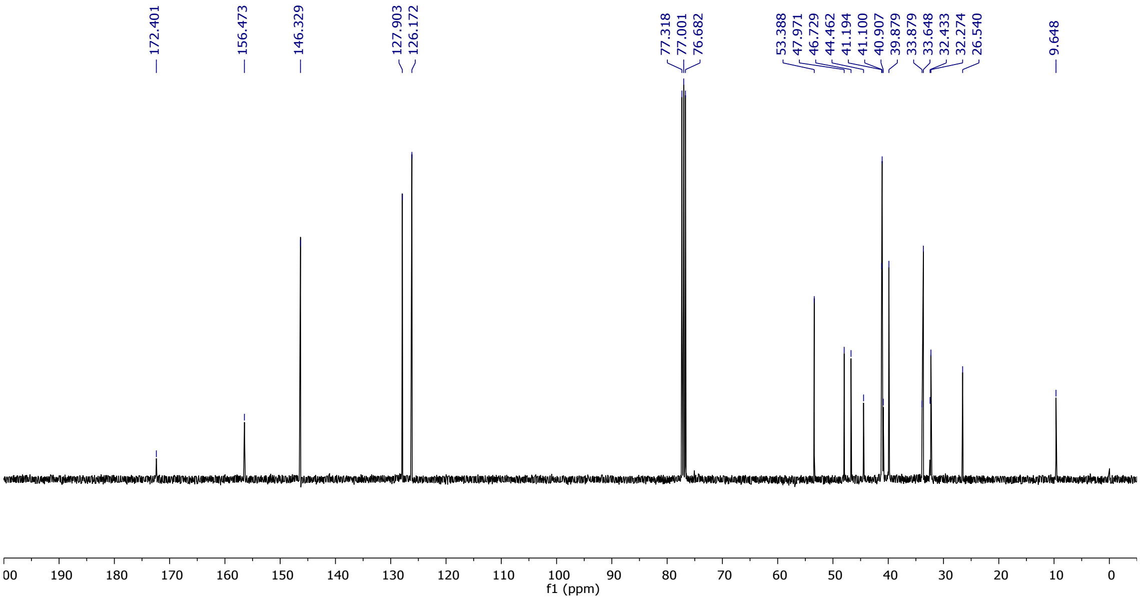

172.401
156.473
146.329
127.903
126.172
77.318
77.001
76.682
53.388
47.971
46.729
44.462
41.194
41.100
40.907
39.879
33.879
33.648
32.433
32.274
26.540
9.648
190
180
170
160
150
140
130
120
110
100
90
80
70
60
50
40
30
20
10
0
f1 (ppm)

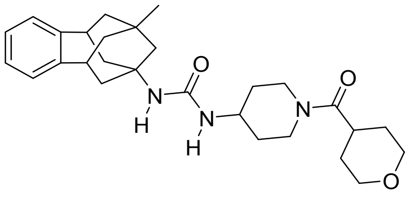

O
N
H
N
H
N
O
O

10

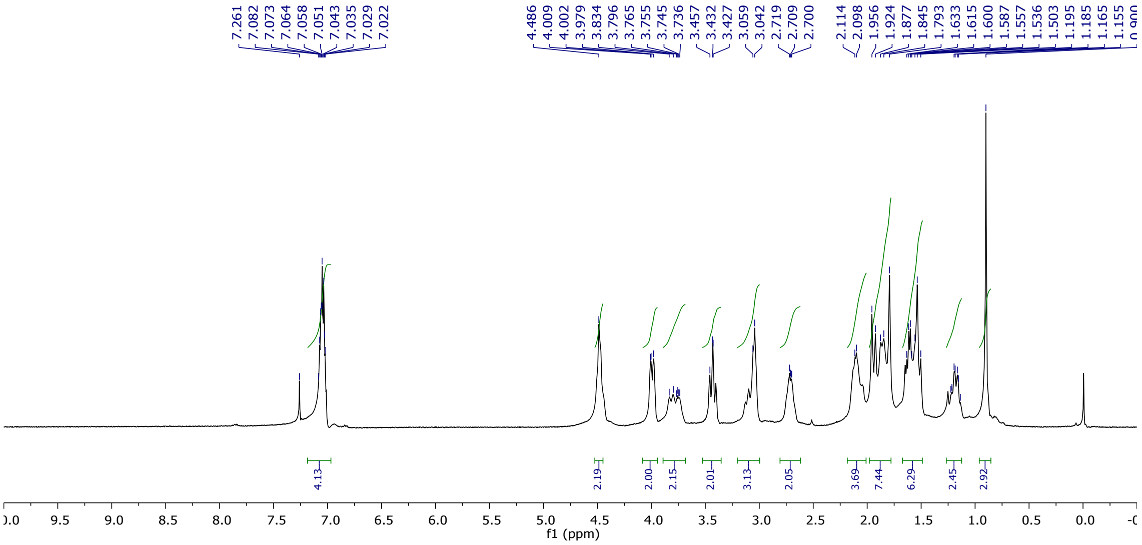

7.261
7.082
7.073
7.064
7.058
7.051
7.043
7.035
7.029
7.022
4.486
4.009
4.002
3.979
3.834
3.796
3.765
3.755
3.745
3.736
3.457
3.432
3.427
3.059
3.042
2.719
2.709
2.700
2.114
2.098
1.956
1.924
1.877
1.845
1.793
1.633
1.615
1.600
1.587
1.557
1.536
1.503
1.195
1.185
1.165
1.155
4.13
2.19
2.00
2.15
2.01
3.13
2.05
3.69
7.44
6.29
2.45
2.92
).0
9.5
9.0
8.5
8.0
7.5
7.0
6.5
6.0
5.5
5.0
4.5
4.0
3.5
3.0
2.5
2.0
1.5
1.0
0.5
0.0
-(
f1 (ppm)

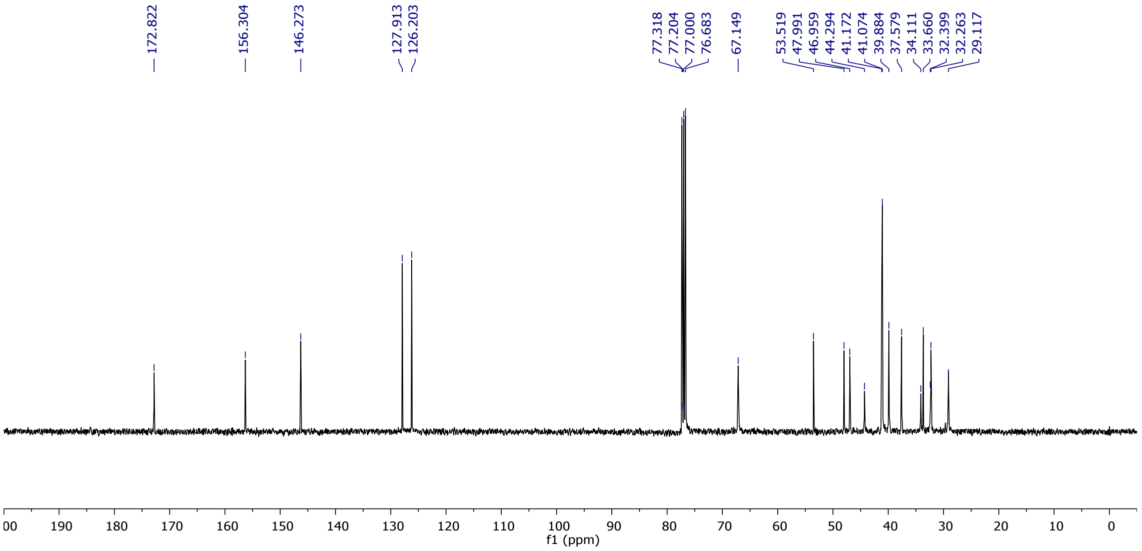

172.822
156.304
146.273
127.913
126.203
77.318
77.204
77.000
76.683
67.149
53.519
47.991
46.959
44.294
41.172
41.074
39.884
37.579
34.111
33.660
32.399
32.263
29.117
00
190
180
170
160
150
140
130
120
110
100
90
80
70
60
50
40
30
20
10
0
f1 (ppm)

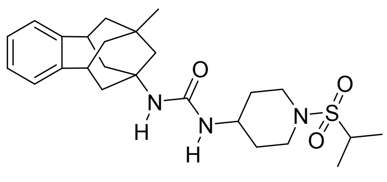

11

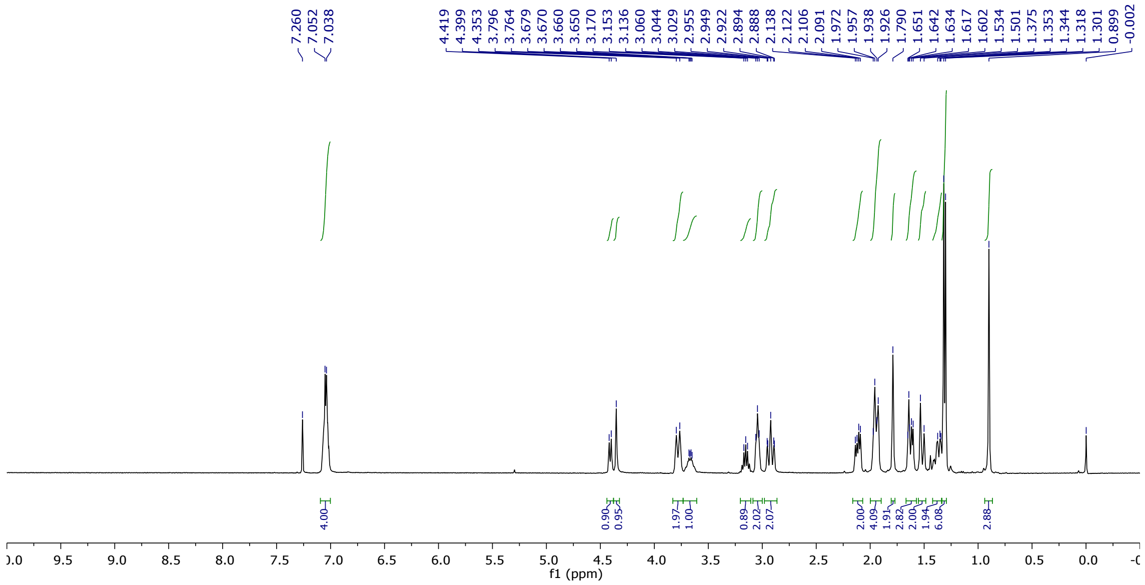

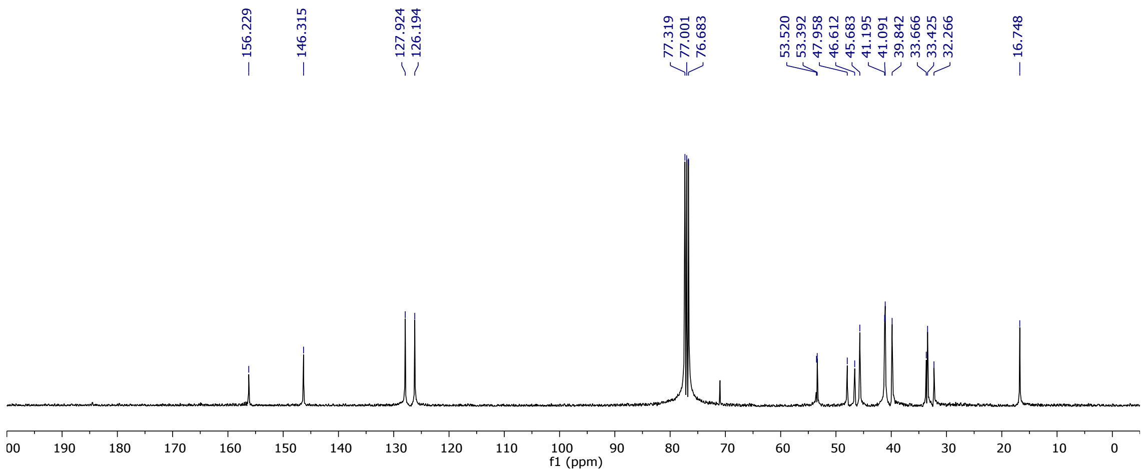

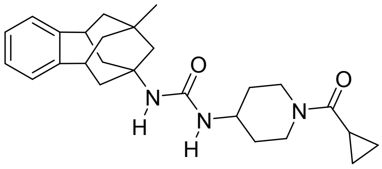

12

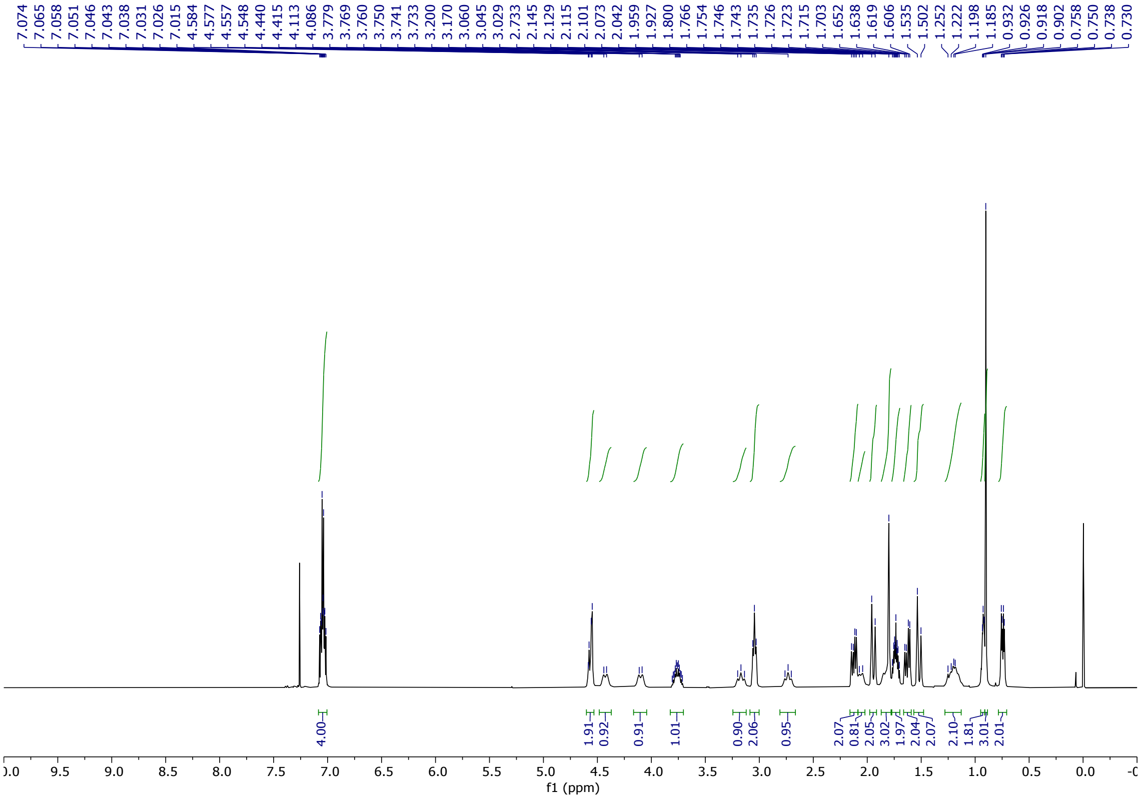
7.074 7.065 7.058 7.051 7.046 7.043 7.038 7.031 7.026 7.015 4.584 4.577 4.557 4.548 4.440 4.415 4.113 4.086 3.779 3.769 3.760 3.750 3.741 3.733 3.200 3.170 3.060 3.045 3.029 2.733 2.145 2.129 2.115 2.101 2.073 2.042 1.959 1.927 1.800 1.766 1.754 1.746 1.743 1.735 1.726 1.723 1.715 1.703 1.652 1.638 1.619 1.606 1.535 1.502 1.252 1.222 1.198 1.185 0.932 0.926 0.918 0.902 0.758 0.750 0.738 0.730
4.00
1.91 0.92 0.91 1.01 0.90 2.06 0.95
2.07 0.81 2.05 3.02 1.97 2.04 2.07 2.10 1.81 3.01 2.01
f1 (ppm)

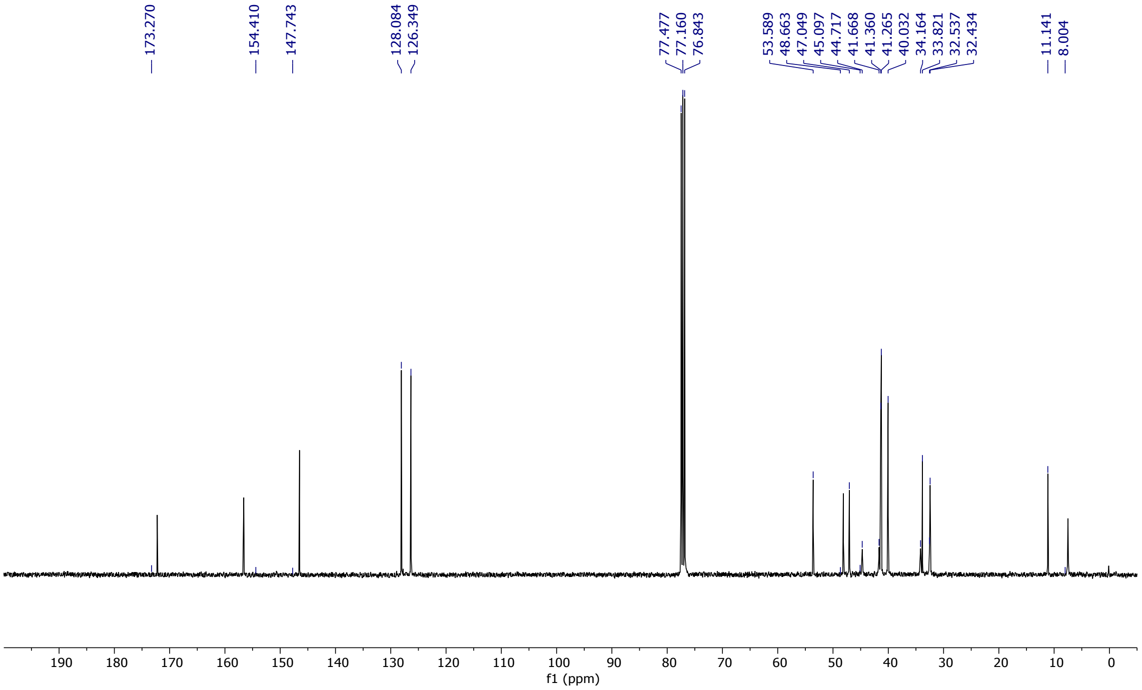
173.270
154.410
147.743
128.084 126.349
77.477 77.160 76.843
53.589 48.663 47.049 45.097 44.717 41.668 41.360 41.265 40.032 34.164 33.821 32.537 32.434
11.141 8.004
f1 (ppm)

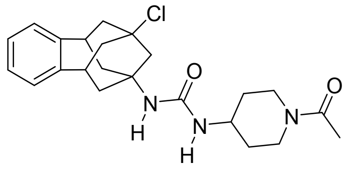

13

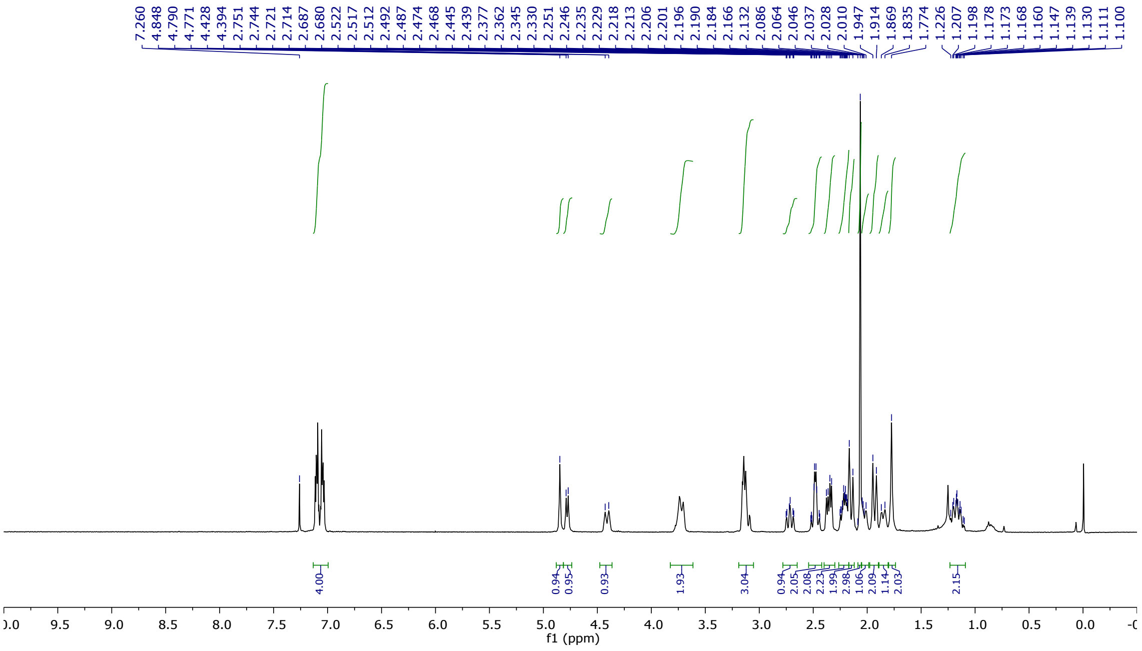

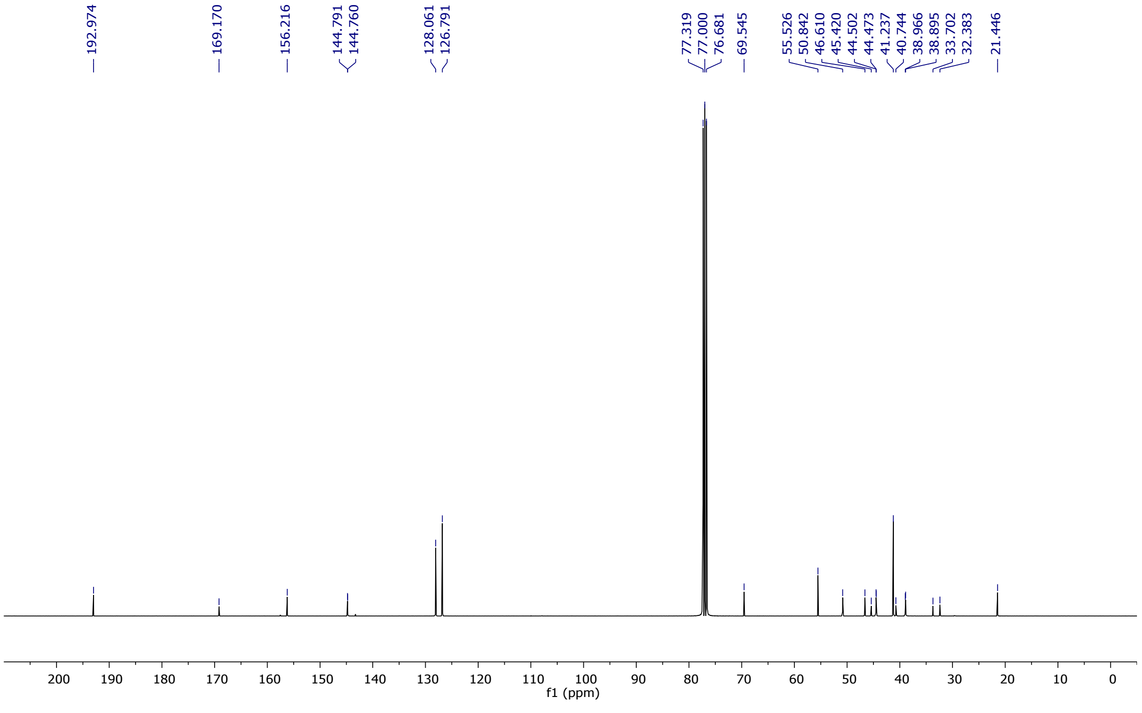

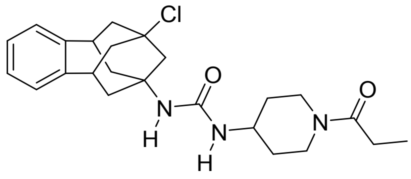
Cl
O
N
H
N
H
N
O

14

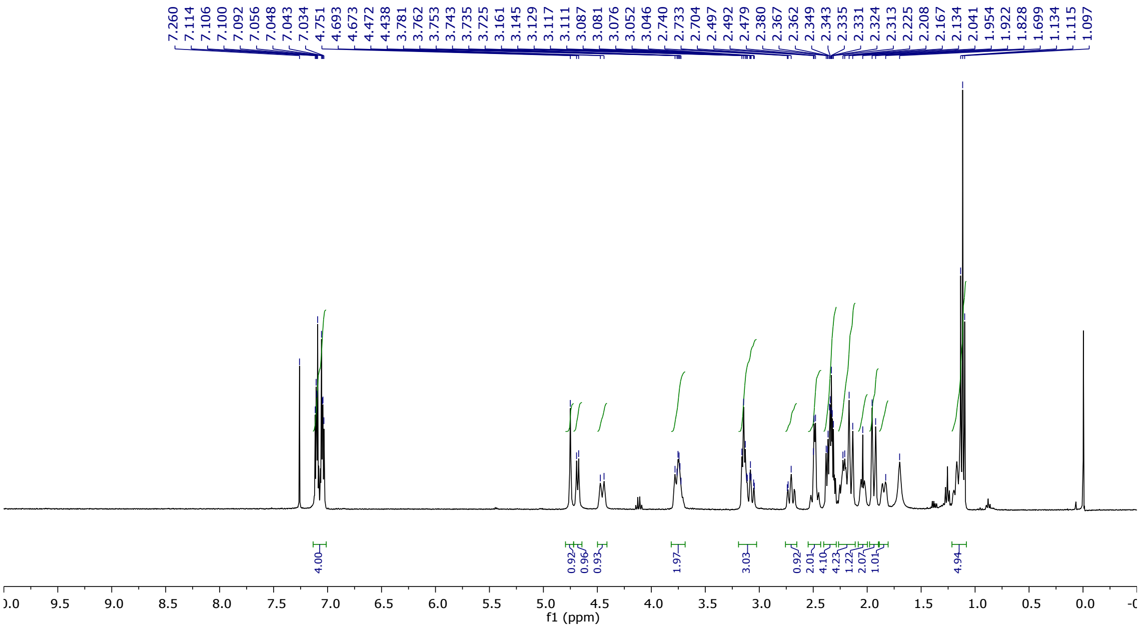
7.260 7.114 7.106 7.100 7.092 7.056 7.048 7.043 7.034 4.751 4.693 4.673 4.472 4.438 3.781 3.762 3.753 3.743 3.735 3.725 3.161 3.145 3.129 3.117 3.111 3.087 3.081 3.076 3.052 3.046 2.740 2.733 2.704 2.497 2.492 2.479 2.380 2.367 2.362 2.349 2.343 2.335 2.331 2.324 2.313 2.225 2.208 2.167 2.134 2.041 1.954 1.922 1.828 1.699 1.134 1.115 1.097
4.00
0.92
0.96
0.93
1.97
3.03
0.92
2.01
4.10
4.23
1.22
2.07
1.01
4.94
f1 (ppm)

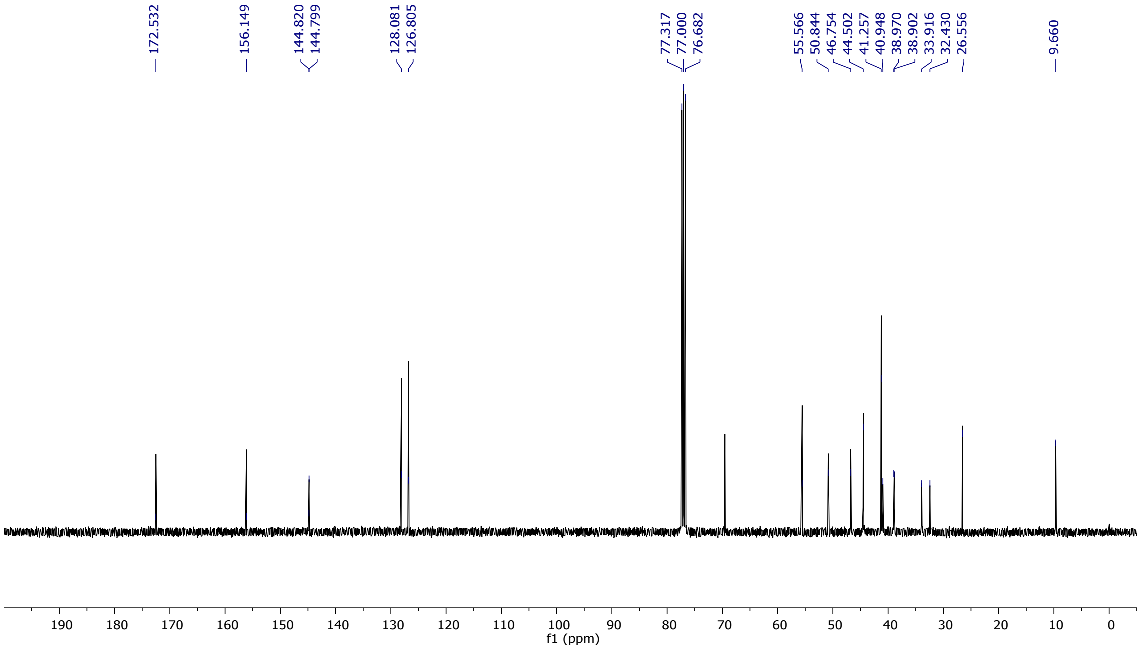
172.532
156.149
144.820
144.799
128.081
126.805
77.317
77.000
76.682
55.566
50.844
46.754
44.502
41.257
40.948
38.970
38.902
33.916
32.430
26.556
9.660
190 180 170 160 150 140 130 120 110 100 90 80 70 60 50 40 30 20 10 0
f1 (ppm)

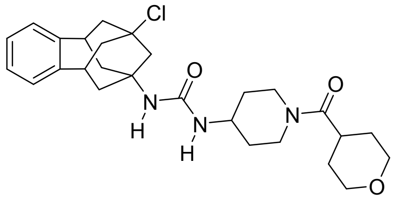
Cl
O
N
H
N
H
N
O
O

15

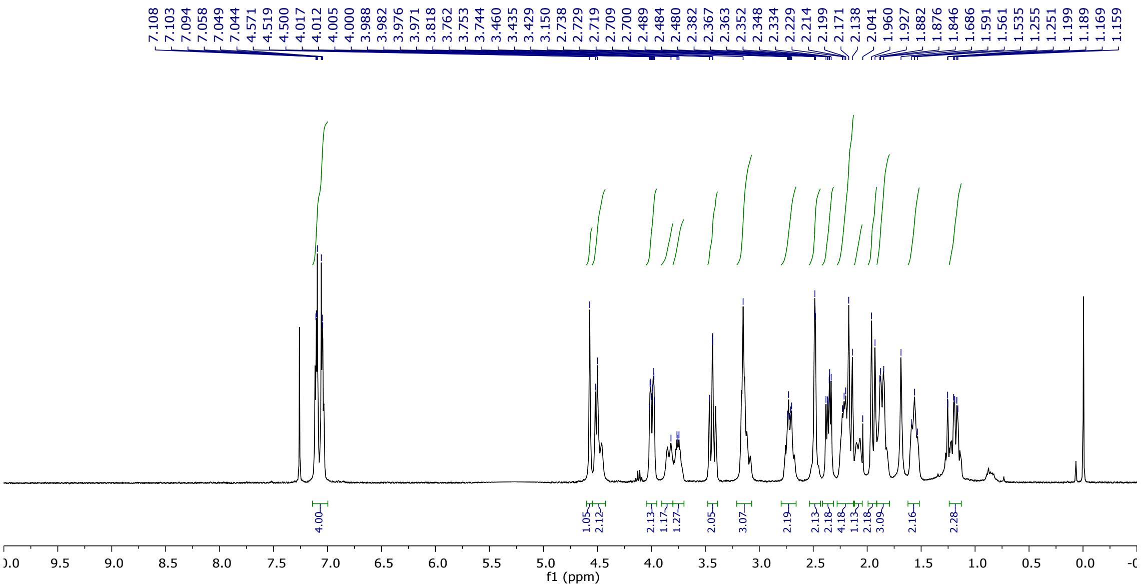
7.108
7.103
7.094
7.058
7.049
7.044
4.571
4.519
4.500
4.017
4.012
4.005
4.000
3.988
3.982
3.976
3.971
3.818
3.762
3.753
3.744
3.460
3.435
3.429
3.150
2.738
2.729
2.719
2.709
2.700
2.489
2.484
2.480
2.382
2.367
2.363
2.352
2.348
2.334
2.229
2.214
2.199
2.171
2.138
2.041
1.960
1.927
1.882
1.876
1.846
1.686
1.591
1.561
1.535
1.255
1.251
1.199
1.189
1.169
1.159
4.00
1.05
2.12
2.13
1.17
1.27
2.05
3.07
2.19
2.13
2.18
4.18
1.13
2.18
3.09
2.16
2.28
f1 (ppm)

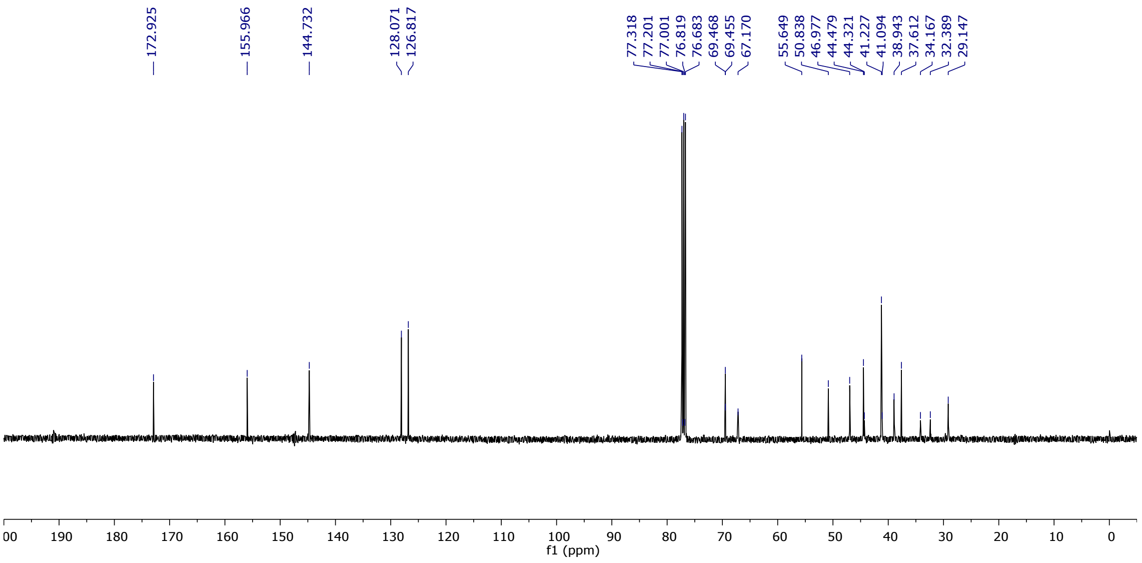
172.925
155.966
144.732
128.071
126.817
77.318
77.201
77.001
76.819
76.683
69.468
69.455
67.170
55.649
50.838
46.977
44.479
44.321
41.227
41.094
38.943
37.612
34.167
32.389
29.147
f1 (ppm)

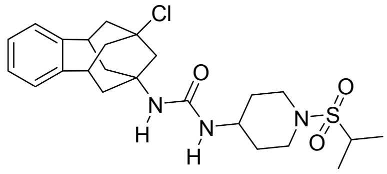
Cl
O
N
H
N
H
N
S
O
O

16

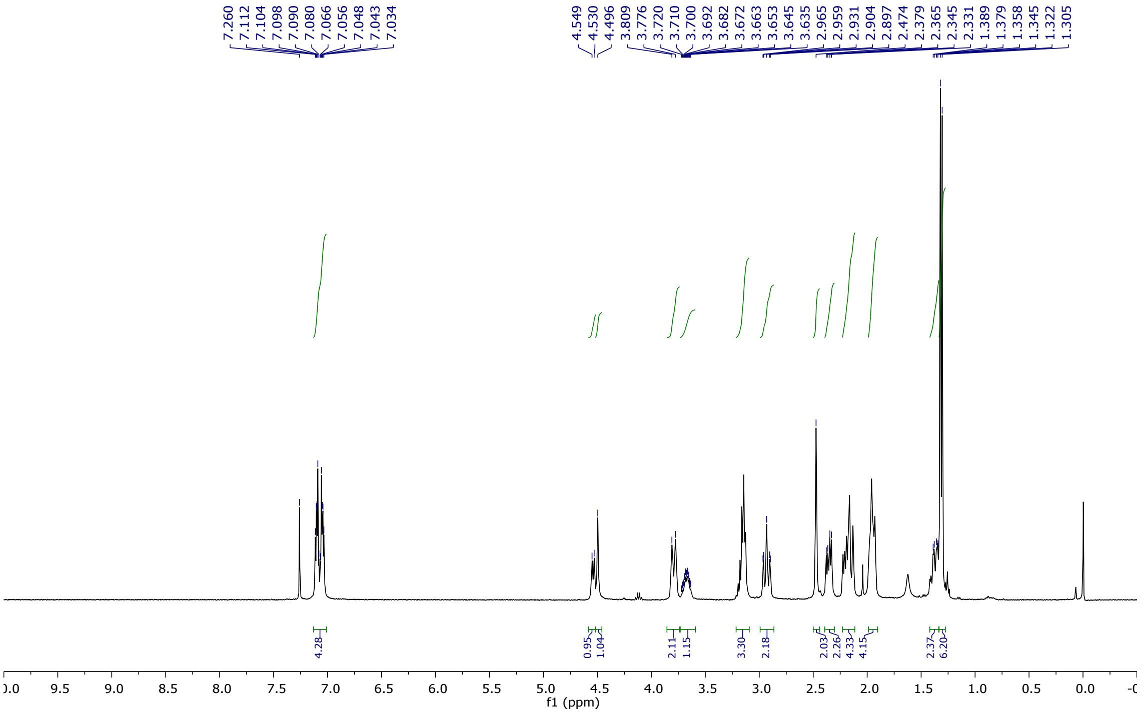
7.260
7.112
7.104
7.098
7.090
7.080
7.066
7.056
7.048
7.043
7.034
4.549
4.530
4.496
3.809
3.776
3.720
3.710
3.700
3.692
3.682
3.672
3.663
3.653
3.645
3.635
2.965
2.959
2.931
2.904
2.897
2.474
2.379
2.365
2.345
2.331
1.389
1.379
1.358
1.345
1.322
1.305
4.28
0.95
1.04
2.11
1.15
3.30
2.18
2.03
2.26
4.33
4.15
2.37
6.20
f1 (ppm)

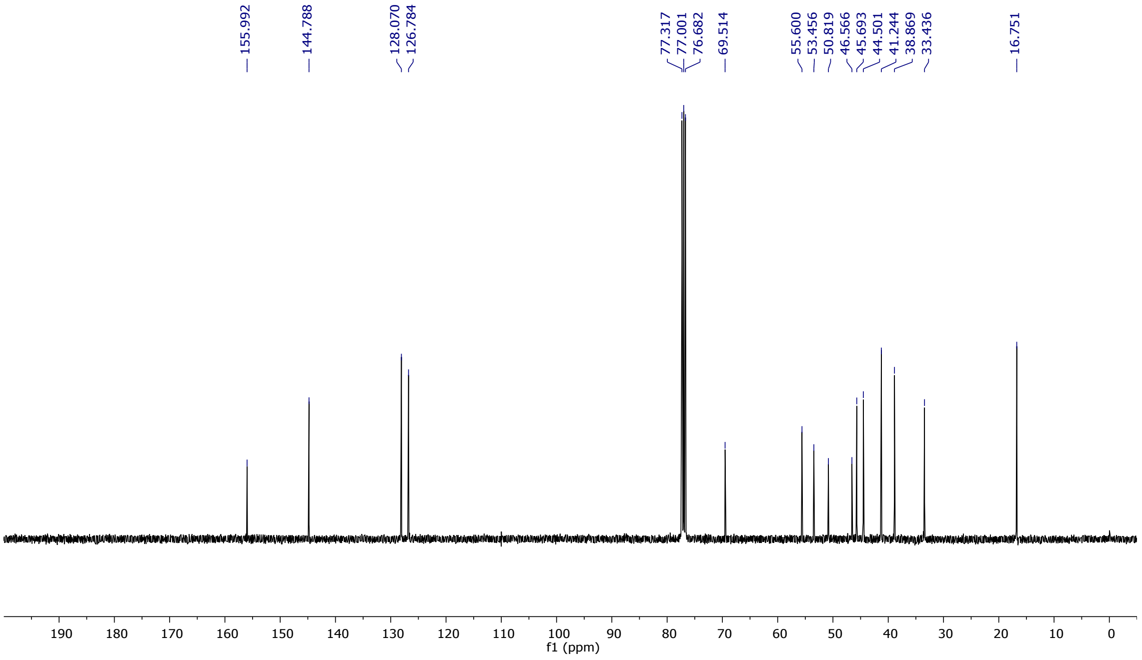
155.992
144.788
128.070
126.784
77.317
77.001
76.682
69.514
55.600
53.456
50.819
46.566
45.693
44.501
41.244
38.869
33.436
16.751
f1 (ppm)

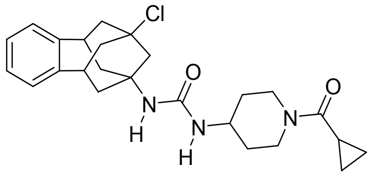

Cl
O
N
H
N
H
N
O

17

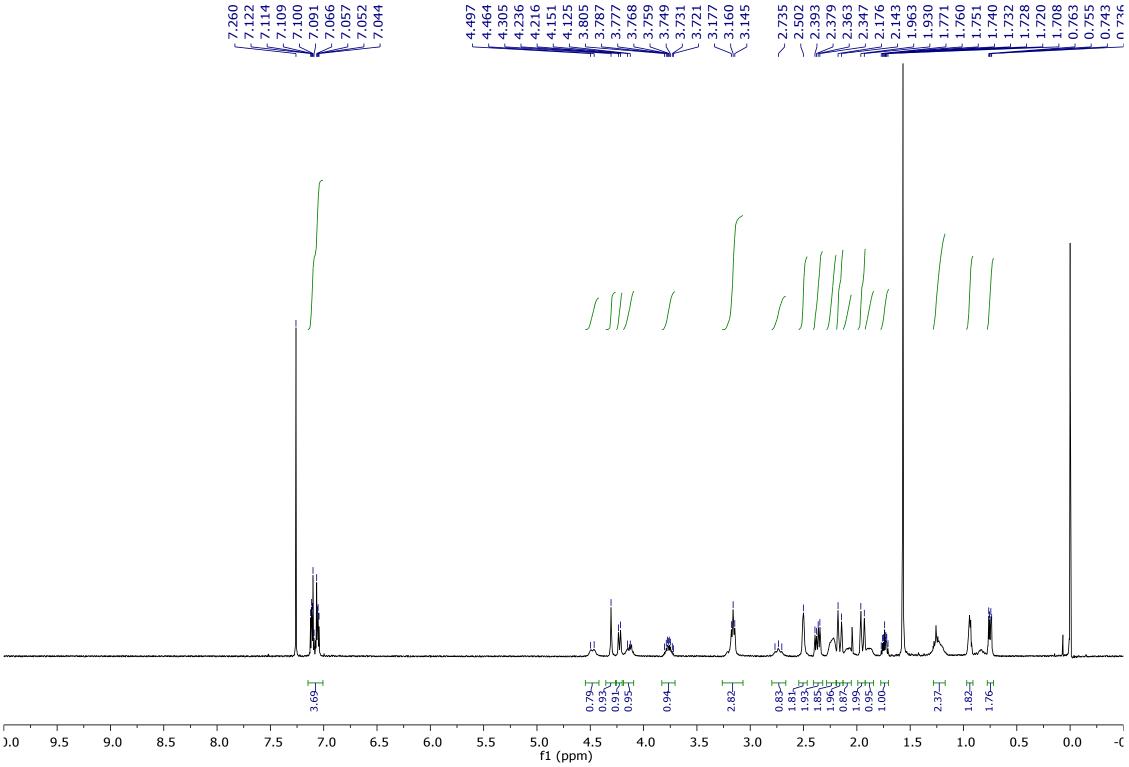

7.260
7.122
7.114
7.109
7.100
7.091
7.066
7.057
7.052
7.044
4.497
4.464
4.305
4.236
4.216
4.151
4.125
3.805
3.787
3.777
3.768
3.759
3.749
3.731
3.721
3.177
3.160
3.145
2.735
2.502
2.393
2.379
2.363
2.347
2.176
2.143
1.963
1.930
1.771
1.760
1.751
1.740
1.732
1.728
1.720
1.708
0.763
0.755
0.743
3.69
0.79
0.93
0.91
0.95
0.94
2.82
0.83
1.81
1.93
1.85
1.96
0.87
1.99
0.95
1.00
2.37
1.82
1.76
f1 (ppm)

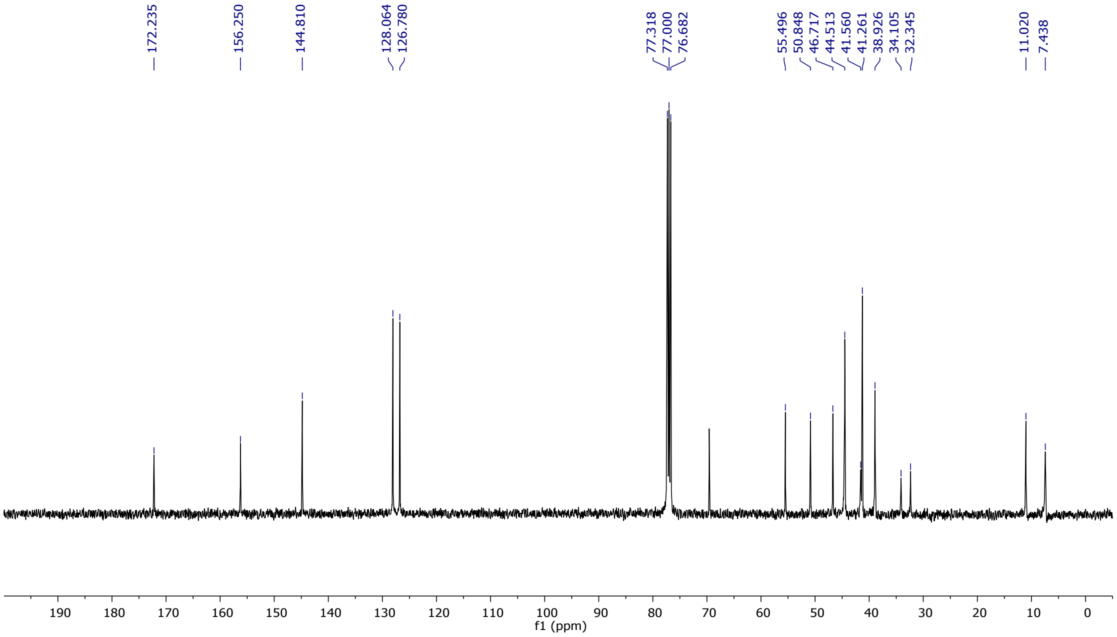

172.235
156.250
144.810
128.064
126.780
77.318
77.000
76.682
55.496
50.848
46.717
44.513
41.560
41.261
38.926
34.105
32.345
11.020
7.438
f1 (ppm)

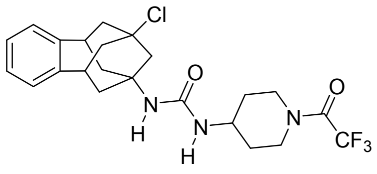
Cl
O
N
H
N
H
N
O
CF3

18

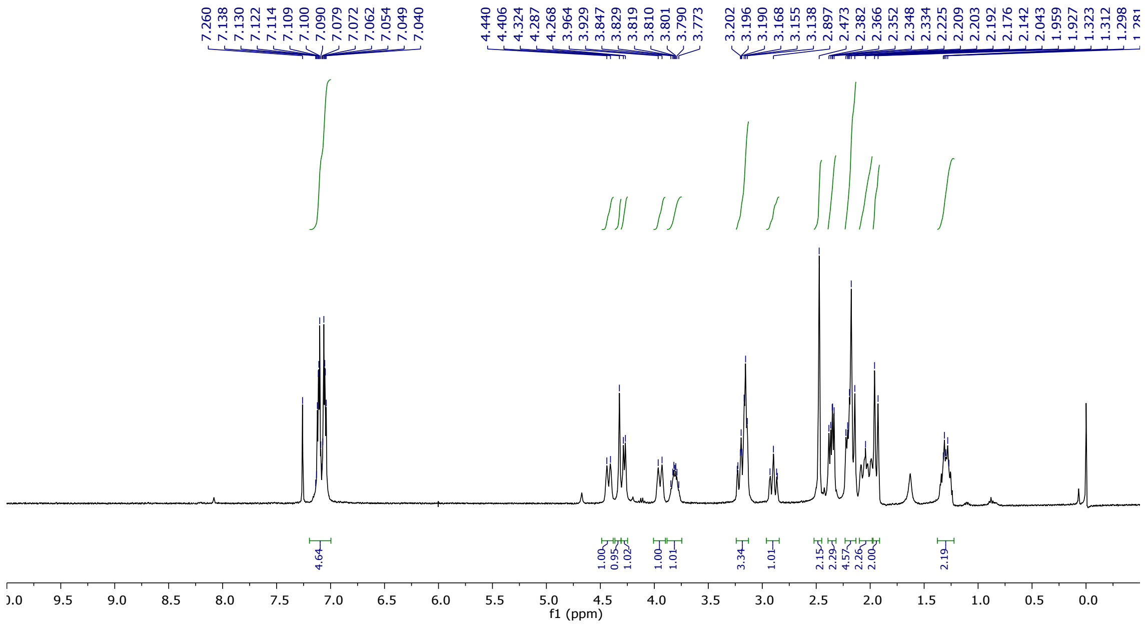
7.260
7.138
7.130
7.122
7.114
7.109
7.100
7.090
7.079
7.072
7.062
7.054
7.049
7.040
4.440
4.406
4.324
4.287
4.268
3.964
3.929
3.847
3.829
3.819
3.810
3.801
3.790
3.773
3.202
3.196
3.190
3.168
3.155
3.138
2.897
2.473
2.382
2.366
2.352
2.348
2.334
2.225
2.209
2.203
2.192
2.176
2.142
2.043
1.959
1.927
1.323
1.312
1.298
4.64
1.00
0.95
1.02
1.00
1.01
3.34
1.01
2.15
2.29
4.57
2.26
2.00
2.19
f1 (ppm)

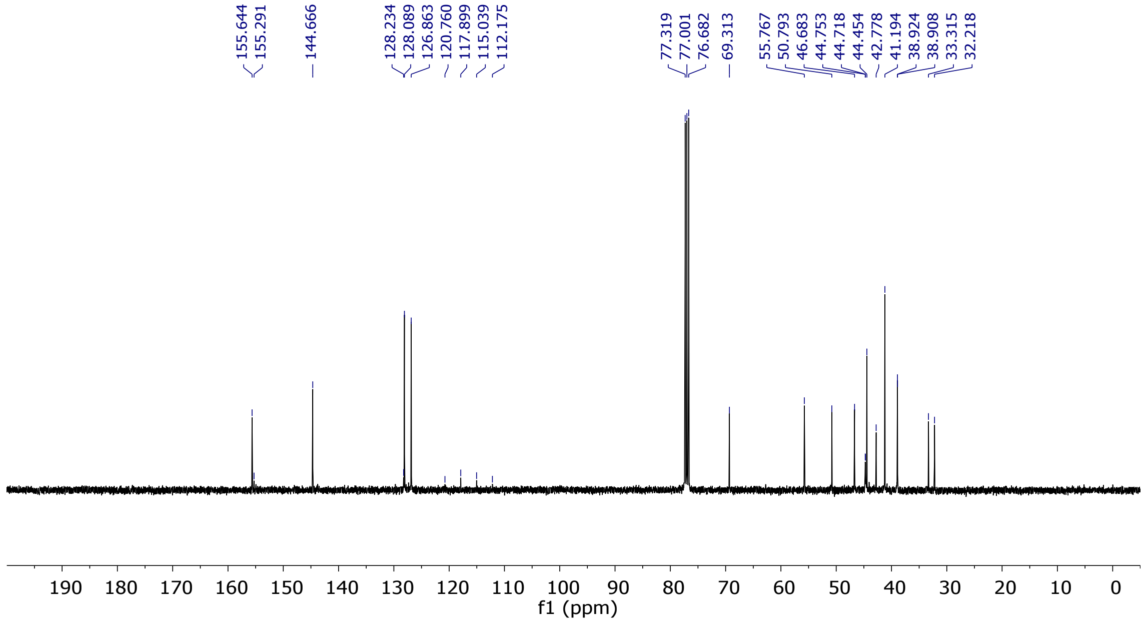
155.644
155.291
144.666
128.234
128.089
126.863
120.760
117.899
115.039
112.175
77.319
77.001
76.682
69.313
55.767
50.793
46.683
44.753
44.718
44.454
42.778
41.194
38.924
38.908
33.315
32.218
f1 (ppm)

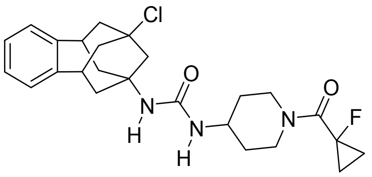

Cl
O
N
H
N
H
N
O
F

19

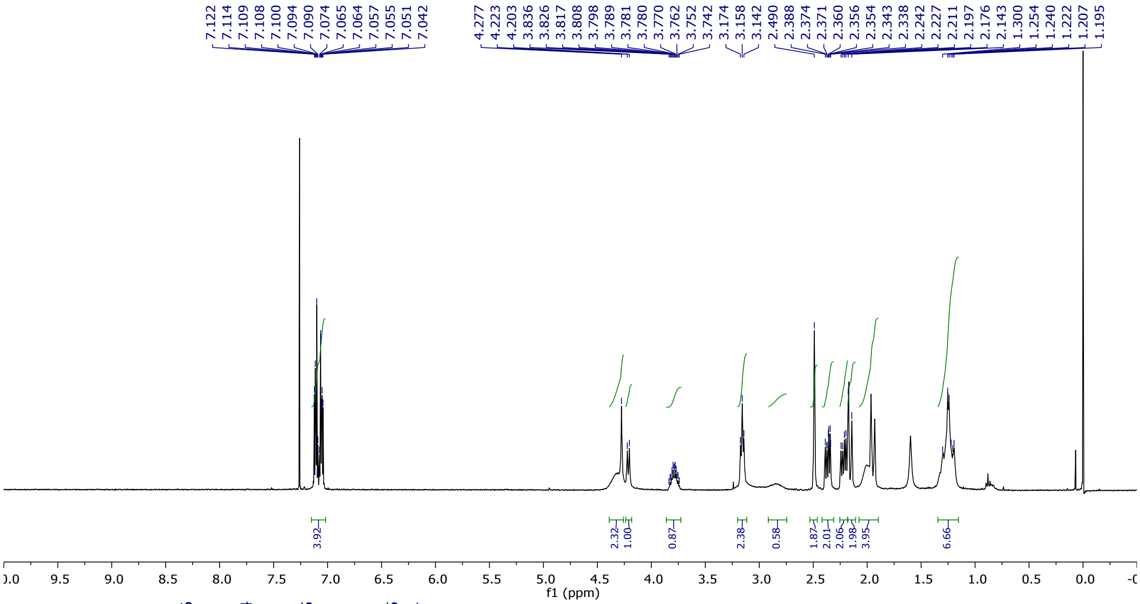

7.122
7.114
7.109
7.108
7.100
7.094
7.090
7.074
7.065
7.064
7.057
7.055
7.051
7.042
4.277
4.223
4.203
3.836
3.826
3.817
3.808
3.798
3.789
3.781
3.780
3.770
3.762
3.752
3.742
3.174
3.158
3.142
2.490
2.388
2.374
2.371
2.360
2.356
2.354
2.343
2.338
2.242
2.227
2.211
2.197
2.176
2.143
1.300
1.254
1.240
1.222
1.207
1.195
3.92
2.32
1.00
0.87
2.38
0.58
1.87
2.01
2.06
1.98
3.95
6.66
9.5
9.0
8.5
8.0
7.5
7.0
6.5
6.0
5.5
5.0
4.5
4.0
3.5
3.0
2.5
2.0
1.5
1.0
0.5
0.0
f1 (ppm)

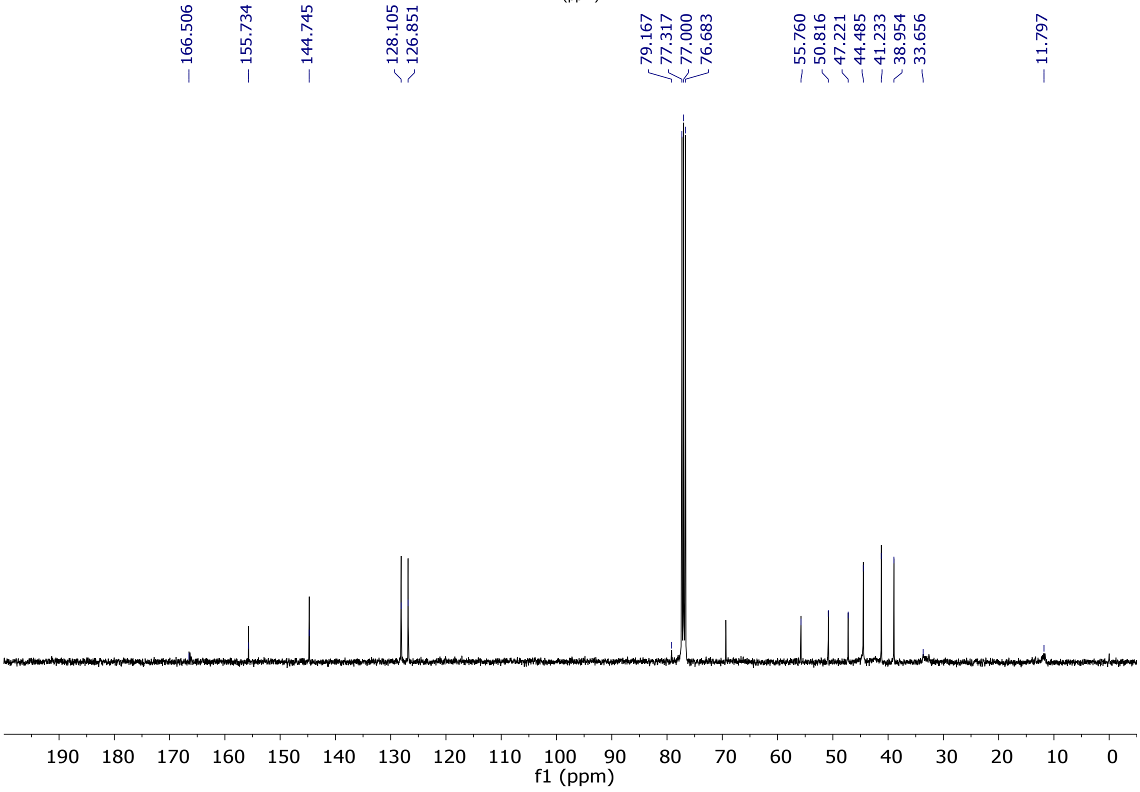

166.506
155.734
144.745
128.105
126.851
79.167
77.317
77.000
76.683
55.760
50.816
47.221
44.485
41.233
38.954
33.656
11.797
190
180
170
160
150
140
130
120
110
100
90
80
70
60
50
40
30
20
10
0
f1 (ppm)

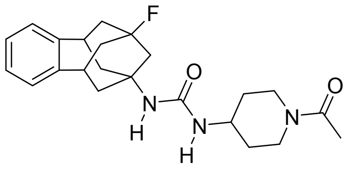
F
O
N
H
N
H
N
O

20

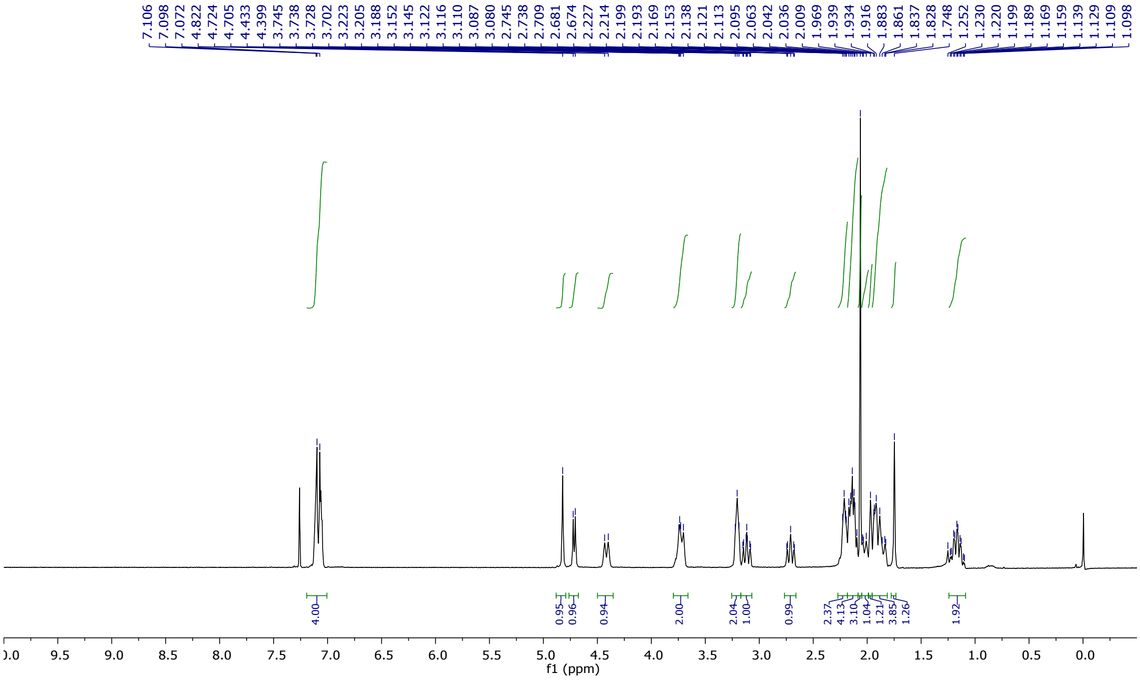
7.106
7.098
7.072
4.822
4.724
4.705
4.433
4.399
3.745
3.738
3.728
3.702
3.223
3.205
3.188
3.152
3.145
3.122
3.116
3.110
3.087
3.080
2.745
2.738
2.709
2.681
2.674
2.227
2.214
2.199
2.193
2.169
2.153
2.138
2.121
2.113
2.095
2.063
2.042
2.036
2.009
1.969
1.939
1.934
1.916
1.883
1.861
1.837
1.828
1.748
1.252
1.230
1.220
1.199
1.189
1.169
1.159
1.139
1.129
1.109
1.098
4.00
0.95
0.96
0.94
2.00
2.04
1.00
0.99
2.37
4.13
3.10
1.04
1.21
3.85
1.26
1.92
f1 (ppm)

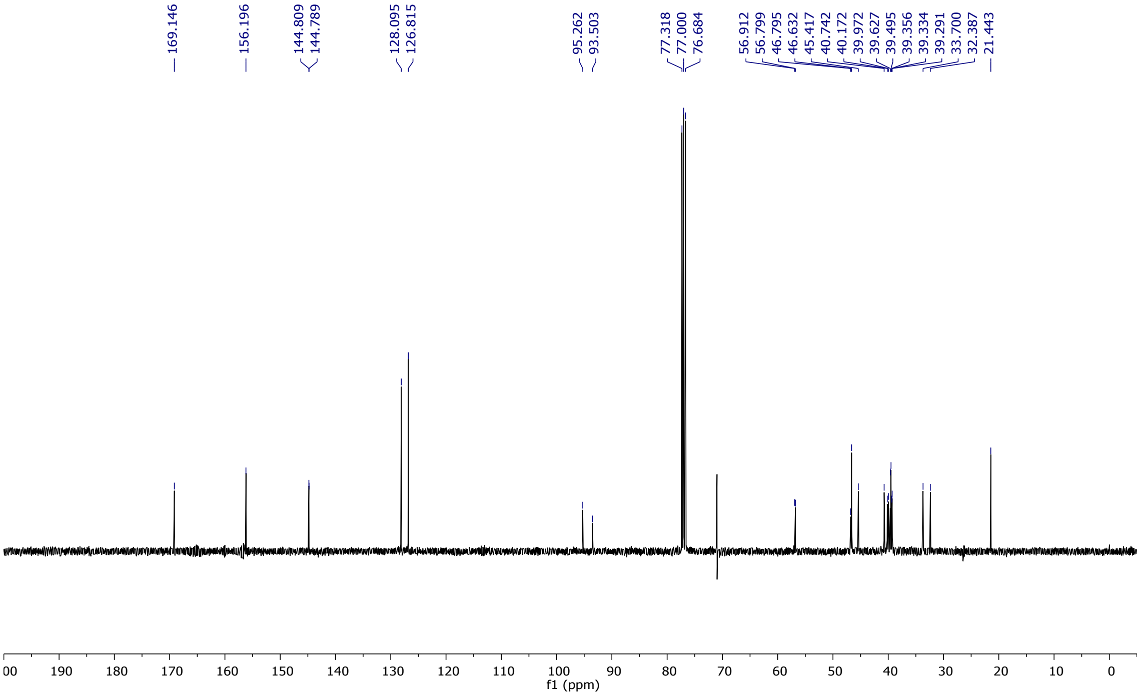
169.146
156.196
144.809
144.789
128.095
126.815
95.262
93.503
77.318
77.000
76.684
56.912
56.799
46.795
46.632
45.417
40.742
40.172
39.972
39.627
39.495
39.356
39.334
39.291
33.700
32.387
21.443
f1 (ppm)

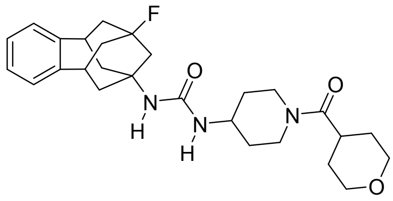

21

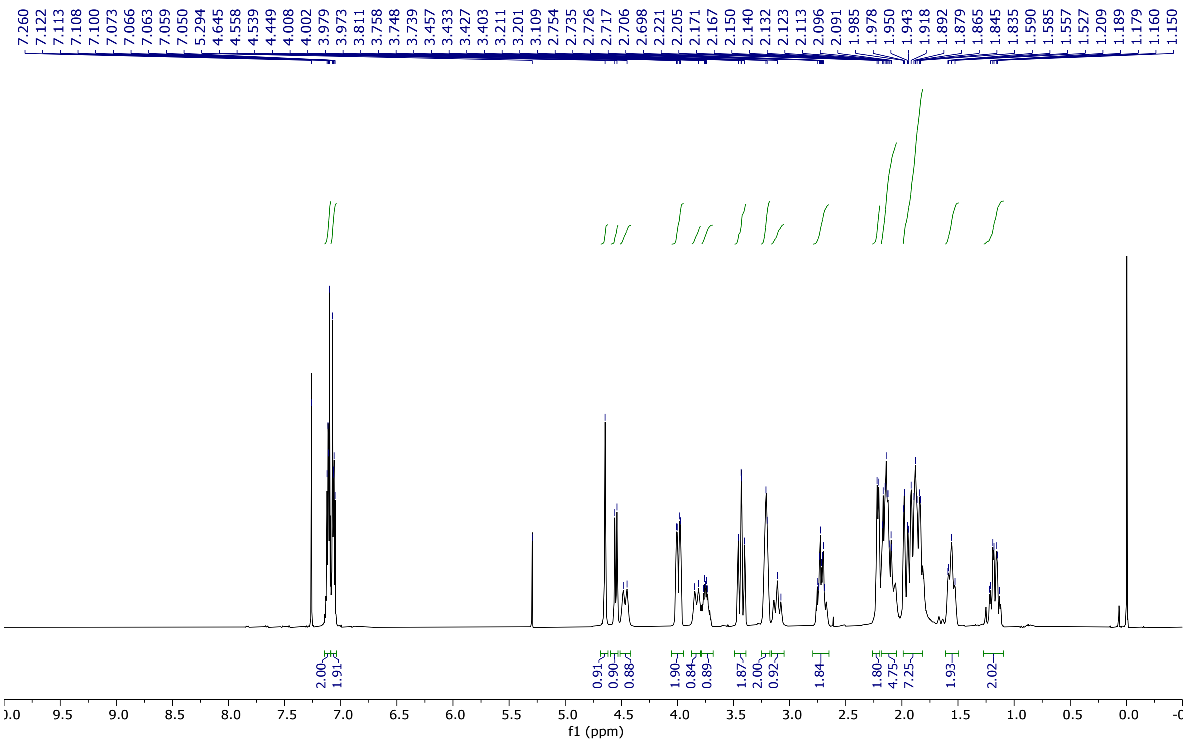

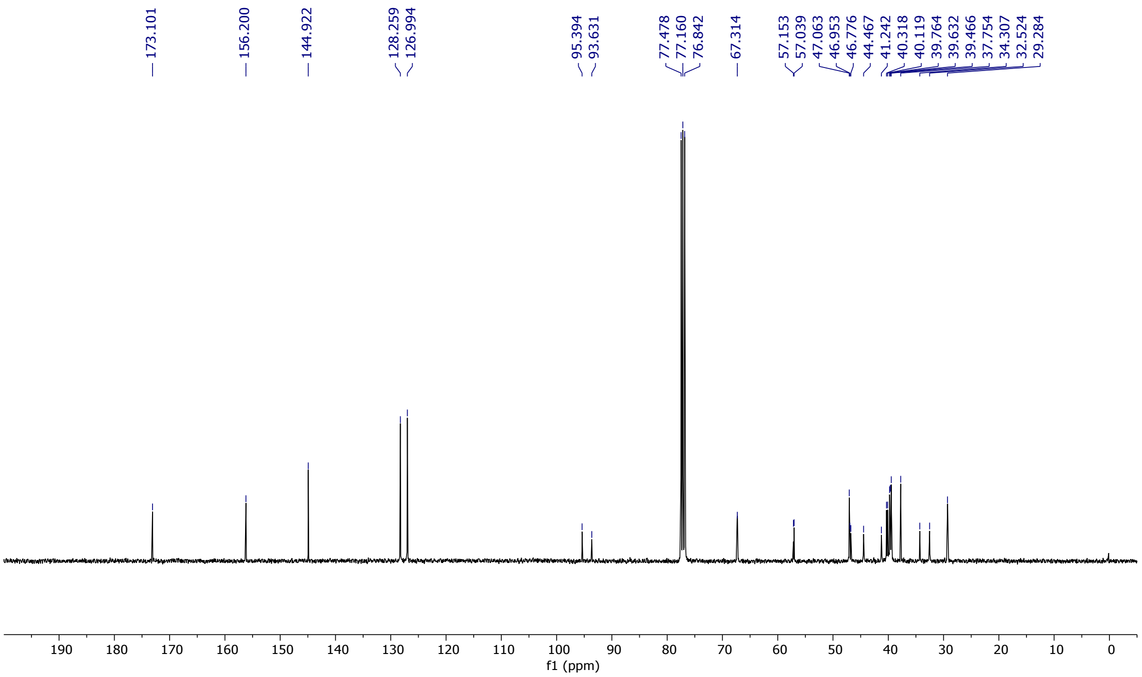

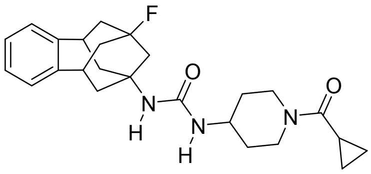
F
O
N
H
N
H
N
O

22

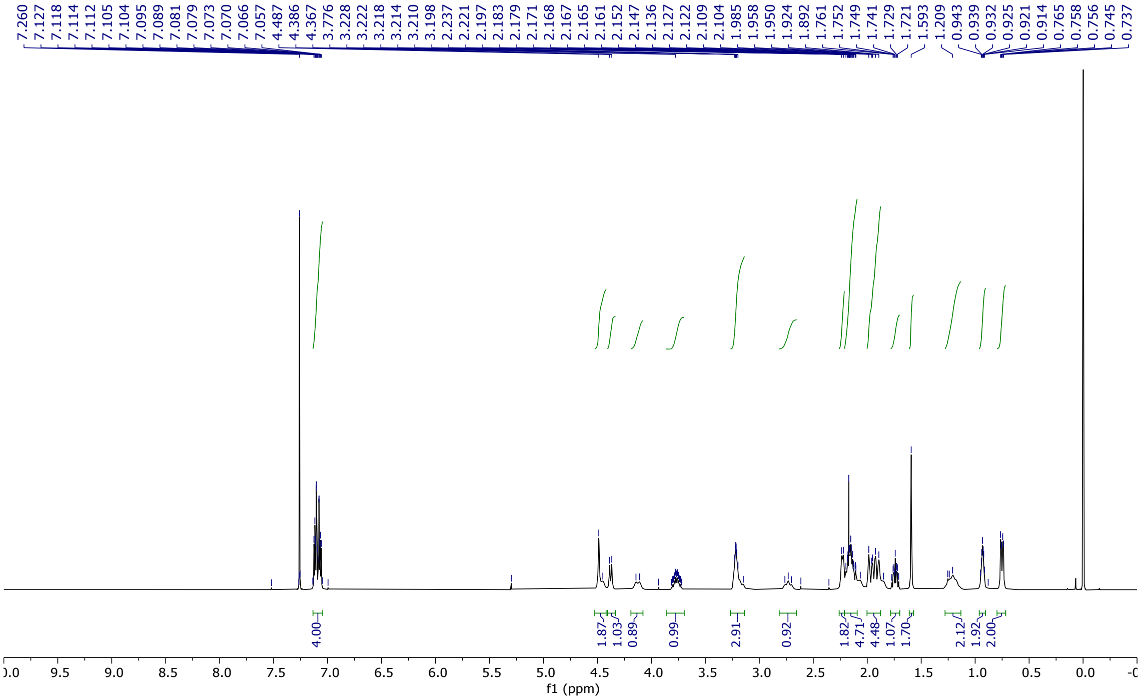
7.260
7.127
7.118
7.114
7.112
7.105
7.104
7.095
7.089
7.081
7.079
7.073
7.070
7.066
7.057
4.487
4.386
4.367
3.776
3.228
3.222
3.218
3.214
3.210
3.198
2.237
2.221
2.197
2.183
2.179
2.171
2.168
2.167
2.165
2.161
2.152
2.147
2.136
2.127
2.122
2.109
2.104
1.985
1.958
1.950
1.924
1.892
1.761
1.752
1.749
1.741
1.729
1.721
1.593
1.209
0.943
0.939
0.932
0.925
0.921
0.914
0.765
0.758
0.756
0.745
0.737
4.00
1.87
1.03
0.89
0.99
2.91
0.92
1.82
4.71
4.48
1.07
1.70
2.12
1.92
2.00
f1 (ppm)

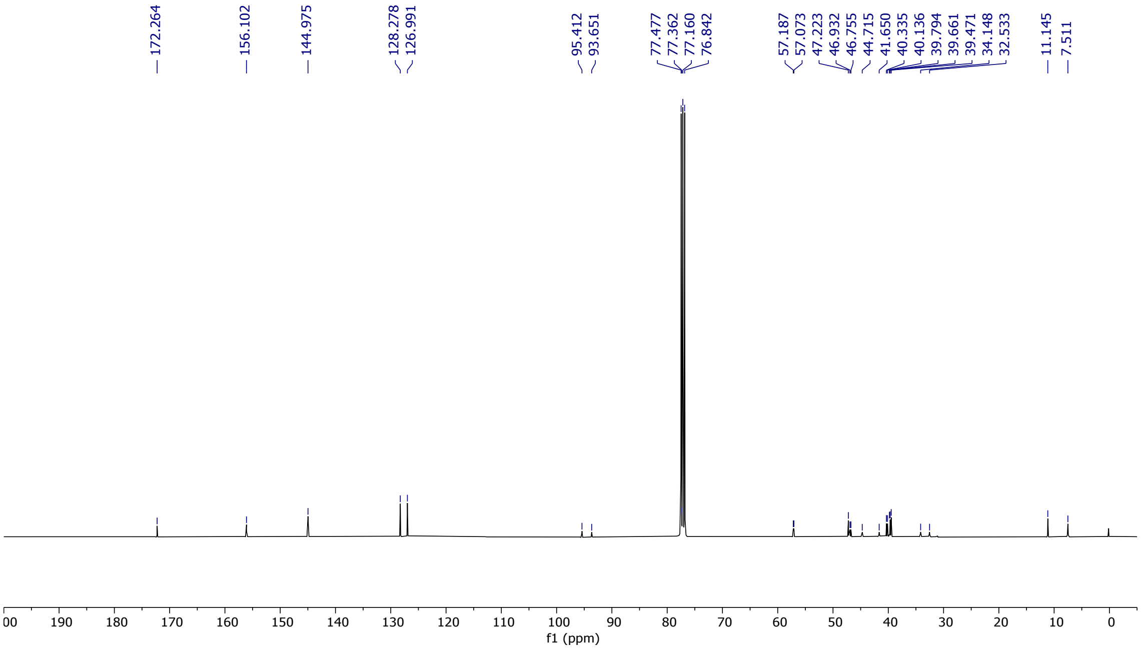
172.264
156.102
144.975
128.278
126.991
95.412
93.651
77.477
77.362
77.160
76.842
57.187
57.073
47.223
46.932
46.755
44.715
41.650
40.335
40.136
39.794
39.661
39.471
34.148
32.533
11.145
7.511
f1 (ppm)

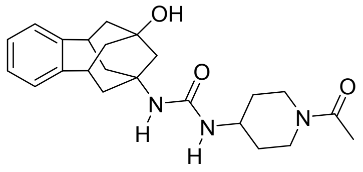
OH
O
N
H
N
H
N
O

23

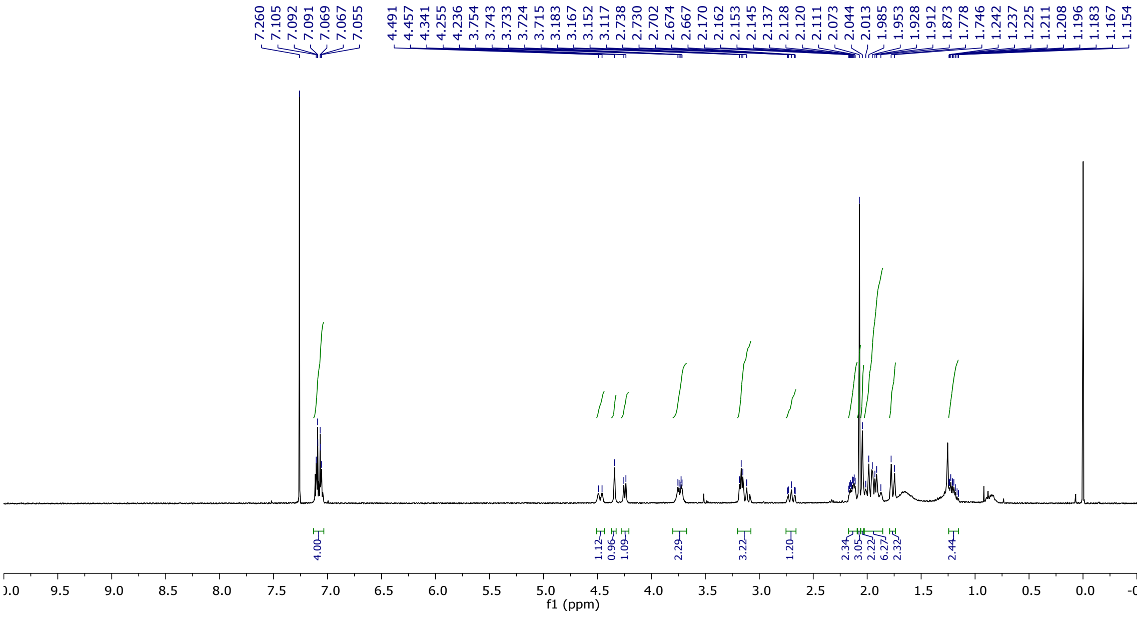
7.260
7.105
7.092
7.091
7.069
7.067
7.055
4.491
4.457
4.341
4.255
4.236
3.754
3.743
3.733
3.724
3.715
3.183
3.167
3.152
3.117
2.738
2.730
2.702
2.674
2.667
2.170
2.162
2.153
2.145
2.137
2.128
2.120
2.111
2.073
2.044
2.013
1.985
1.953
1.928
1.912
1.873
1.778
1.746
1.242
1.237
1.225
1.211
1.208
1.196
1.183
1.167
1.154
4.00
1.12
0.96
1.09
2.29
3.22
1.20
2.34
3.05
2.22
6.27
2.32
2.44
f1 (ppm)

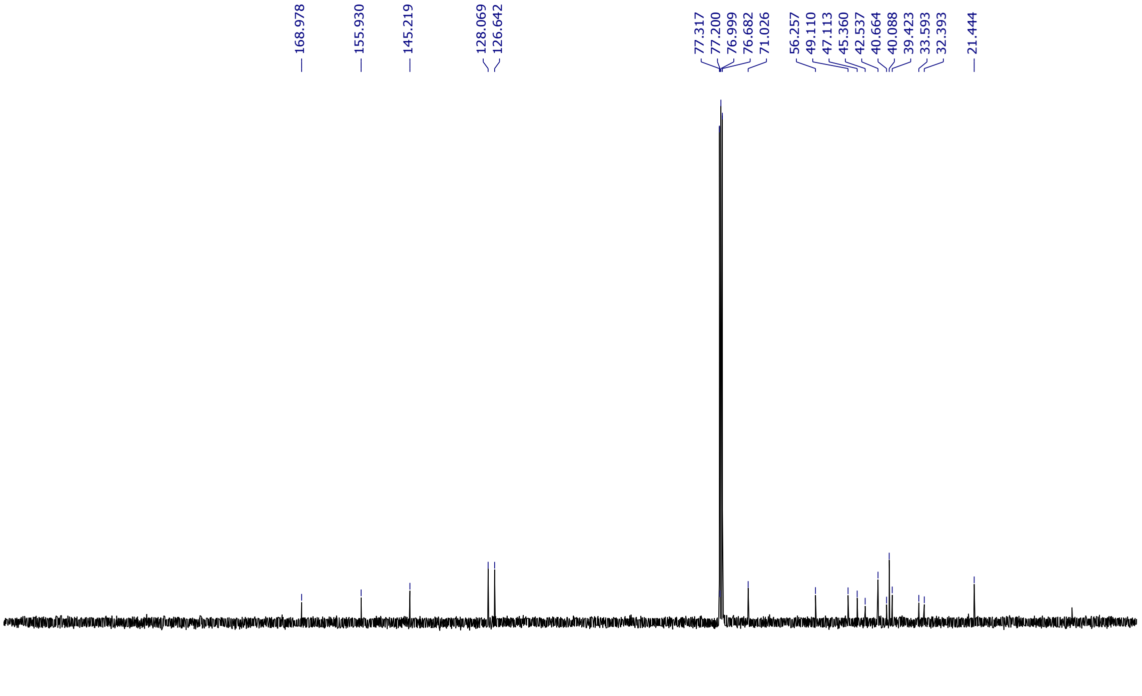
168.978
155.930
145.219
128.069
126.642
77.317
77.200
76.999
76.682
71.026
56.257
49.110
47.113
45.360
42.537
40.664
40.088
39.423
33.593
32.393
21.444
f1 (ppm)

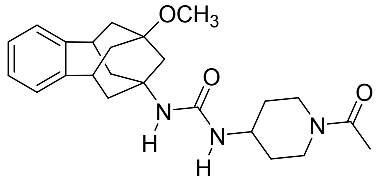

OCH3
O
N
H
N
H
N
O

24

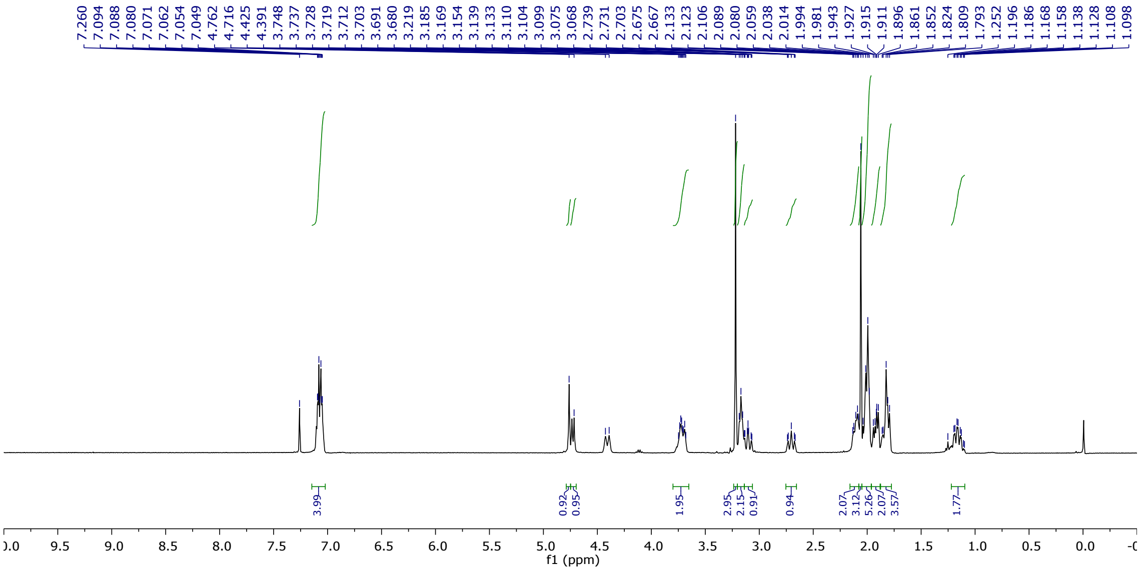

7.260 7.094 7.088 7.080 7.071 7.062 7.054 7.049 4.762 4.716 4.425 4.391 3.748 3.737 3.728 3.719 3.712 3.703 3.691 3.680 3.219 3.185 3.169 3.154 3.139 3.133 3.110 3.104 3.099 3.075 3.068 2.739 2.731 2.703 2.675 2.667 2.133 2.123 2.106 2.089 2.080 2.059 2.038 2.014 1.994 1.981 1.943 1.927 1.915 1.911 1.896 1.861 1.852 1.824 1.809 1.793 1.252 1.196 1.168 1.158 1.138 1.128 1.108 1.098
3.99 0.92 0.95 1.95 2.95 2.15 0.91 0.94 2.07 3.12 5.26 2.07 3.57 1.77
f1 (ppm)

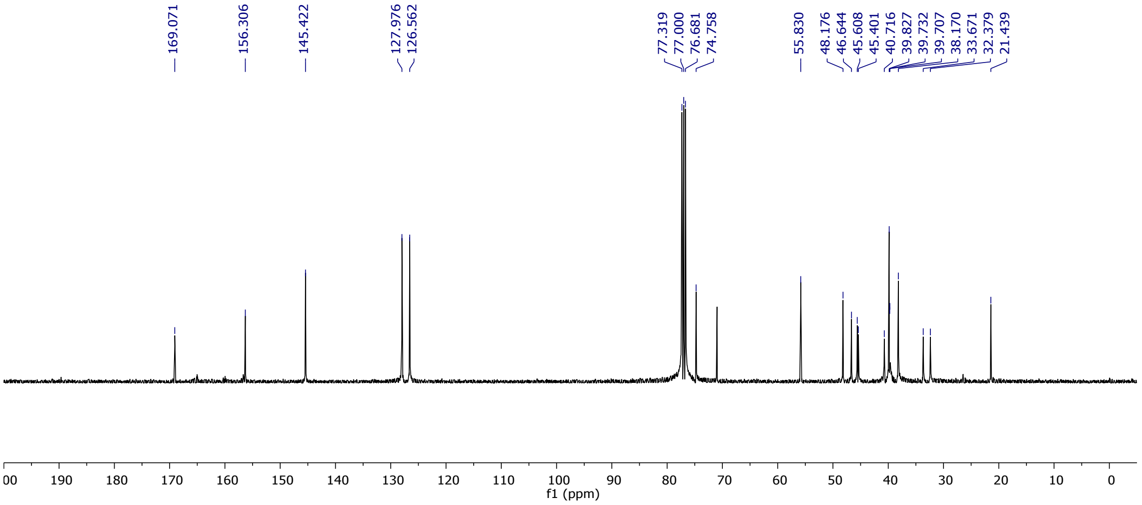

169.071 156.306 145.422 127.976 126.562 77.319 77.000 76.681 74.758 55.830 48.176 46.644 45.608 45.401 40.716 39.827 39.732 39.707 38.170 33.671 32.379 21.439
f1 (ppm)

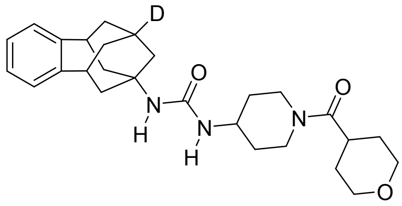

25

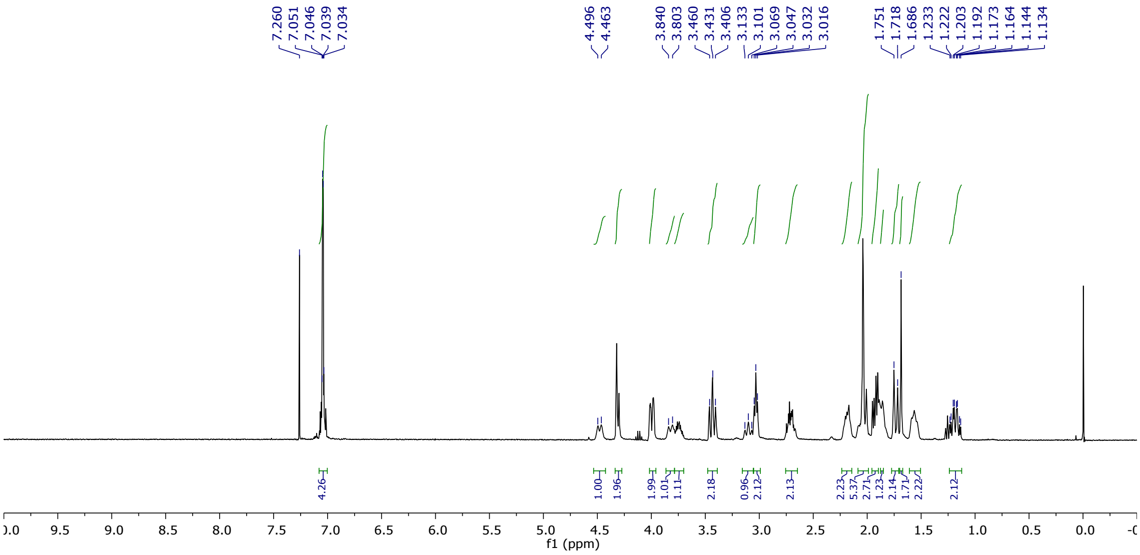
7.260
7.051
7.046
7.039
7.034
4.496
4.463
3.840
3.803
3.460
3.431
3.406
3.133
3.101
3.069
3.047
3.032
3.016
1.751
1.718
1.686
1.233
1.222
1.203
1.192
1.173
1.164
1.144
1.134
f1 (ppm)

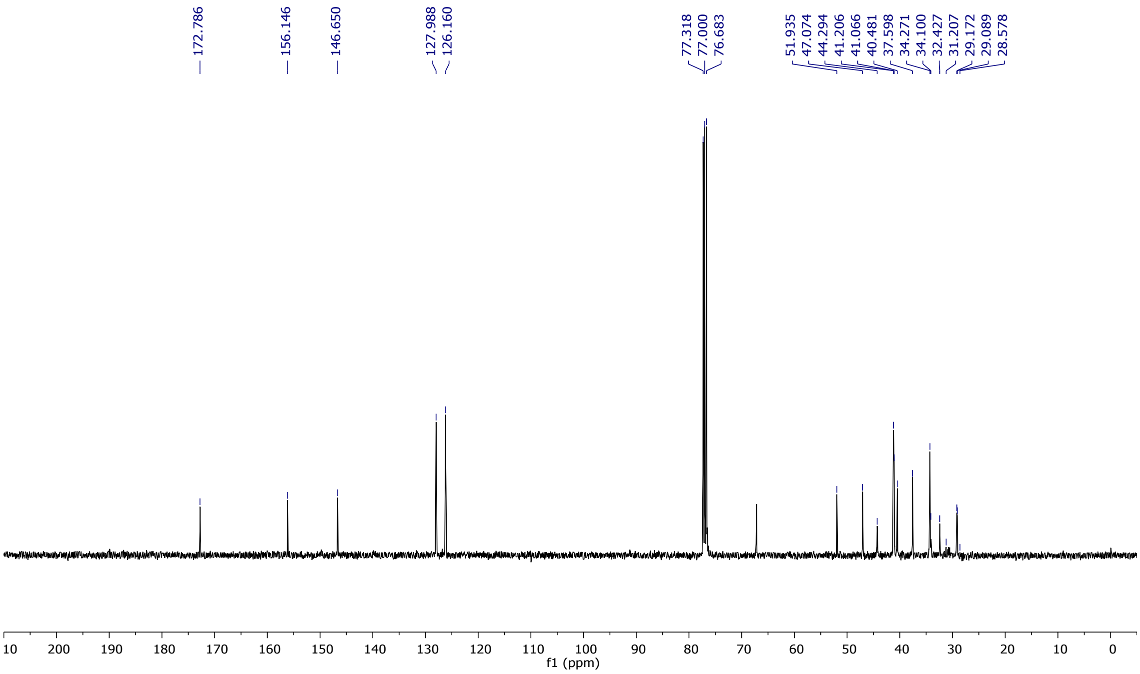
172.786
156.146
146.650
127.988
126.160
77.318
77.000
76.683
51.935
47.074
44.294
41.206
41.066
40.481
37.598
34.271
34.100
32.427
31.207
29.172
29.089
28.578
f1 (ppm)

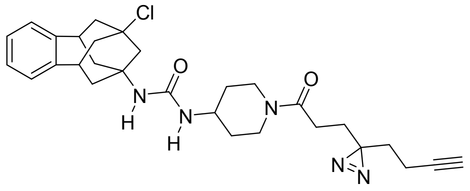
Cl
O
N
H
N
H
O
N
N
N
28

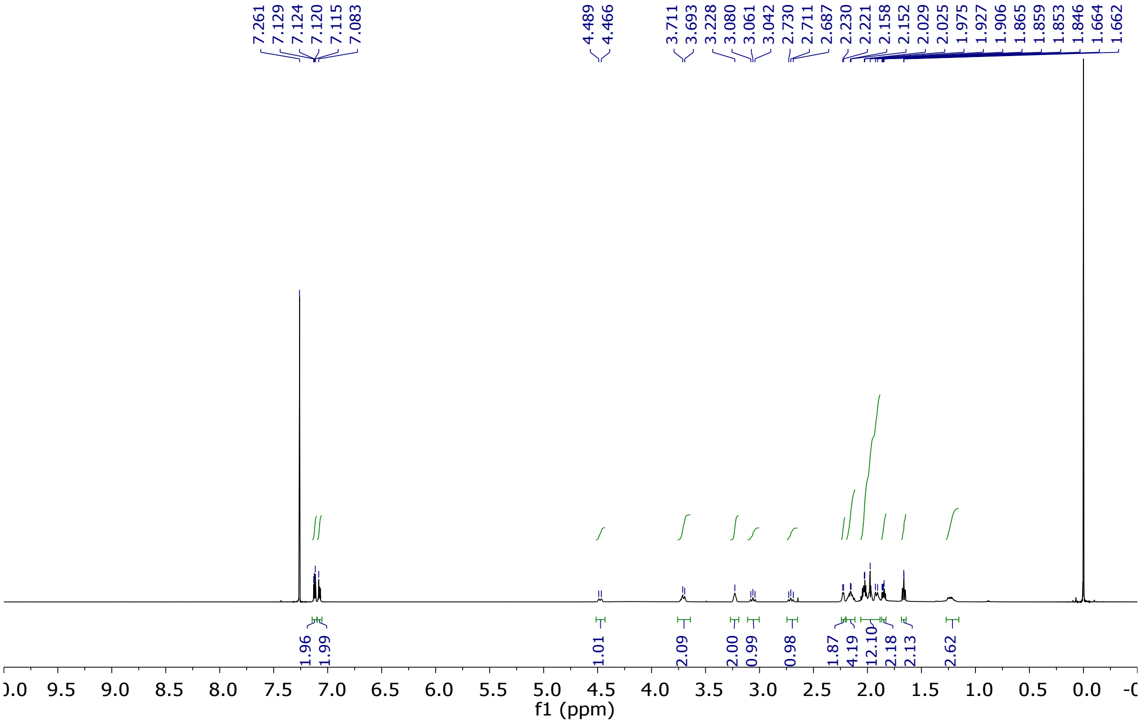
7.261
7.129
7.124
7.120
7.115
7.083
4.489
4.466
3.711
3.693
3.228
3.080
3.061
3.042
2.730
2.711
2.687
2.230
2.221
2.158
2.152
2.029
2.025
1.975
1.927
1.906
1.865
1.859
1.853
1.846
1.664
1.662
1.96
1.99
1.01
2.09
2.00
0.99
0.98
1.87
4.19
12.10
2.18
2.13
2.62
f1 (ppm)

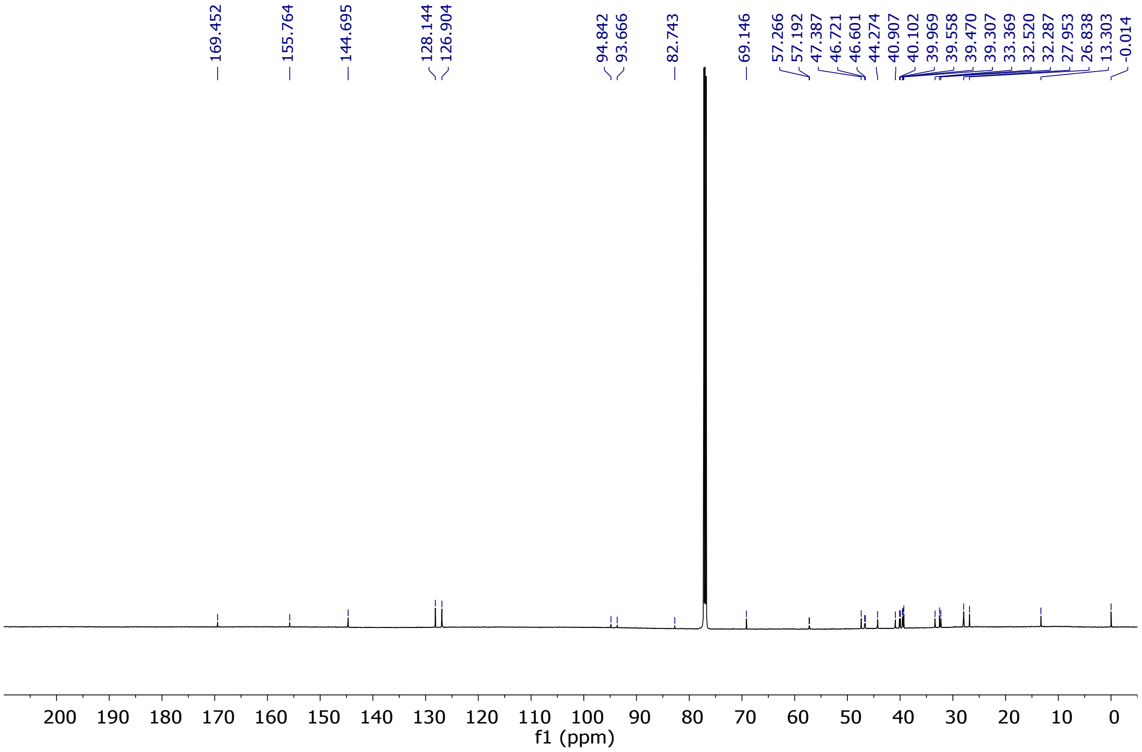
169.452
155.764
144.695
128.144
126.904
94.842
93.666
82.743
69.146
57.266
57.192
47.387
46.721
46.601
44.274
40.907
40.102
39.969
39.558
39.470
39.307
33.369
32.520
32.287
27.953
26.838
13.303
-0.014
f1 (ppm)

Compound	Molecular Formula	Calculated			Found		
		C	H	N	C	H	N
**9**	$C_{25}H_{35}N_3O_2 \cdot 0.25\ H_2O$	72.52	8.64	10.15	72.65	8.49	9.82
**10**	$C_{28}H_{39}N_3O_3$	72.23	8.44	9.02	72.33	8.40	8.83
**11**	$C_{25}H_{37}N_3O_3S$	65.33	8.11	9.14	65.41	8.31	8.93
**12**	$C_{26}H_{35}N_3O_2 \cdot 0.1\ CH_2Cl_2$	72.89	8.25	9.77	73.08	8.23	9.53
**13**	$C_{23}H_{30}ClN_3O_2 \cdot 0.75$ EtOAc	64.78	7.53	8.72	64.73	7.56	8.89
**14**	$C_{24}H_{32}ClN_3O_2 \cdot 0.75\ H_2O$	65.00	7.61	9.47	65.27	7.51	9.15
**15**	$C_{27}H_{36}ClN_3O_3$	66.72	7.47	8.65	66.92	7.40	8.43
**16**	$C_{24}H_{34}ClN_3O_3S \cdot 0.05$ EtOAc	60.00	7.16	8.67	60.38	7.08	8.27
**17**	$C_{25}H_{32}ClN_3O_2 \cdot 0.75\ H_2O$	66.05	7.41	9.24	66.21	7.31	9.00
**18**	$C_{23}H_{27}ClF_3N_3O_2 \cdot 0.75\ CH_3OH$	57.75	6.12	8.51	58.04	5.82	8.20
**20**	$C_{23}H_{30}FN_3O_2 \cdot 0.5\ H_2O$	67.62	7.65	10.29	67.61	7.93	9.94
**21**	$C_{27}H_{36}FN_3O_3 \cdot 0.2\ CH_2Cl_2$	67.14	7.54	8.64	67.47	7.57	8.29
**22**	$C_{25}H_{32}FN_3O_2 \cdot 0.1\ CH_2Cl_2$	69.46	7.48	9.68	69.64	7.52	9.45
**23**	$C_{23}H_{31}N_3O_3 \cdot 1\ CH_3OH$	67.11	8.21	9.78	67.25	8.15	9.72
**24**	$C_{24}H_{33}N_3O_3$	70.04	8.08	10.21	69.63	8.28	9.86
**25**	$C_{27}H_{36}DN_3O_3 \cdot 1\ H_2O$	68.91	8.14	8.93	69.28	7.94	8.69

Table S1: Elemental analysis data.

HPLC blank

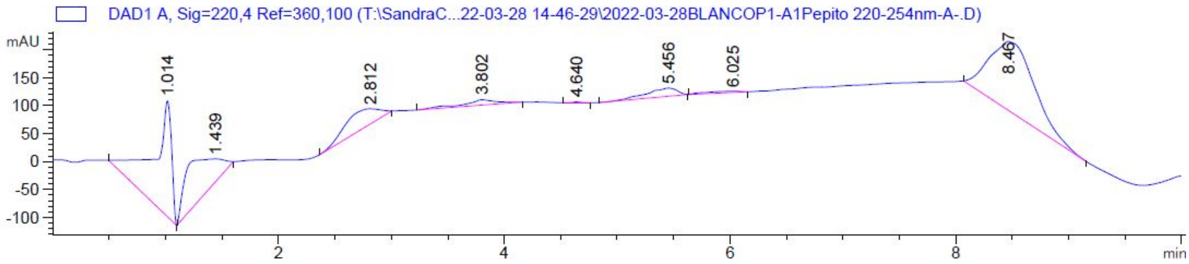

HPLC trace for compound **15**

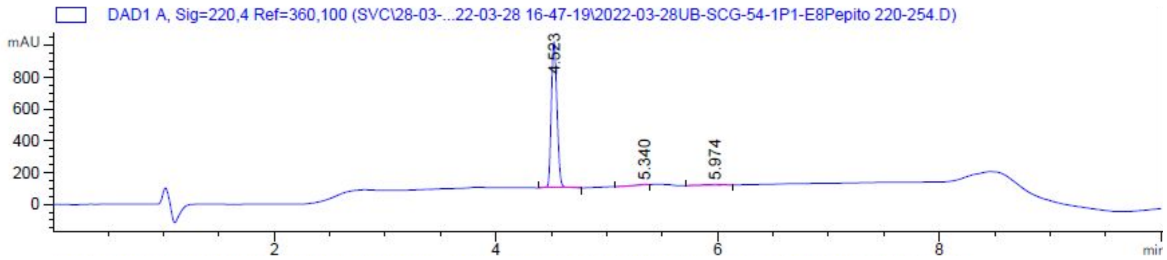

Signal 1: DAD1 A, Sig=220,4 Ref=360,100

Peak #	RetTime [min]	Type	Width [min]	Area [mAU*s]	Height [mAU]	Area %
1	4.523	BB	0.0579	3315.89185	922.69910	97.0873
2	5.340	BB	0.0977	24.28494	3.46684	0.7110
3	5.974	BB	0.1840	75.19284	5.77258	2.2016
Totals :				3415.36963	931.93853	

HPLC trace for compound **19**

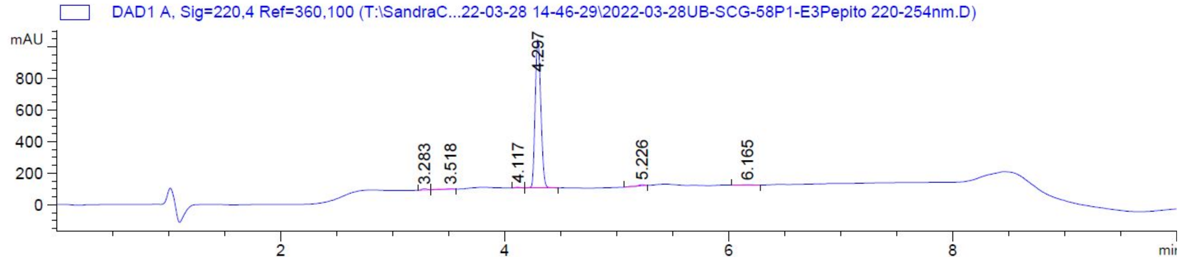

Signal 1: DAD1 A, Sig=220,4 Ref=360,100

Peak #	RetTime [min]	Type	Width [min]	Area [mAU*s]	Height [mAU]	Area %
1	3.283	BB	0.0461	24.11851	8.25013	0.7095
2	3.518	BB	0.0702	13.88129	2.78350	0.4083
3	4.117	BB	0.0505	14.65306	4.68819	0.4310
4	4.297	BB	0.0549	3305.63013	942.17554	97.2374
5	5.226	BB	0.0612	27.93230	6.90967	0.8216
6	6.165	BB	0.1294	13.32978	1.73950	0.3921

Totals : 3399.54506 966.54653

HPLC trace for compound **21**

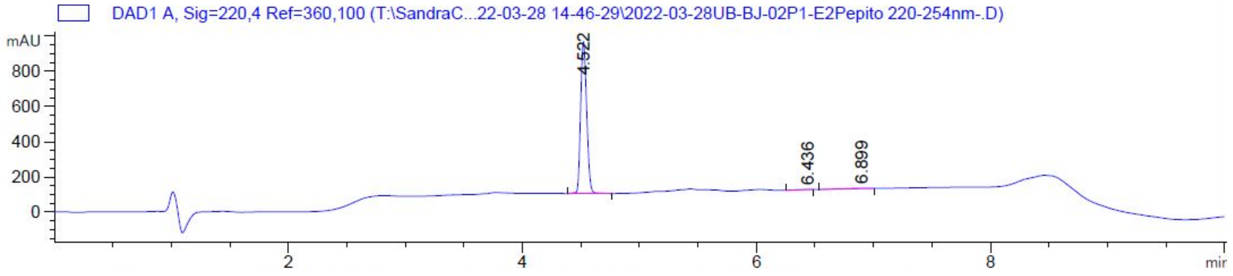

Signal 1: DAD1 A, Sig=220,4 Ref=360,100

Peak #	RetTime [min]	Type	Width [min]	Area [mAU*s]	Height [mAU]	Area %
1	4.522	BB	0.0578	3103.75317	865.27777	98.7808
2	6.436	BB	0.1077	10.30939	1.31215	0.3281
3	6.899	BB	0.1877	27.99917	2.09771	0.8911

Totals : 3142.06174 868.68762

Compound	5AM3 Orientation	5ALZ Orientation
**15**	-69.4 ± 3.4 kcal/mol	-59.2 ± 5.7 kcal/mol
**21**	-68.0 ± 3.9 kcal/mol	-62.4 ± 6.1 kcal/mol
**13**	-59.1 ± 3.6 kcal/mol	-56.1 ± 5.3 kcal/mol
**23**	-57.8 ± 4.8 kcal/mol	-58.5 ± 3.4 kcal/mol

Table S2. Values of MMGBSA calculations for compounds **15**, **21**, **13**, and **23** obtained from three replicas of 500 ns of molecular dynamics simulations for each compound. The simulations are performed in two possible orientations: 1. Similar to PDB 5AM3, i.e. with the benzohomoadamantane moiety in the LHS and the piperidine group in the RHS, respectively; 2. Similar to PDB 5ALZ, i.e. with the piperidine group in the LHS and the benzohomoadmantane scaffold in the RHS, respectively. All values are given in kcal/mol.

	Cytochrome inhibition[a]		
Compound	CYP 1A2	CYP 2D6	CYP 3A4[b] (DBF)
**15**	18 ± 1	43 ± 2	25 ± 3
**21**	24 ± 1	37 ± 3	8 ± 3
**22**	24 ± 2	13 ± 5	2 ± 1

Table S3. Inhibition (expressed as % of inhibition at 10 µM or IC_{50}) of recombinant human cytochromes P450 enzymes. [a]The cytochrome inhibition was tested at 10 µM. IC_{50} was calculated for those compounds that presented >50% of inhibition. [b]For the study of CYP3A4, two different substrates were used: benzyloxytrifluoromethylcoumarin (BFC) and dibenzylfluorescein (DBF).

Time	Mean (µg/mL)	SD (µg/mL)
0 h	0	0
0.5 h	0.54	0.052
0.75 h	1.21	0.15
1 h	0.45	0.12
2 h	0.82	0.078
4 h	0.15	0.005
6 h	0.018	0.001

Table S4. Mean of concentrations of compound **15** in mouse plasma at different times after subcutaneous administration at 5 mg/Kg.

Time	Mean (µg/mL)	SD (µg/mL)
0 h	0	0
0.25 h	19.1	2.7
0.5 h	11.9	6.48
1 h	2.5	2.42
2 h	1.0	0.55
3 h	1.05	0.07
4 h	0.32	0.21

6 h	0.56	0.02

Table S5. Mean of concentrations of compound **21** in mouse plasma at different times after subcutaneous administration at 5 mg/Kg.

a) *Spontaneous binding accelerated molecular dynamics simulation (aMD) Compound 15*

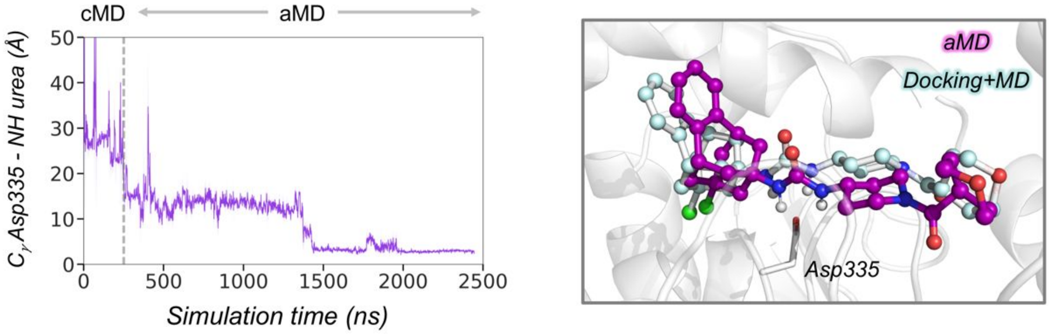

Figure S1. Spontaneous binding accelerated molecular dynamics simulation (aMD) of compound **15**. (a) Plot of the distance (left) between the γ carbon of Asp335 and the NH urea of compound **15** for a replica of 250 ns of conventional molecular dynamics (cMD) followed by 2000 ns of accelerated molecular dynamics (aMD) simulations. To reconstruct the spontaneous binding process, we placed one molecule of **15** in the solvent with a minimum distance of 25 Å from catalytic Asp335, which is the starting point of the cMD simulation. The grey dashed vertical line indicates where the aMD simulation starts. Overlay of the most populated cluster (right) obtained from 3 replicas of 500 ns of compound **15** (in cyan) bound in the active site using the docking orientation as starting pose with the inhibitor bound pose predicted from spontaneous binding aMD simulations (in purple).

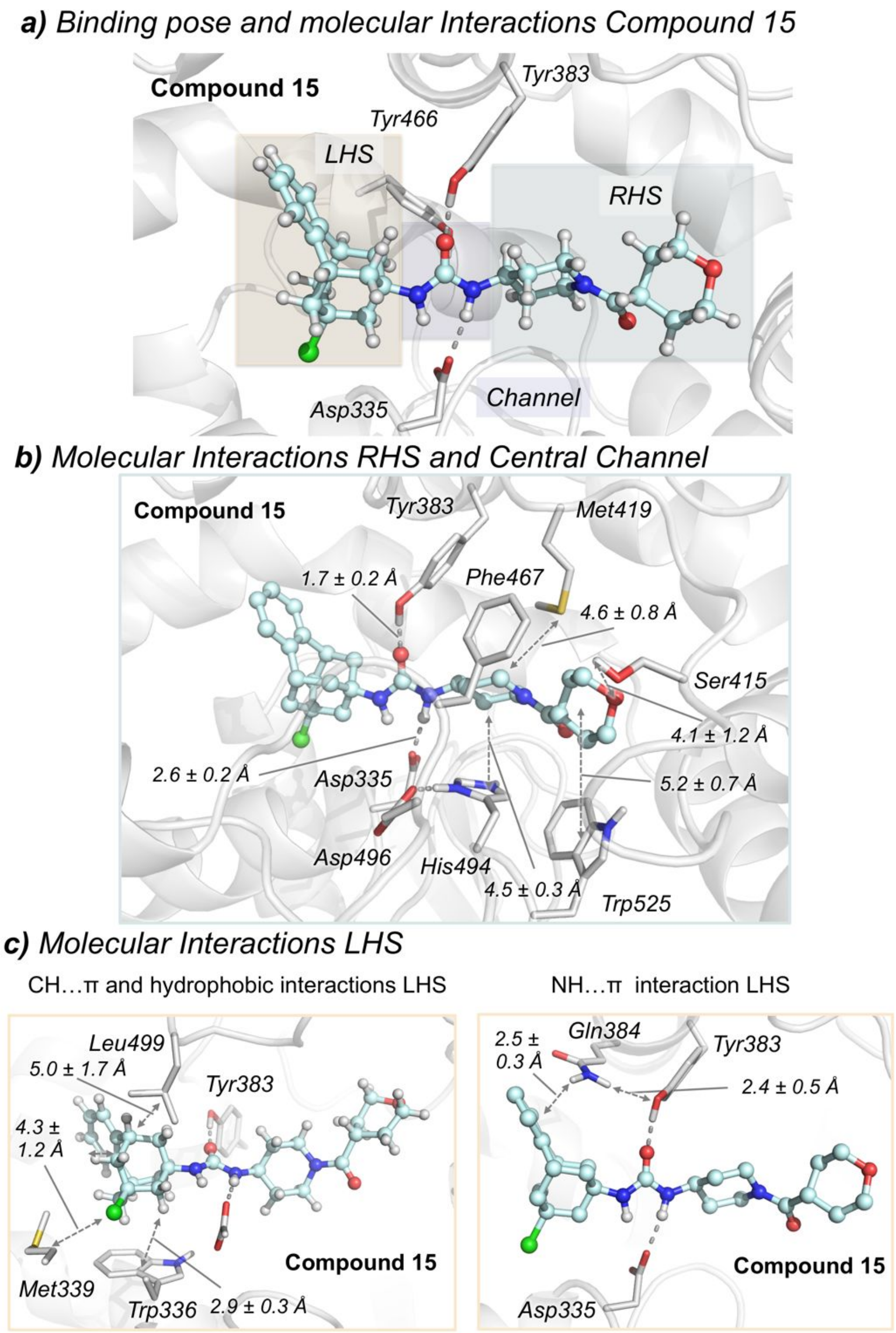

Figure S2. Binding pose and molecular interactions of compound **15**. (a) Representative binding pose of compound **15** bound in the active site of sEH obtained from the most visited conformations along molecular dynamics (MD) simulations. The benzohomoadamantane moiety occupies the LHS pocket while the piperidine group is placed in the RHS pocket. The central urea unit establishes hydrogen bonds with Asp335, Tyr466, and Tyr383. (b) Most relevant molecular interactions in the RHS. Average distances (in Å) obtained from three replicas of 500 ns of MD simulations are represented. Hydrogen bonds between the oxygens of the tetrahydropyran group of 21 and the hydrogen of the OH group of Ser415 is shown. The hydrophobic interaction average distances are computed between the terminal heavy atom of amino acid side chains and the centroid of each ring. Hydrogen bond distances between the carboxylic group of the catalytic Asp335 and the amide groups of the inhibitor and the distance between the

carbonyl group of the urea inhibitor and the OH group of Tyr383 and Tyr466 residues. (c) Most relevant molecular interactions in the LHS. Average distances (in Å) obtained from the three replicas of 500 ns of MD simulations are represented. The CH···π interaction is calculated between the hydrogens of the benzohomoadamantane unit and the centroid of the benzoid ring of Trp336. The NH···π interaction is monitored between the amide hydrogen of Gln384 and the center of the aromatic ring of the benzohomoadamantane scaffold.

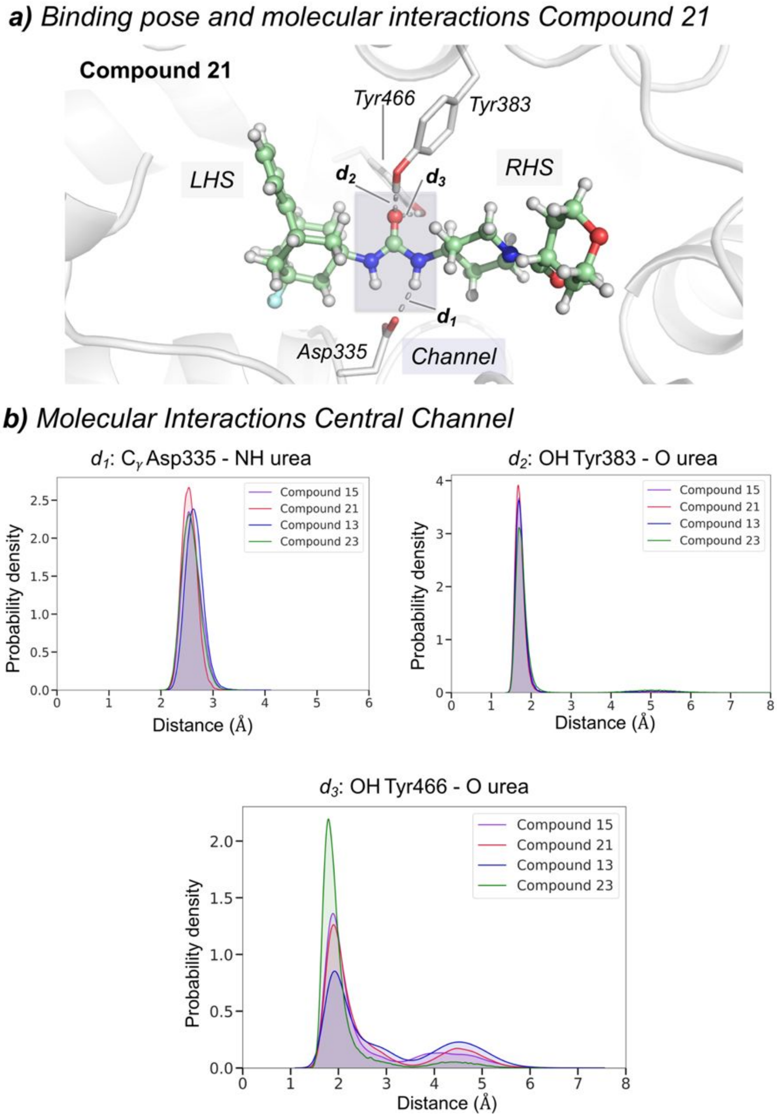

Figure S3. Binding pose and molecular interactions of compound **21**. (a) Representative binding pose of compound **21** bound in the active site of sEH obtained from the most visited conformations along the MD simulations. The benzohomoadamantane moiety occupies the LHS pocket while the benzoic acid group lays in the RHS pocket. The central urea unit establishes hydrogen bonds with Asp335 (**d$_1$**), Tyr466 (**d$_2$**), and Tyr383 (**d$_3$**). (b) Molecular interactions between compound 21 and residues of sEH central channel. Histogram plots of the distance between the carboxylic group of the catalytic Asp335 and the amide groups of the inhibitor [d_1(C_γAsp335- NH_{INH})], the distance between the carbonyl group of the urea inhibitor and the OH group of Tyr383 residue [d_2(OHTyr466-O_{INH})], and the distance between the carbonyl group of the urea inhibitor and the OH group of Tyr466 residue [d_3(OHTyr466-O_{INH})] along the MD simulations of **15** (purple), **21** (red), **13** (blue), and **23** (green).

a) Binding pose and molecular interactions Compound 13

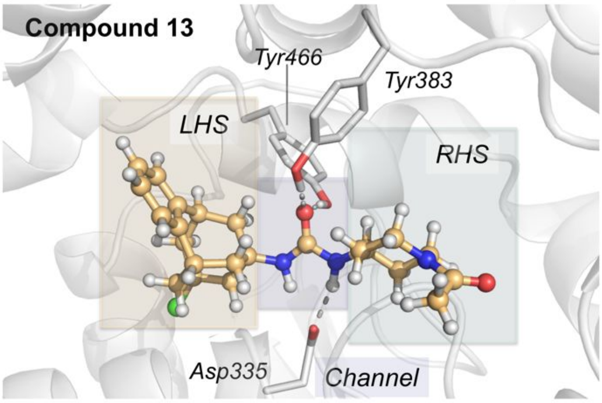

b) Molecular Interactions RHS and Central Channel Compound 13

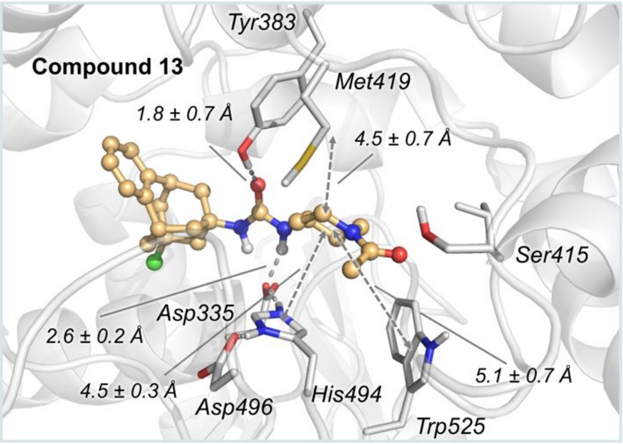

c) Molecular Interactions LHS Compound 13

CH…π and hydrophobic interactions LHS

NH…π interaction LHS

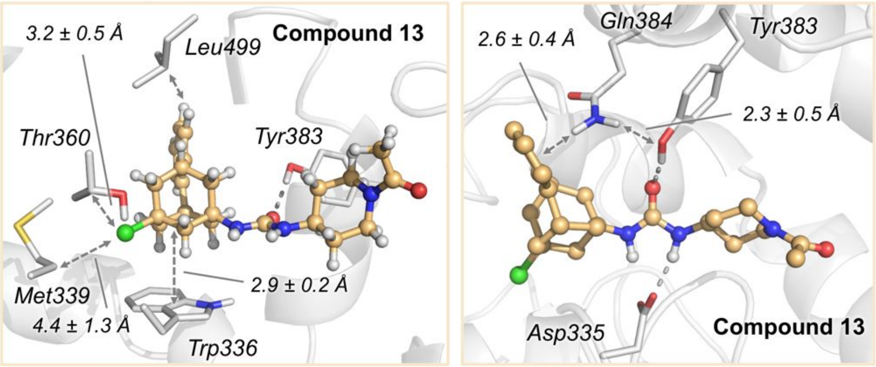

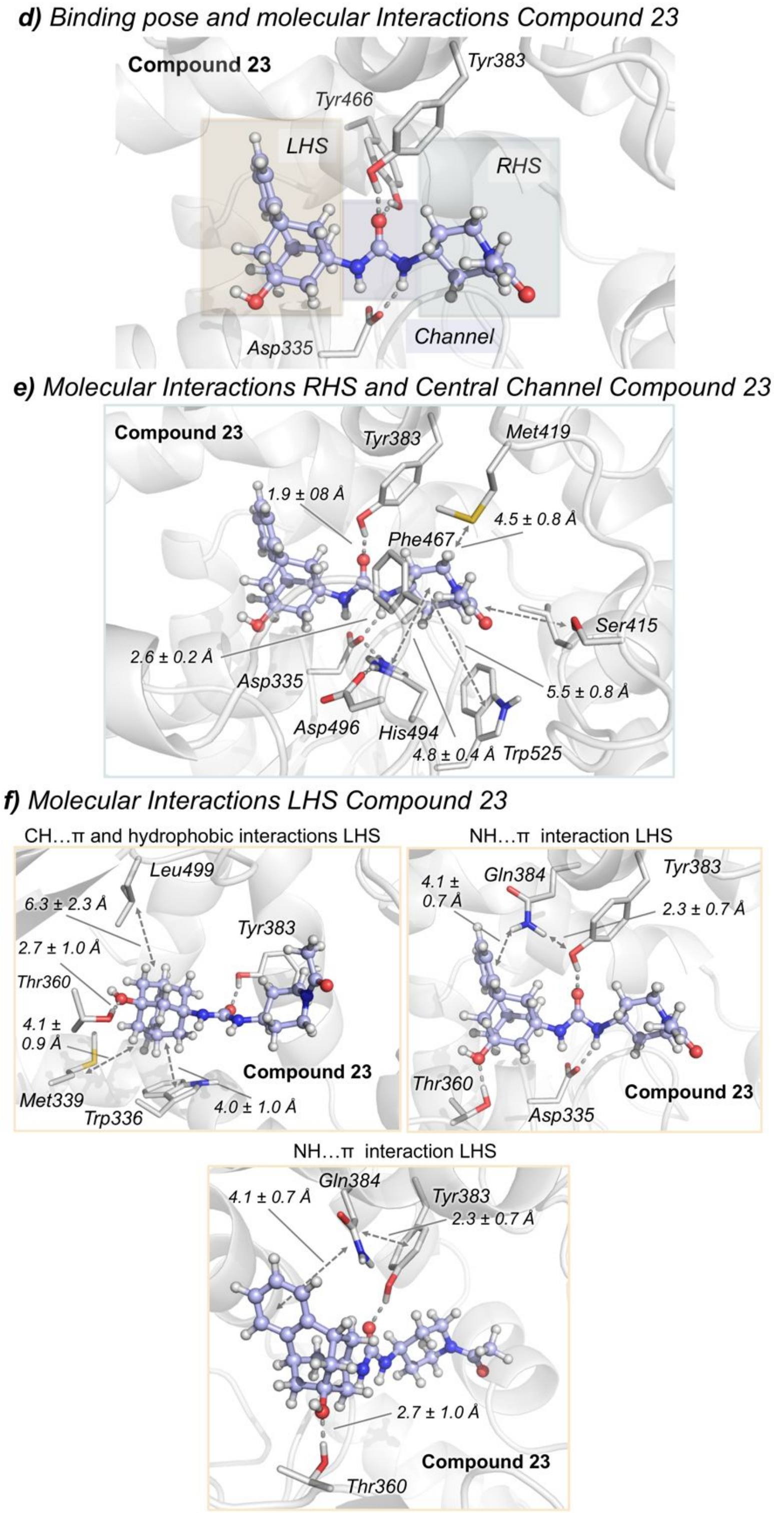

Figure S4. Binding pose and molecular interactions of compounds **13** and **23**. (a) Representative binding pose of compound **13** bound in the active site of sEH obtained from the most visited conformations along molecular dynamics (MD) simulations. (b) Most relevant molecular interactions of compound **13** in the RHS. (c) Most relevant molecular interactions of compound **13** in the LHS. Average distances (in Å) obtained from the three replicas of 500 ns of MD simulations are represented. (d) Representative binding pose of compound **23** bound in the active site of sEH obtained from the most visited conformations along molecular dynamics (MD) simulations. (e) Most relevant

molecular interactions of compound **23** in the RHS. (f) Most relevant molecular interactions of compound **23** in the LHS. In both compounds **13** and **23**, the benzohomoadamantane moiety occupies the LHS pocket while the piperidine group is placed in the RHS pocket. Average distances (in Å) obtained from three replicas of 500 ns of MD simulations are represented. The hydrophobic interaction average distances are computed between the terminal heavy atom of amino acid side chains and the centroid of each ring. Hydrogen bond distances between the carboxylic group of the catalytic Asp335 and the amide groups of the inhibitor and the distance between the carbonyl group of the urea inhibitor and the OH group of Tyr383 and Tyr466 residues. The CH···π interaction is calculated between the hydrogens of the benzohomoadamantane unit and the centroid of the benzoid ring of Trp336. The NH···π interaction is monitored between the amide hydrogen of Gln384 and the center of the aromatic ring of the benzohomoadamantane scaffold.

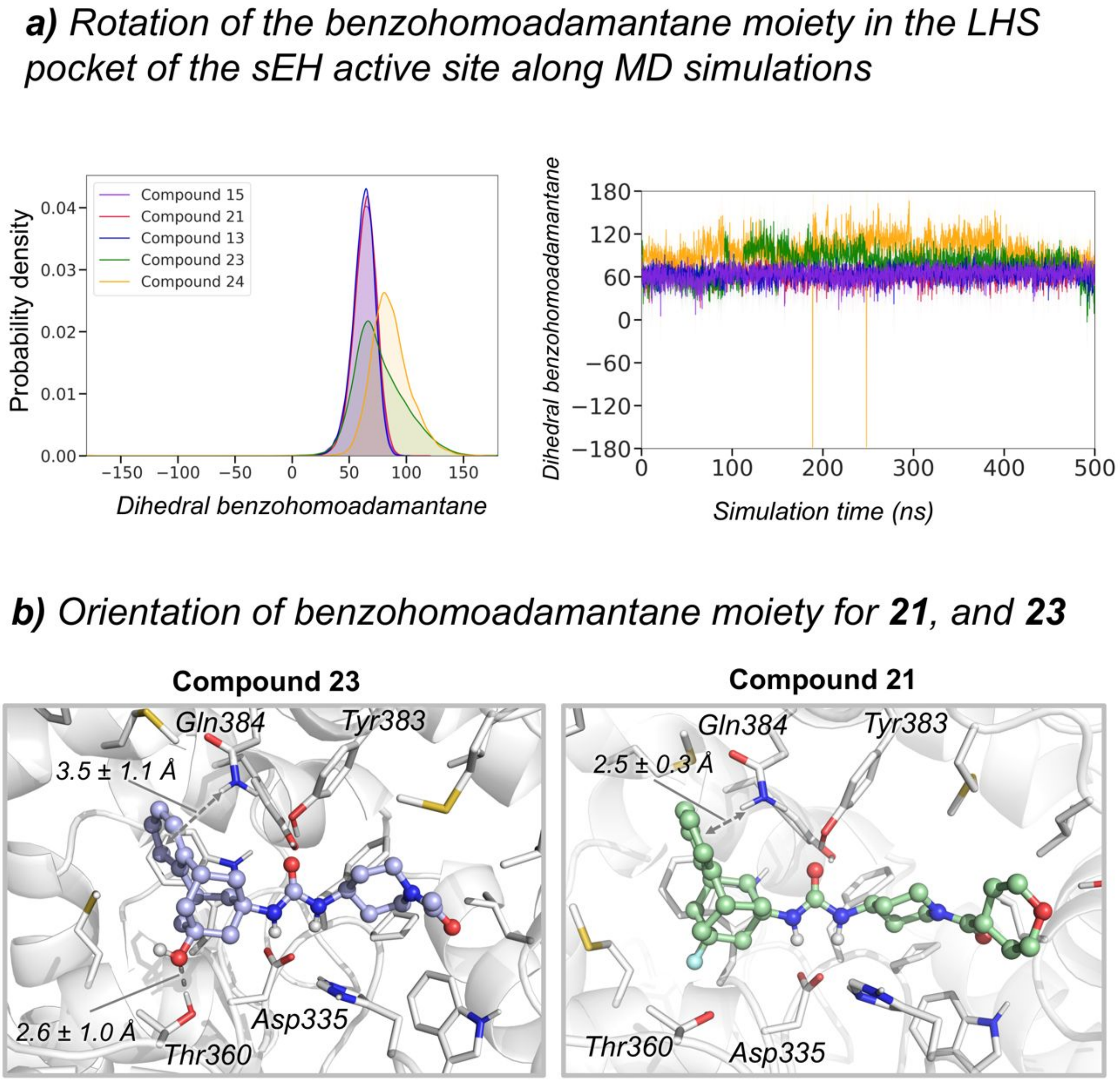

Figure S5. Rotation and orientation of the benzohomoadamantane moiety in the LHS pocket of sEH. (a) Histogram (left) and plot (right) of the dihedral angle that describes the rotation of the benzohomoadamantane moiety in the left-hand-side (LHS) pocket of the sEH active site along the MD simulations of **15** (purple), **21** (red), **13** (blue), **23** (green), and **24** (orange). (b) Structural comparision of benzohomoadamantane orientation in compounds **21** and **23**. The figure shows that the Thr360 side chain establishes a hydrogen bond with the oxygen of the hydroxyl substituent of compound **23** that induces the rotation of the benzohomoadamantane scaffold in the LHS pocket. This breaks the NH···π interaction between Gln384 and the aromatic ring of **23** flexibilizing the benzohomadamantane moiety.

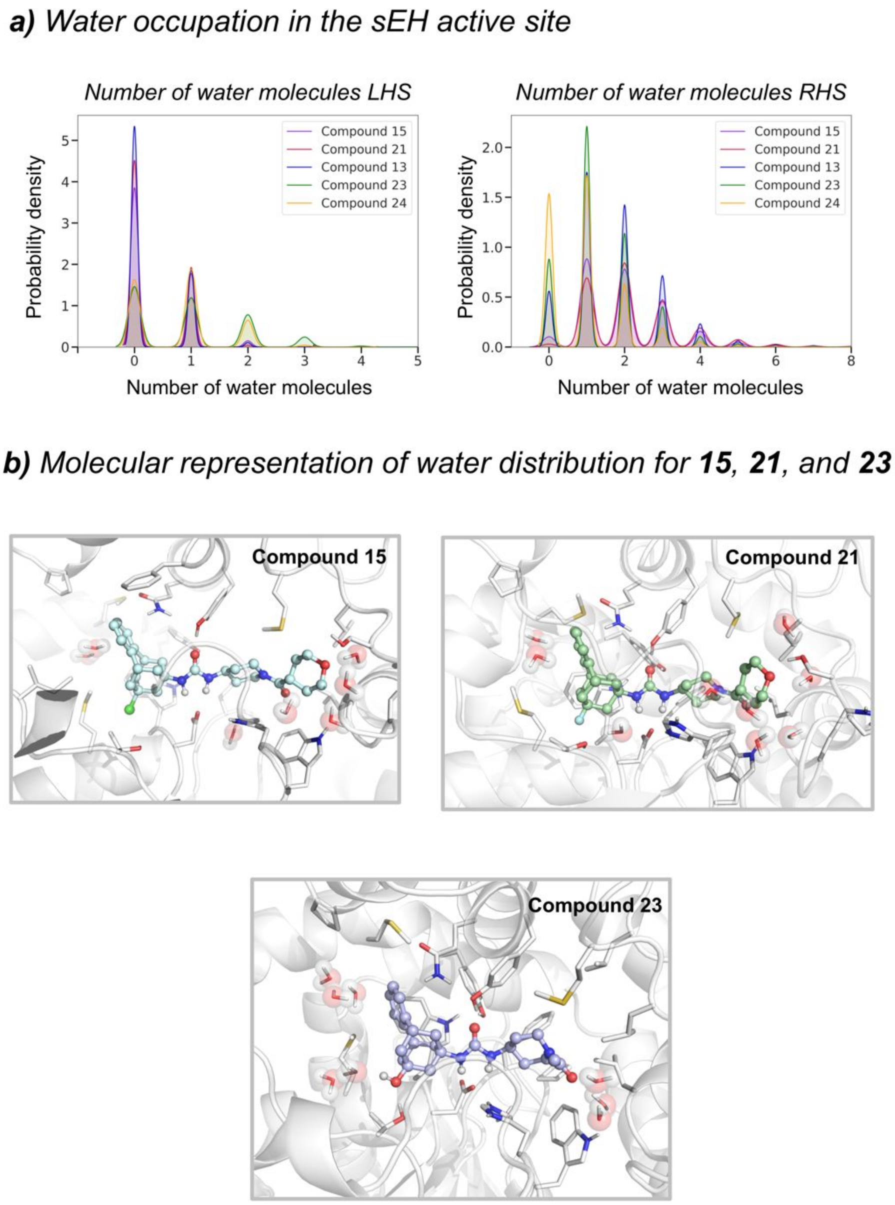

Figure S6. Water occupation in the sEH active site. (a) Representation of the normalized kernel density plot of the water distribution in the active site LHS and RHS pocket in the presence of **15** (purple), **21** (red), **13** (blue), **23** (green), and **24** (orange). We monitored the presence of water molecules through visual inspection of MD trajectories and using the watershell function of cpptraj MD analysis program. Using watershell, we calculated the number of water molecules in the first solvation shell (using a distance cutoff of 3.4 Å) for both RHS (piperidine group) and LHS (benzohomoadamantane) pockets along the MD simulations. The average number of water molecules in the LHS pocket is: 0.36 ± 0.54 for **15**, 0.31 ± 0.48 for **21**, 0.29 ± 0.49 for **13**, 0.97 ± 0.96 for **23**, and 0.59 ± 0.64 for **24**. The average number of water molecules in the RHS pocket is: 2.01 ± 1.25 for **15**, 2.24 ± 1.32 for **21**, 1.73 ± 1.21 for **13**, 1.33 ± 1.05 for **23**, and 0.96 ± 1.03 for **24**. (b) Representative MD snapshots displaying the water occupation sEH active site for compounds **15**, **21**, and **23**.

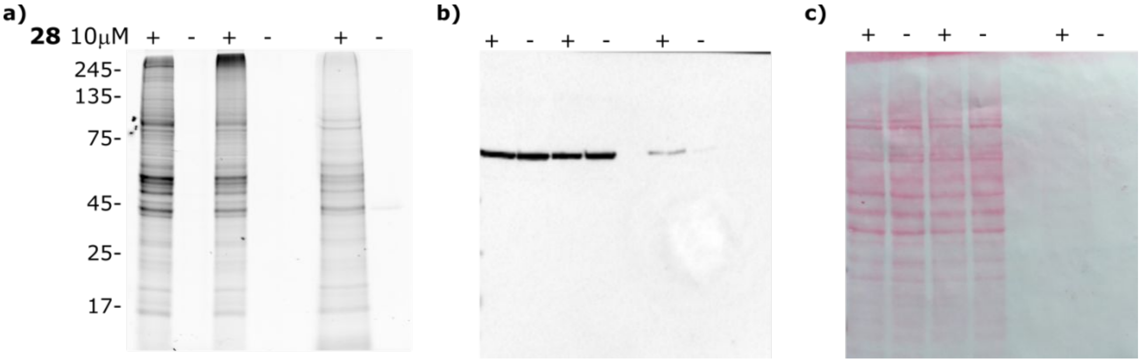

Figure S7. Target spectrum of probe **28** in HEK293T cells. Lanes from left to right: (1) input control treated with **28 (2)** input control with DMSO, (3) protein resuspension control after protein precipitation with acetone treated with **28,** (4) protein resuspension control treated with DMSO after protein precipitation with acetone, (5) proteins released from biotin beads treated with **28** and (6) proteins released from biotin beads treated with DMSO. Panels represent different read outs from the same experiment: **a)** Fluorescent scan which shows proteins labelled by probe **28**. **b)** Western blot analysis of proteins enriched by probe **28** shows soluble epoxide hydrolase as target. **c)** Ponceau stain shows equal protein loading in probe treated lanes and control lanes.

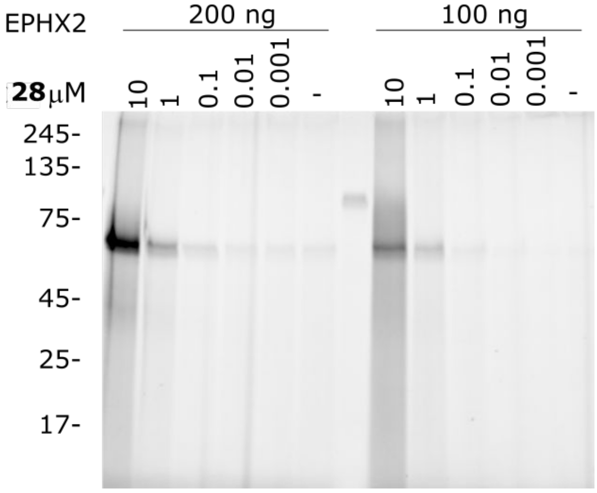

Figure S8. Fluorescence intensity of photoaffinity labeling of purified soluble epoxide hydrolase shows 10 nM to be the minimal probe concentration for efficient visualization of enzyme labeling.

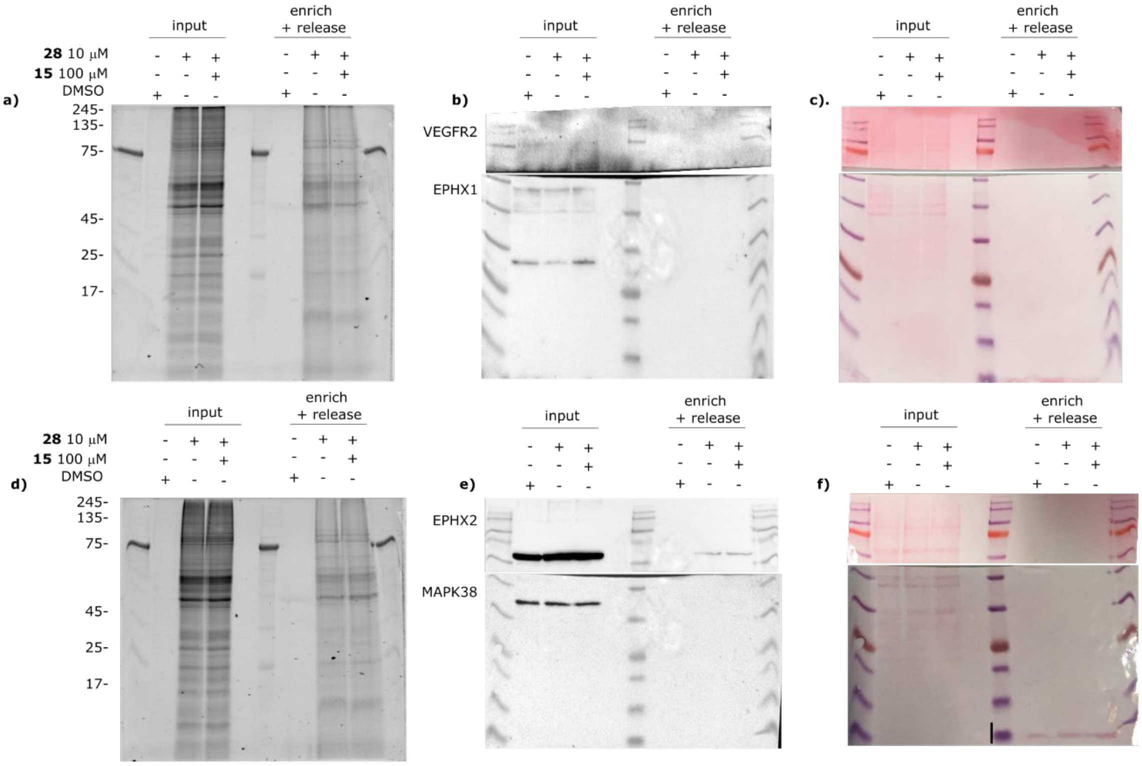

Figure S9. Pull down experiments to confirm off-targets of **15** using **28** at 10 μM reveals selective engagement to soluble epoxide hydrolase (EPHX2) over VEGFR2, MAPK38 and microsomal epoxide hydrolase (EPHX1). Experiments were performed in HEK293T cell lysates using probe **28** to which a biotin and TAMRA tag was clicked after irradiation. The bifunctional tag was used for fluorescent visualization of probe targets and enrichment and release using streptavidin beads. Panels show the fluorescent scan (**a)** and **d**), Western Blot (**b** and **e**) and Ponceau stain (**c** and **f**) of input control and released samples using either: DMSO, probe **28** or probe **28** in competition with **15** 100 μM treated sample. For immunoblotting the membranes were cut and incubated with relevant antibodies: VEGFR2 (top part **b**), EPHX1 (low part **b**), EPHX2 (top part **c**), MAPK38 (low part **c**).

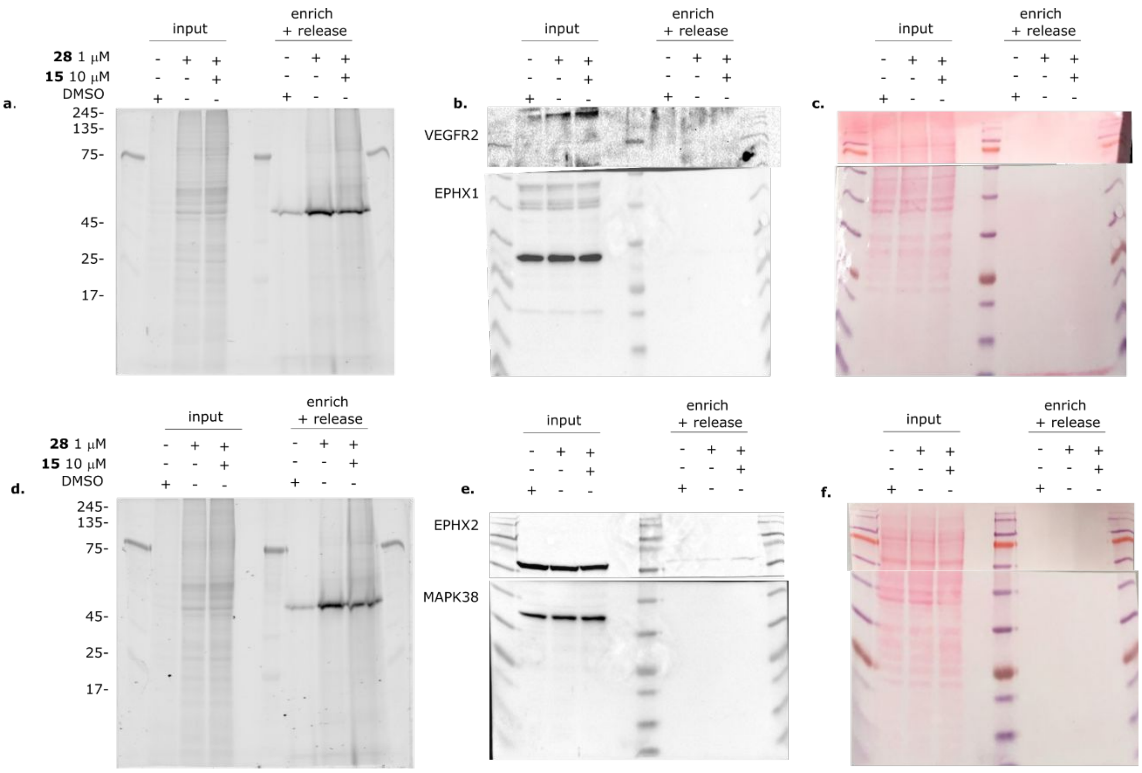

Figure S10. Pull down experiments to confirm off-targets of **15** using **28** at 1 μM reveals selective engagement to soluble epoxide hydrolase (EPHX2) over VEGFR2, MAPK38 and microsomal epoxide hydrolase (EPHX1). Experiments were performed in HEK293T cell lysates using probe **28** to which a biotin and TAMRA tag was clicked after irradiation. The bifunctional tag was used for fluorescent visualization of probe targets and enrichment and release using streptavidin beads. Panels show the fluorescent scan (**a** and **d**), Western Blot (**b** and **e**) and Ponceau stain (**c** and **f**) of input control and released samples using either: DMSO, **28** or **28** in competition with **15** 10 μM treated sample. For immunoblotting the membranes were cut and incubated with relevant antibodies: VEGFR2 (top part **b**), EPHX1 (low part **b**), EPHX2 (top part **c**), MAPK38 (low part **c**).

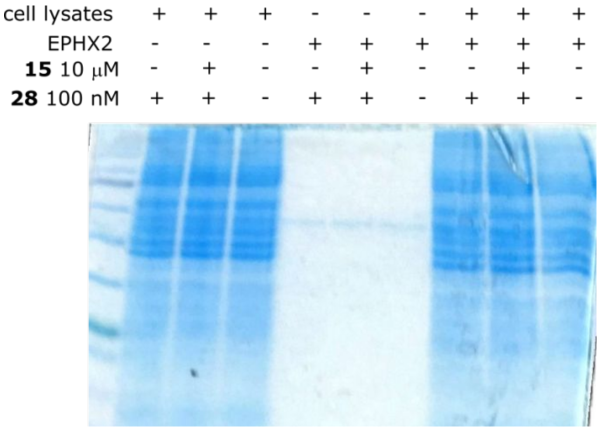

Figure S11. Coomassie-stained gel corresponding to the fluorescent scan of Figure 5 panel a.

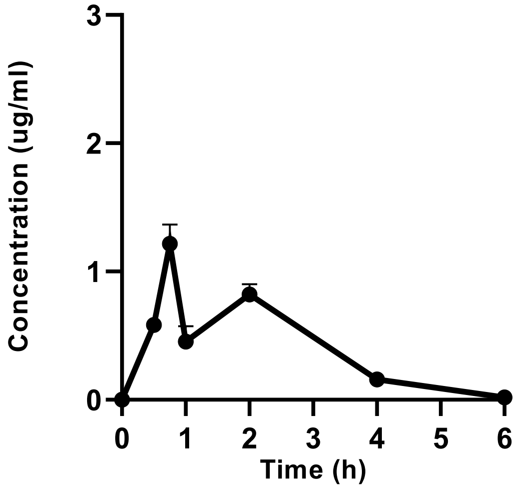

Figure S12. Plasma concentration *vs* time for compound **15** (5 mg/Kg, SC) in mouse.

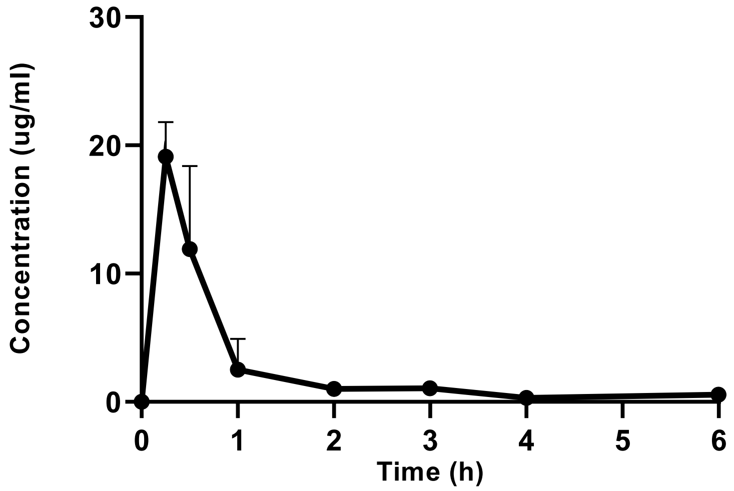

Figure S13. Plasma concentration *vs* time for compound **21** (5 mg/Kg, SC) in mouse.

Movie S1. Molecular movie of the spontaneous binding accelerated molecular dynamics simulation of compound **15** into the sEH active site.
